# Supplementary material for: Improving cascade outcomes for active TB: A global systematic review and meta-analysis of TB interventions
Source: PLoS Med. 2023 Jan 3;20(1):e1004091. doi: 10.1371/journal.pmed.1004091 (PMC9847969; doi:10.1371/journal.pmed.1004091)
Supplement: S1 File — Protocol A in S1 File. Study protocol as published on PROSPERO. Table B in S1 File. Search strategy and results. Table C in S1 File. Characteristics of included studies grouped by intervention type. Table D in S1 File. Results of meta-regression analysis. Table E in S1 File. Outcome of risk of bias assessment for 84 RCTs studies using the Cochrane Risk of Bias Assessment Tool. Table F in S1 File. Quality assessment of included studies using the EPHPP quality assessment tool. Table G in S1 File. GRADE Outcomes. Table H in S1 File. PRISMA checklist for protocol. (DOCX) [file pmed.1004091.s001.docx]

# Supplementary materials

[Supplementary materials 1](#_Toc122529627)

[Protocol A. 2](#_Toc122529628)

[Table B in S1File Showing Search Strategy and Results for Each Database Searched 7](#_Toc122529629)

[Table C in S1 File Showing Characteristics of Included Studies Grouped by Intervention Type. 8](#_Toc122529630)

[Table D in S1 File Showing Results of Meta-regression Analysis 20](#_Toc122529631)

[Table E in S1 File Showing Outcome of Risk of Bias Assessment for 84 RCTs Studies Using the Cochrane Risk of Bias Assessment Tool 22](#_Toc122529632)

[Table F in S1 File Showing Quality Assessment of Included Studies Using the EPHPP Quality Assessment Tool. 27](#_Toc122529633)

[Table G in S1 File Showing GRADE Outcomes 29](#_Toc122529634)

[Summary of GRADE Assessment of Quality of Evidence Certainty for each Outcome of interest for TB testing 29](#_Toc122529635)

[Summary of GRADE Assessment of Quality of Evidence Certainty for each Outcome of interest for TB Diagnosis 31](#_Toc122529636)

[Summary of GRADE Assessment of Quality of Evidence Certainty for each Outcome of interest for linkage-to-care 33](#_Toc122529637)

[Summary of GRADE Assessment of Quality of Evidence Certainty for each Outcome of interest for TB cure 37](#_Toc122529638)

[Summary of GRADE Assessment of Quality of Evidence Certainty for each Outcome of interest for treatment completion 40](#_Toc122529639)

[Summary of GRADE Assessment of Quality of Evidence Certainty for each Outcome of interest for treatment success 43](#_Toc122529640)

[Table H in S1 File PRISMA checklist for protocol 47](#_Toc122529641)

# Protocol A.

**Interventions to Optimize Care Continuum for Tuberculosis: A Systematic Review and Meta-analyses**

**INTRODUCTION**

Tuberculosis (TB) is one of the top leading causes of death worldwide, with an estimated 1.7 million deaths in the year 2016.^1^ In addition, more than 95% of these deaths occur in low- and middle-income countries.^2^ TB is also a leading killer of people living with HIV.^2^ For example, in 2016, 40% of HIV-related deaths were due to TB. As a curable and preventable disease, effective treatment can prevent the further transmission of TB and reduce TB-related mortality. Thus, identifying people with TB symptoms and linking them to care is a public health priority for TB control.^3^ Current therapies for active, drug-susceptible TB disease is treated with a standard 6-month course of 4 antimicrobial drugs that are provided with information, supervision, and support to the patient by a health worker or trained volunteer.^2^ When the treatment and care strategies are provided and taken properly, we can successfully cure the vast majority of TB cases.

Even curable, operational interventions that can improve the quality of care across a continuum of care for TB are strongly needed^4^. Identification of interventions that can maximize engagement and retention along the TB care continuum is essential for several reasons. However, most people living with active TB do not know their infection status and do not get tested until sick^5^, especially for people living in low-income and middle-income countries^6^. In addition, without interventions and support for a continuum of care, treatment adherence can be difficult and the disease can further spread^7^. Simple and inexpensive operational interventions could maximize the effect of limited health services in these low-resource settings, and some have shown efficacy in HBV, HCV, and HIV research, and similar methods could be used for TB care continuum^8^.

WHO has proposed the goal of End TB strategy by 2035 with a 95% reduction in TB death and a 90% reduction in tuberculosis incidence by 2035^9^. To achieve these ambitious targets, operational interventions that can optimize the delivery of TB services are needed. The purpose of this review was to synthesize data on operational interventions for TB testing, linkage to care, treatment uptake, adherence, and cure in adults.

| **Table A in S1 File Showing Details of PICO Steps to be used in Formulating Search Strategy** | |
| --- | --- |
| **PICO** |  |
| **P** | Individuals living with TB (diagnosed or undiagnosed) or providers caring for these patients. |
| **I** | Operational interventions delivered in conjunction with testing, care, or treatment of TB infection |
| **C** | Standard of care or no intervention |
| **O** | Retention and progression along the continuum of care |

Population: Tuberculosis OR TB

Intervention: Intervention; Counseling; Education OR educate; Teach; Training; Program; Engagement; Smoking AND reduce, reduction, cessation

Outcome: Screen OR screened OR screening; Test OR tested OR testing; Linking OR linkage; Refer OR referral; Uptake; Retain OR retained OR retention; Adherence OR adhere; Compliance OR comply

**METHOD:**

Search Strategy

Searches will be conducted in PubMed, Google Scholar, Ovid/MEDLINE, Global Health, and Embase using free text and controlled vocabulary terms (MeSH). The following study designs will be permitted: RCTs, Observational cohort studies, cross-sectional surveys, program evaluations. Modeling studies; qualitative studies, surveys examining predictors of having a test in general (willingness, knowledge, acceptability, etc.) will be excluded. A broad compound search strategy will be developed that combine terms for “TB”, “Intervention”, “Counseling”, “Education OR educate”, “Teach”, “Training”, “Program”, “Engagement”, “Alcohol AND reduce”, “Smoking AND reduce”, “reduction”, and “cessation”.

**PUBMED search strategy:**

(Tuberculosis OR TB[tiab]) AND (Intervention[tiab] OR counseling[tiab] OR education[tiab] OR educate[tiab] OR teach[tiab] OR training[tiab] OR program[tiab] OR Engagement[tiab] OR (alcohol [tiab] AND (reduce OR reduction OR cessation OR decrease)) OR (smoking[tiab] AND (reduce OR reduction OR cessation OR decrease))) AND (Uptake[tiab] OR Adherence[tiab] OR adhere[tiab] OR Compliance[tiab] OR comply[tiab] OR retain[tiab] OR retained[tiab] OR Retention[tiab] OR Screen[tiab] OR screened[tiab] OR screening[tiab] OR test[tiab] OR tested[tiab] OR testing[tiab] OR Linkage[tiab] OR linking[tiab] OR refer[tiab] OR Referral[tiab])

**Inclusion and exclusion criteria**

Authors of included abstracts will be contacted to determine whether the same data had been later published as a full-text article in a peer-reviewed journal, in which case the abstract would be excluded, and the full-text article included.

Study selection will proceed in three stages. First, two reviewers will screen titles obtained from the initial search strategy according to standard inclusion and exclusion criteria. Second, abstracts for all titles identified for further review will be assessed independently by two reviewers for inclusion. If there are disagreements, a third reviewer will determine final inclusion. Finally, full texts for all abstracts identified for further review will be assessed independently by two reviewers for inclusion. If there are disagreements, a third reviewer will determine final inclusion.

**Inclusion Criteria:**

- Peer-reviewed article or published abstract or registered clinical trial

- Non-pharmaceutical operational intervention

- Adults with diagnosed or undiagnosed TB

- Primary or secondary outcome was testing, linkage to care, treatment uptake, Linkage-to-care, treatment completion, treatment outcome, or disease endpoints

- Study design with comparator or control

**Exclusion Criteria:**

- Dissertation

- Pediatric populations

- Systematic reviews

- Studies not reporting necessary data

- Duplicate data of another included study

**Data extraction**

Data extraction will be completed by three independent reviewers using a standardized data extraction form. Data extraction categories will include the following: title, journal, first author, year of publication, year of study, study design, intervention type, treatment cascade, study population, HIV status, inclusion criteria, exclusion criteria, study location/country, study context, study settings, describe the intervention, intervention type, describe control, duration of intervention, blinding present, intent to treat analysis, sample in intervention, sample size in control, participant characteristics (intervention), participant characteristics (control), significant baseline differences, primary outcomes, secondary outcomes, intervention group (x/y, %), control group (x/y, %), RR, P-value, loss to follow up (intervention), loss to follow up (control), conclusion, comments.

The first table will summarize findings for quantitative analysis and GRADE quality of evidence assessment for each included article. The second table will include the characteristics of each of the included articles. A third table will focus on the quality assessment of included studies. Fig 1 will summarize the flowchart of the review, Fig 2 will provide the pooled results for results from each stage of the care continuum.

**Quality assessment**

In an analysis of quality assessment, studies will be stratified based on study design and level of evidence. Bias among randomized controlled studies will be assessed using the Cochrane Collaboration “Risk of Bias” tool, using six criteria in four sources of bias including selection bias, performance and detection bias, attrition bias, and reporting bias. Bias in observational studies will be assessed using the Newcastle-Ottawa Quality Assessment (EPHPP) Scale, which assesses selection bias, patient-level barrier, and measurement bias. The EPHPP tool assessed each study in seven main domains (selection bias, study design, confounders, blinding, data collection, methods, and withdrawals and dropouts of patients) and rated each aspect as strong, moderate, or weak quality. The quality of evidence will be assessed according to the methodology described by the GRADE working group. A GRADE table will be generated for each meta-analysis and sub-analysis.

**Data analysis**

All included publications will be assessed for comparability based on intervention type, control condition, and outcome. Studies determined to be similar for intervention, control, and the outcome will be included in meta-analyses to determine pooled effect sizes. Pooled relative risks or odds ratios with confidence intervals and forest plots were generated using a random-effects model in Review Manager 5.3. The heterogeneity between studies in comparison was assessed by calculating *I^2^*.

Meta-analyses that included both RCTs and NRSs will be stratified by study design. If possible, the pooled results will be reported as relative risk. When studies only reported odds ratios with confidence intervals, data will be pooled using the generic inverse variance method, and a pooled odds ratio will be reported. Funnel plots will be used to screen for reporting bias only if ten or more studies were included in a meta-analysis.

Whenever possible, we will stratify the participants into HIV+/HIV-, and conduct a sub-analysis among these two groups of people.

**References**

1. Organization WH. Global tuberculosis report 2016. 2016.

2. Organization WH. Tuberculosis Fact Sheet. Geneva, 2016.

3. Organization WH. WHO policy on TB infection control in health-care facilities, congregate settings, and households. 2009.

4. Wingfield T, Boccia D, Tovar MA, et al. Designing and implementing a socioeconomic intervention to enhance TB control: operational evidence from the CRESIPT project in Peru. BMC Public Health 2015; 15(1): 810.

5. Padmapriyadarsini C, Narendran G, Swaminathan S. Diagnosis & treatment of tuberculosis in HIV co-infected patients. The Indian journal of medical research 2011; 134(6): 850.

6. Pai NP, Vadnais C, Denkinger C, Engel N, Pai M. Point-of-care testing for infectious diseases: diversity, complexity, and barriers in low-and middle-income countries. PLoS medicine 2012; 9(9): e1001306.

7. Munro SA, Lewin SA, Smith HJ, Engel ME, Fretheim A, Volmink J. Patient adherence to tuberculosis treatment: a systematic review of qualitative research. PLoS medicine 2007; 4(7): e238.

8. Zhou K, Fitzpatrick T, Walsh N, et al. Interventions to optimize the care continuum for chronic viral hepatitis: a systematic review and meta-analyses. The Lancet infectious diseases 2016; 16(12): 1409-22.

9. Uplekar M, Weil D, Lonnroth K, et al. WHO's new End TB Strategy. The Lancet 2015; 385(9979): 1799-80

| Table B in S1File Showing Search Strategy and Results for Each Database Searched | | |
| --- | --- | --- |
| PubMed | | |
| **Search** | **Query** | **Results** |
| 1 | "Tuberculosis, meningeal"[MeSH Terms] OR "Tuberculosis"[TW] OR "TB"[TW] | 291,702 |
| 2 | Uptake[tiab] OR Adherence[tiab] OR adhere[tiab] OR Compliance[tiab] OR comply[tiab] OR compliant[tiab] OR retain[tiab] OR retained[tiab] OR Retention[tiab] OR outcome[tiab] OR outcomes[tiab] OR Testing[tiab] OR Diagnosis[tiab] OR Diagnostics[tiab] OR linkage-to-care[tiab] OR Linkage to care[tiab] | 5,250,867 |
| 3 | intervention[tiab] OR interventions[tiab] OR interventional[tiab] OR cohort*[tiab] OR trial[tiab] OR trials[tiab] OR RCT [tiab] | 2,451,417 |
| #4 | #1 AND #2 AND #3 | 8,515 |
| Embase | | |
| **1** | tuberculosis:de,ab,ti OR tb:de,ab,ti | 342,377 |
| **2** | (Uptake OR Adherence OR adhere OR Compliance OR comply OR compliant OR retain OR retained OR Retention OR outcome OR outcomes OR testing OR diagnosis OR diagnostics OR Linkage-to-care OR Linkage to care): ab,ti | 937,198 |
| **3** | (Intervention OR interventions OR interventional OR cohort* OR trial OR trials OR RCT): ab,ti | 4,259,297 |
| **4** | #1 AND #2 AND #3 | 3,596 |
| CINAHL | | |
| **S1** | TX (tb or tuberculosis) | 70,781 |
| **S2** | Uptake OR Adherence OR adhere OR Compliance OR comply OR compliant OR retain OR retained OR Retention OR outcome OR outcomes OR testing OR diagnosis OR diagnostics OR Linkage-to-care OR Linkage to care | 2,314,100 |
| **S3** | Intervention OR interventions OR interventional OR cohort* OR trial OR trials OR RCT | 1,216,846 |
| **S4** | S1 AND S2 AND S3 | 6,860 |
| PsycINFO | | |
| **S1** | TX (tb or tuberculosis) | 5,059 |
| **S2** | Uptake OR Adherence OR adhere OR Compliance OR comply OR compliant OR retain OR retained OR Retention OR outcome OR outcomes OR testing OR diagnosis OR diagnostics OR Linkage-to-care OR Linkage to care | 1,174,566 |
| **S3** | Intervention OR interventions OR interventional OR cohort* OR trial OR trials OR RCT | 761,038 |
| **S4** | S1 AND S2 AND S3 | 732 |
| Cochrane Trials | | |
| **S1** | (tuberculosis OR TB): ti,ab,kw | 8594 |
| **S2** | (Uptake OR Adherence OR adhere OR Compliance OR comply OR compliant OR retain OR retained OR Retention OR outcome OR outcomes OR testing OR diagnosis OR diagnostics OR Linkage-to-care OR Linkage to care): ti,ab,kw | 1,063,390 |
| **S3** | (Intervention OR interventions OR interventional OR cohort* OR trial OR trials OR RCT): ti,ab,kw | 1,244,756 |
| **S4** | S1 AND S2 AND S3 | 4587 |

# Table C in S1 File Showing Characteristics of Included Studies Grouped by Intervention Type.

| **Refs** | **Study** | **Countries** | **Region** | **Study design** | **Study design** | **Integration with HIV** | **Intervention** |
| --- | --- | --- | --- | --- | --- | --- | --- |
|  | **TB testing** |  |  |  |  |  |  |
| [1] | Aldridge2015 | United Kingdom | 2 | Cluster RCT | 1 | No | Education & Counselling |
| [2] | Bello2017 | Malawi | 1 | cluster randomized interventional trial | 1 | Yes | Staff Training |
| [3] | Chaisson1996 | United States | 2 | non-RCT | 2 | Yes | Education & Counselling, Incentives |
| [4] | Churchyard2011 | South Africa | 1 | RCT | 1 | No | Active Case Finding |
| [5] | Durovni2013 | Brazil | 1 | Cluster RCT | 1 | Yes | Education & Counselling |
| [6] | Ekwueme2014 | Nigeria | 1 | non-RCT | 2 | No | Education & Counselling |
| [7] | Fitzgerald 1999 | Canada | 2 | pre and post intervention study | 2 | No | Incentives |
| [8] | Griffiths2007 | United Kingdom | 2 | Cluster RCT | 1 | No | Education Outreach |
| [9] | Harstad 2014 | Norway | 2 | non-RCT | 2 | Yes | Collaboration |
| [10] | Malotte1999 | United States | 2 | non-RCT | 2 | No | Education & Counselling, Incentives |
| [11] | Sequeira-Aymar2021 | Spain | 2 | Cluster RCT | 1 | No | Digital Health |
| [12] | Shah 2020 | Peru | 1 | RCT | 1 | No | Community Case-Finding |
| [13] | Simwaka2012 | Malawi | 1 | pre and post intervention study | 2 | No | Community Case-Finding |
| [14] | Uwimana2012 | South Africa | 1 | Cluster RCT | 1 | Yes | Community Case-Finding |
| [15] | Uwimana2013 | South Africa | 1 | before and after intervention study | 2 | Yes | Community Case-Finding |
| [16] | Vo2020 | Vietnam | 1 | controlled intervention study | 2 | No | Community Case-Finding |
| [17] | Volkmann2016 | Kenya | 1 | pre and post intervention study | 2 | No | Community Case-Finding |
| [18] | Yassi2016 | South Africa | 1 | Cluster RCT | 1 | Yes | Digital Health |
| [19] | Yellappa2018 | India | 1 | RCT | 1 | No | Collaboration |
| [20] | Zaeh2013 | Ethiopia | 1 | pre and post intervention study | 2 | Yes | Education &Counselling |
|  | **TB diagnosis** |  |  |  |  |  |  |
| [21] | Alisjahbana2005 | Indonesia | 1 | RCT | 1 | No | Sputum Sampling Instructions |
| [22] | Bai2008 | China | 1 | quasi-experimental study | 2 | No | Improve Facility TB Care |
| [23] | Balakrishnan2021 | India | 1 | Pre and post intervention study | 2 | No | Collaboration |
| [24] | Becx2001 | Indonesia | 1 | non-RCT | 2 | No | Community Case-Finding |
| [25] | Bjerrum2016 | Ghana | 1 | pre and post intervention study | 2 | Yes | Improve Facility TB Care |
| [26] | Calligaro2017 | Zimbabwe, South Africa | 1 | Multicenter randomized control trial | 1 | Yes | Improve Diagnose Technology And Efficiency |
| [4] | Churchyard2011 | South Africa | 1 | RCT | 1 | No | Active Case Finding |
| [27] | Corbett2010 | Zimbabwe | 1 | Cluster RCT | 1 | Yes | Community Case-Finding |
| [28] | Datiko2009 | Southern Ethiopia | 1 | Cluster RCT | 1 | No | Community Case-Finding |
| [29] | Dudley2003 | South Africa | 1 | non-RCT | 2 | No | Community Case-Finding |
| [30] | Durovni2014 | Brazil | 1 | Cluster RCT | 1 | No | Improve Diagnose Technology And Efficiency |
| [31] | Eom2022 | South Korea | 2 | RCT | 1 | No | Improve Diagnose Technology And Efficiency |
| [32] | Fairall 2005 | South Africa | 1 | Cluster RCT | 1 | No | Education Outreach |
|  | Fatima2016 | Pakistan | 1 | pre and post intervention study | 2 | No | Improve Diagnose Technology And Efficiency |
| [33] | Garg2020 | India | 1 | Quasi-experimental design. | 2 | No | Community Case-Finding |
| [34] | Geldenhuys2014 | South Africa | 1 | non-RCT | 2 | Yes | Sputum Sampling Instructions |
| [35] | Gengiah2021 | South Africa | 1 | Cluster randomized control trial | 1 | Yes | Mixed Interventions |
| [36] | Kaswaswa2022 | Malawi | 1 | Cluster RCT | 1 | No | Active Case Finding |
| [37] | Khan2007 | Pakistan | 1 | RCT | 1 | No | Sputum Sampling Instructions |
| [38] | Khan2016 One | Pakistan | 1 | RCT | 1 | No | Education & Counselling |
| [39] | Khan2016 Two | Pakistan | 1 | quasi-experimental study | 2 | No | Improve Facility TB Care |
| [40] | Lisboa2020 | Mozambique | 1 | Quasi- experimental | 2 | No | Mixed Interventions |
| [41] | MacPherson2021 | Malawi | 1 | RCT | 1 | Yes | Digital Health |
| [42] | Martinson2022 | South Africa | 1 | RCT | 1 | No | Active Case Finding |
| [43] | Mhalu2015 | Tanzania | 1 | RCT | 1 | No | Sputum Sampling Instructions |
| [44] | Parija2014 | India | 1 | pre and post intervention study | 2 | No | Community Case-Finding |
| [45] | Qureshi2010 | India | 1 | non-RCT | 2 | No | Improve Facility TB Care |
| [46] | Rudolf2021 | Guinea-Bissau/ Ethiopia | 1 | RCT | 1 | No | Improve Diagnose Technology and Efficiency |
| [47] | Rüütel2011 | Estonia | 1 | RCT | 1 | Yes | Improve Facility TB Care |
| [48] | Sah2021 | Nepal | 1 | pre and post intervention study | 2 | No | Active Case Finding |
| [13] | Simwaka2012 | Malawi | 1 | pre and post intervention study | 2 | No | Collaboration |
| [49] | Thu 2019 | Vietnam | 1 | Mixed methods | 2 | No | Collaboration |
| [50] | Timire2018 | Zimbabwe, Southern African | 1 | pre and post intervention study | 2 | No | Sputum Sampling Instructions |
| [51] | Wang 2009 | China | 1 | pre and post intervention study | 2 | No | Collaboration |
| [52] | Wei2015 | China | 1 | quasi-experimental study | 2 | No | Improve Facility TB Care |
| [18] | Yassi2016 | South Africa | 1 | Cluster RCT | 1 | Yes | Digital Health |
| [53] | Yassin2013 | Ethiopia | 1 | pre and post intervention study | 2 | No | Community Case-Finding |
|  | **Linkage to care** |  |  |  |  |  |  |
| [54] | Adane2019 | Ethiopia | 1 | Cluster randomized control trial | 1 | Yes | Education And Counselling |
| [55] | Al-Sahafi2021 | Saudi Arabia | 1 | RCT | 1 | No | Community-Based Interventions |
| [56] | Bassett2016 | South Africa | 1 | RCT | 1 | Yes | Patient Navigators and Referral |
| [4] | Churchyard2011 | South Africa | 1 | RCT | 1 | No | Active Case Finding |
| [5] | Durovni2013 | Brazil | 1 | Cluster RCT | 1 | Yes | Staff Training |
| [32] | Fairall 2005 | South Africa | 1 | Cluster RCT | 1 | No | Staff Training |
| [35] | Gengiah2021 | South Africa | 1 | Cluster randomized control trial | 1 | Yes | Mixed Interventions |
| [57] | Jenum2018 | India | 1 | Cluster randomized control trial | 1 | No | Active Case Finding |
| [36] | Kaswaswa2022 | Malawi | 1 | Cluster RCT | 1 | No | Active Case Finding |
| [58] | Lee2011 | Bangladesh | 1 | RCT | 1 | No | Mixed Interventions |
| [41] | MacPherson2021 | Malawi | 1 | RCT | 1 | Yes | Digital Health |
| [59] | Majella2021 | India | 1 | RCT | 1 | No | Reminders And Tracers |
| [60] | Mohan2003 | Iraq | 1 | RCT | 1 | No | Home-Based Care |
| [61] | Mukoka2022 | Malawi | 1 | RCT | 1 | Yes | Improved Facility TB Care |
| [62] | Mwansa-Kambafwile2022 | South Africa | 1 | RCT | 1 | No | Community-Based Active Case Finding, Reminders and Tracers |
| [14] | Uwimana2012 | South Africa | 1 | Cluster RCT | 1 | Yes | Staff Training |
| [63] | Wagstaff 2019 | South Africa | 1 | RCT | 1 | Yes | Digital Health |
| [64] | White1998 | United States | 2 | RCT | 1 | No | Incentives |
| [65] | Wingfield2017 | Peru | 1 | RCT | 1 | No | Incentives |
| [18] | Yassi2016 | South Africa | 1 | Cluster RCT | 1 | Yes | Mixed Interventions |
| [19] | Yellappa2018 | India | 1 | RCT | 1 | No | Collaboration Between Different Sectors |
|  | **Cured** |  |  |  |  |  |  |
| [55] | Al-Sahafi2021 | Saudi Arabia | 1 | randomized control trial | 1 | No | Community-Based Interventions |
| [66] | Ali2020 | Sudan | 1 | controlled intervention pilot study | 2 | No | SMS Reminder |
| [67] | Müller2019 | Brazil | 1 | RCT | 1 | No | Education |
| [68] | Al-Sayili2021 | Saudi Arabia | 1 | RCT | 1 | No | Community-Based Interventions |
| [69] | Awaisu2011 | Malaysia | 1 | non-RCT | 2 | No | Alcohol And Tobacco Control |
| [70] | Baluku2021 | Uganda | 1 | quasi-experimental study | 2 | No | Incentives |
| [24] | Becx2001 | Indonesia | 1 | non-RCT | 2 | No | Community-Based Intervention |
| [71] | Bediang2018 | Cameroon | 1 | RCT | 1 | No | Digital Health |
| [72] | Broomhead2012 | South Africa | 1 | non-RCT | 2 | No | Digital Health |
| [28] | Datiko2009 | Southern Ethiopia | 1 | Cluster RCT | 1 | No | Community Based Interventions |
| [73] | Datiko2017 | Southern Ethiopia | 1 | pre and post intervention study | 2 | No | Community Based Interventions |
| [74] | Diaw2018 | Senegal | 1 | pre and post intervention study | 2 | No | Education and Counseling, New Testing Tools / Strategies |
| [29] | Dudley2003 | South Africa | 1 | non-RCT | 2 | No | Community-Based Intervention |
| [75] | Farooqi2017 | Pakistan | 1 | RCT | 1 | No | Digital Health |
| [76] | Fatima2022 | Pakistan | 1 | RCT | 1 | No | Community-Based Interventions |
| [77] | Janmeja2005 | India | 1 | RCT | 1 | No | Psychotherapy |
| [78] | John2015 | Nigeria | 1 | pre and post intervention study | 2 | No | Active Case Finding |
| [79] | Khan2011 | Pakistan | 1 | pre and post intervention study | 2 | No | Improve Facility TB Care |
| [80] | Khortwong2013 | Thailand | 1 | pre and post intervention study | 2 | No | Education And Counselling |
| [81] | Kunawararak2011 | Thailand | 1 | RCT | 1 | No | Digital Health |
| [82] | Lee2015 | Bangladesh | 1 | quasi-experimental study | 2 | No | Incentives |
| [83] | Lewin2005 | South Africa | 1 | RCT | 1 | No | Staff Training |
| [84] | Louwagie2022 | South Africa | 1 | RCT | 1 | Yes | Alcohol And Tobacco Control |
| [85] | Lutge2013 | South Africa | 1 | Cluster RCT | 1 | No | Incentives |
| [86] | Manyazewal2022 | Ethiopia | 1 | RCT | 1 | No | Reminders And Tracers |
| [87] | Miti2003 | Zambia | 1 | non-RCT | 2 | Yes | Home-Based Care |
| [88] | Mohammed2016 | Pakistan | 1 | RCT | 1 | No | Digital Health |
| [89] | Niazi2003 | Iraq | 1 | RCT | 1 | No | Community-Based Intervention |
| [90] | Peltzer2013 | South Africa | 1 | Cluster RCT | 1 | No | Alcohol And Tobacco Control |
| [91] | Puchalski2015 | Malawi | 1 | Cluster RCT | 1 | No | Staff Training, Mixed Interventions |
| [88] | Mohammed2016 | Pakistan | 1 | RCT | 1 | No | Digital Health |
| [92] | Taneja 2017 | India | 1 | quasi-experimental study | 2 | No | Home-Based Care |
| [93] | Thekkur 2019 | India | 1 | Mixed methods | 2 | Yes | Digital Health, Integrated with HIV Care |
| [94] | Thiam 2007 | Senegal | 1 | Cluster RCT | 1 | No | Mixed Interventions |
| [50] | Timire2018 | Zimbabwe, Southern African | 1 | pre and post intervention study | 2 | No | Improved Facility TB Care |
| [95] | Torrens2016 | Brazil | 1 | pre and post intervention study | 2 | No | Incentives |
| [96] | Venkatapraveen2012 | Gulbarga | 1 | RCT | 1 | No | Staff Training |
| [65] | Wingfield2017 | Peru | 1 | RCT | 1 | No | Incentives |
| [97] | Wongduan2015 | Thailand | 1 | RCT | 1 | No | Mixed Interventions |
| [53] | Yassin2013 | Ethiopia | 1 | pre and post intervention study | 2 | No | Community-Based Intervention |
|  | **Treatment completion** | |  |  |  |  |  |
| [55] | Al. Sahafi2021 | Saudi Arabia | 1 | randomized control trial | 1 | No | Community-Based Interventions |
| [68] | Al-Sayali2021 | Saudi Arabia | 1 | RCT | 1 | No | Community-Based Interventions |
| [69] | Awaisu2011 | Malaysia | 1 | non-RCT | 2 | No | Alcohol And Tobacco Control |
| [70] | Baluku2021 | Uganda | 1 | quasi-experimental study | 2 | No | Incentives |
| [24] | Becx2001 | Indonesia | 1 | non-RCT | 2 | No | Community-Based Intervention |
| [98] | Burzynski2022 | United States | 2 | RCT | 1 | No | Digital Health |
| [99] | Chaisson2001 | United States | 2 | RCT | 1 | No | Incentives, Education and Counselling |
| [100] | Chua2015 | Singapore | 2 | non-RCT | 2 | No | Incentives |
| [101] | Chuck2015 | United States | 2 | non-RCT | 2 | No | Digital Health |
| [28] | Datiko2009 | Southern Ethiopia | 1 | Cluster RCT | 1 | No | Community Based Interventions |
| [73] | Datiko2017 | Southern Ethiopia | 1 | pre and post intervention study | 2 | No | Community Based Interventions |
| [102] | Demissie2003 | Ethiopia | 1 | non-RCT | 2 | No | Community-Based Care |
| [74] | Diaw2018 | Senegal | 1 | pre and post intervention study | 2 | No | Education And Counseling, New Testing Tools/Strategies |
| [29] | Dudley2003 | South Africa | 1 | non-RCT | 2 | No | Community-Based Intervention |
| [103] | Fang2017 | China | 1 | Cluster RCT | 1 | No | Digital Health |
| [75] | Farooqi2017 | Pakistan | 1 | RCT | 1 | No | Digital Health |
| [76] | Fatima2022 | Pakistan | 1 | RCT | 1 | No | Community-Based Interventions |
| [104] | Guo2020 | China | 1 | RCT | 1 | No | Reminders And Tracers |
| [105] | Hermans2017 | Uganda | 1 | pre and post intervention study | 2 | Yes | Digital Health |
| [106] | Hsieh2008 | China (Taiwan) | 1 | RCT | 1 | No | Case Management |
| [107] | Jahnavi G2010 | India | 1 | RCT | 1 | No | Incentives |
| [77] | Janmeja2005 | India | 1 | RCT | 1 | No | Psychotherapy |
| [78] | John2015 | Nigeria | 1 | pre and post intervention study | 2 | No | Active Case Finding |
| [36] | Kaswaswa2022 | Malawi | 1 | Cluster RCT | 1 | No | Active Case Finding |
| [79] | Khan2011 | Pakistan | 1 | pre and post intervention study | 2 | No | Improve Facility TB Care |
| [80] | Khortwong2013 | Thailand | 1 | pre and post intervention study | 2 | No | Education And Counselling |
| [108] | Kufa2018 | South Africa | 1 | Cluster RCT | 1 | Yes | Staff Training |
| [81] | Kunawararak2011 | Thailand | 1 | RCT | 1 | No | Digital Health |
| [83] | Lewin2005 | South Africa | 1 | RCT | 1 | No | Staff Training |
| [109] | Liefooghe1999 | Pakistan | 1 | RCT | 1 | No | Education And Counselling |
| [85] | Lutge2013 | South Africa | 1 | Cluster RCT | 1 | No | Incentives |
| [110] | Martins2009 | Timor-Leste | 1 | RCT | 1 | No | Incentives |
| [111] | Miller2010 | Brazil | 1 | Cluster RCT | 1 | No | Active Case Finding |
| [87] | Miti2003 | Zambia | 1 | non-RCT | 2 | Yes | Home-Based Care |
| [112] | Mohammed2016 | Pakistan | 1 | RCT | 1 | No | Digital Health |
| [113] | Morisky2001 | United States | 2 | RCT | 1 | No | Incentives, Psychotherapy, Mixed Intervention |
| [114] | Moulding2002 | Haiti | 1 | RCT | 1 | No | Reminders And Tracers |
| [115] | Nyamathi 2006 | United States | 2 | RCT | 1 | No | Education And Counseling, Incentives |
| [90] | Peltzer2013 | South Africa | 1 | Cluster RCT | 1 | No | Alcohol And Tobacco Control |
| [116] | Plokhykh2021 | Ukraine | 1 | Quasi-experimental Study | 2 | No | Alcohol And Tobacco Control, Education and Counselling |
| [91] | Puchalski2015 | Malawi | 1 | Cluster RCT | 1 | No | Staff Training, Mixed Interventions |
| [117] | Rajasekaran1993 | India | 1 | RCT | 1 | No | Reminders And Tracers |
| [118] | Rocha2011 | Peru | 1 | pre and post intervention study | 2 | No | Household Visits |
| [112] | Mohammed2016 | Pakistan | 1 | RCT | 1 | No | Digital Health |
| [92] | Taneja2017 | India | 1 | quasi-experimental study | 2 | No | Home-Based Care |
| [93] | Thekkur 2019 | India | 1 | Mixed methods | 2 | Yes | Digital Health, And Integrated with HIV Care |
| [94] | Thiam, S.2007 | Senegal | 1 | Cluster RCT | 1 | No | Mixed Interventions |
| [50] | Timire2018 | Zimbabwe, Southern African | 1 | pre and post intervention study | 2 | No | Improved Facility TB Care |
| [119] | Turnbull2021 | England | 2 | pre and post intervention study | 2 | No | Improved Facility TB Care |
| [120] | Wei2012 | China | 1 | Interventional study | 2 | No | Incentives |
| [65] | Wingfield2017 | Peru | 1 | RCT | 1 | No | Incentives |
| [97] | Wongduan2015 | Thailand | 1 | RCT | 1 | No | Mixed Interventions |
| [121] | Yao2008 | China | 1 | Cluster RCT | 1 | No | Incentives |
| [53] | Yassin2013 | Ethiopia | 1 | pre and post intervention study | 2 | No | Community-Based Intervention |
| [122] | Zou2013 | China | 1 | pre and post intervention study | 2 | No | Incentives |
|  | **Treatment Success** |  |  |  |  |  |  |
| [123] | Acosta2022 | Peru | 1 | RCT | 1 | No | Reminders And Tracers |
| [55] | Al. Sahafi2021 | Saudi Arabia | 1 | randomized control trial | 1 | No | Community-Based Interventions |
| [68] | Al-Sayali2021 | Saudi Arabia | 1 | RCT | 1 | No | Community-Based Interventions |
| [69] | Awaisu2011 | Malaysia | 1 | non-RCT | 2 | No | Alcohol And Tobacco Control |
| [24] | Becx2001 | Indonesia | 1 | non-RCT | 2 | No | Community-Based Intervention |
| [124] | Cattamanchi2021 | Uganda | 1 | a stepped-wedge cluster randomized trial | 1 | No | Digital Health |
| [28] | Datiko2009 | Southern Ethiopia | 1 | Cluster RCT | 1 | No | Community Based Interventions |
| [73] | Datiko2017 | Southern Ethiopia | 1 | pre and post intervention study | 2 | No | Community Based Interventions |
| [74] | Diaw2018 | Senegal | 1 | pre and post intervention study | 2 | No | Education And Counseling, New Testing Tools/Strategies Methods |
| [29] | Dudley2003 | South Africa | 1 | non-RCT | 2 | No | Community-Based Intervention |
| [75] | Farooqi2017 | Pakistan | 1 | RCT | 1 | No | Digital Health |
| [76] | Fatima2022 | Pakistan | 1 | RCT | 1 | No | Community-Based Interventions |
| [125] | Gashu2021 | Ethiopia | 1 | RCT | 1 | No | Reminders And Tracers |
| [106] | Hsieh2008 | China (Taiwan) | 1 | RCT | 1 | No | Case Management |
| [126] | Iribarren2013 | Argentina | 1 | pilot RCT | 1 | No | Digital Health |
| [77] | Janmeja2005 | India | 1 | RCT | 1 | No | Psychotherapy |
| [78] | John2015 | Nigeria | 1 | pre and post intervention study | 2 | No | Active Case Finding |
| [79] | Khan2011 | Pakistan | 1 | pre and post intervention study | 2 | No | Improve Facility TB Care |
| [80] | Khortwong2013 | Thailand | 1 | pre and post intervention study | 2 | No | Education And Counselling |
| [81] | Kunawararak2011 | Thailand | 1 | RCT | 1 | No | Digital Health |
| [127] | Lee2013 | Bangladesh | 1 | pre and post intervention study | 2 | No | Staff Training |
| [83] | Lewin2005 | South Africa | 1 | RCT | 1 | No | Staff Training |
| [84] | Louwagie2022 | South Africa | 1 | RCT | 1 | Yes | Alcohol And Tobacco Control |
| [128] | Lu2013 | China | 1 | pre and post intervention study | 2 | No | Incentives |
| [85] | Lutge2013 | South Africa | 1 | Cluster RCT | 1 | No | Incentives |
| [87] | Miti2003 | Zambia | 1 | non-RCT | 2 | Yes | Home-Based Care |
| [112] | Mohammed2016 | Pakistan | 1 | RCT | 1 | No | Digital Health |
| [60] | Mohan2003 | Iraq | 1 | RCT | 1 | No | Home-Based Care |
| [129] | Parwati2021 | Indonesia | 1 | RCT | 1 | No | Education And Counselling |
| [90] | Peltzer2013 | South Africa | 1 | Cluster RCT | 1 | No | Alcohol And Tobacco Control |
| [116] | Plokhykh2021 | Ukraine | 1 | Quasi-experimental Study | 2 | No | Alcohol And Tobacco Control, Education and counseling |
| [91] | Puchalski2015 | Malawi | 1 | Cluster RCT | 1 | No | Staff Training, Mixed interventions |
| [130] | Ravenscroft2020 | Moldova | 1 | RCT | 1 | No | Digital Health |
| [131] | Shargi2006 | Ethiopia | 1 | Cluster RCT | 1 | No | Community-Based Intervention |
| [132] | Shin2013 | Russian Federation | 2 | RCT | 1 | No | Alcohol And Tobacco Control |
| [133] | Soares2013 | Brazil. | 1 | before and after intervention study | 2 | No | Community-Based Intervention |
| [134] | Sudarsanam2011 | India | 1 | RCT | 1 | No | Incentives |
| [92] | Taneja2017 | India | 1 | quasi-experimental study | 2 | No | Home-Based Care |
| [93] | Thekkur,2019 | India | 1 | Mixed methods | 2 | Yes | Digital Health, And Integrated with HIV Care |
| [94] | Thiam, S.2007 | Senegal | 1 | Cluster RCT | 1 | No | Mixed Interventions |
| [135] | Thomas, B.2017 | India | 1 | Cluster RCT | 1 | No | Alcohol And Tobacco Control |
| [50] | Timire2018 | Zimbabwe, Southern African | 1 | pre and post intervention study | 2 | No | Improved Facility TB Care |
| [136] | Ukwaja, K N2017 | Nigeria | 1 | prospective non-randomized intervention | 2 | No | Incentives |
| [65] | Wingfield2017 | Peru | 1 | RCT | 1 | No | Incentives |
| [97] | Wongduan2015 | Thailand | 1 | RCT | 1 | No | Mixed Interventions |
| [53] | Yassin2013 | Ethiopia | 1 | pre and post intervention study | 2 | No | Community-Based Intervention |
|  | Note: Incentives = monetary and non-monetary incentives, mixed interventions = strategies made up more than 2 intervention activities. Details in of all interventions can be found in the primary article referenced in column 1. | | | | | | |

# Table D in S1 File Showing Results of Meta-regression Analysis

| **Outcome** | **Intervention** | **Factors** | **RR/OR (95% CI)** | ***P*-value** |
| --- | --- | --- | --- | --- |
| TB testing | Active case finding | Year of Publication | 0.89 (0.78, 1.02) | 0.1 |
|  |  | Study design | 1.09 (0.44, 2.69) | 0.85 |
|  |  | HIV integration | 0.38 (0.13, 1.10) | 0.075 |
|  | Education and counseling | Year of Publication | 0.87 (0.48, 1.58) | 0.64 |
|  |  | Study design | 0.10 (0.00, 40.59) | 0.46 |
|  |  | Region | 0.12 (0.00, 750.70) | 0.64 |
|  |  | HIV integration | 3.33 (0.01, 775.42) | 0.66 |
| TB diagnosis | Multi-sector collaboration | Year of Publication | 1.13 (0.73, 1.76) | 0.58 |
|  |  | Study design | 3.02 (0.02, 440.85) | 0.66 |
|  | Active case finding | Year of Publication | 1.02 (0.97, 1.06) | 0.49 |
|  |  | Study design | 0.97 (0.51, 1.83) | 0.92 |
|  |  | HIV integration | 0.95 (0.39, 2.31) | 0.91 |
|  | Education and counseling | Year of Publication | 1.00 (0.94, 1.06) | 0.87 |
|  |  | Study design | 0.84 (0.39, 1.78) | 0.64 |
|  |  | HIV integration | 0.99 (0.54, 1.80) | 0.96 |
|  | Improve diagnose technology and efficiency | Year of Publication | 0.96 (0.87, 1.07) | 0.48 |
|  |  | Study design | 0.64 (0.52, 0.79) | 2.20E-05 |
|  |  | Region | 1.18 (0.49, 2.83) | 0.71 |
|  |  | HIV integration | 1.25 (0.92, 1.70) | 0.15 |
|  | Improve facility TB care | Year of Publication | 1.09 (0.96, 1.24) | 0.2 |
|  |  | Study design | 1.10 (0.14, 8.46) | 0.92 |
|  |  | HIV integration | 1.77 (0.28, 11.23) | 0.54 |
| Linkage to care | Active case finding | Year of Publication | 1.03 (0.95, 1.11) | 0.46 |
|  |  | Study design | 1.22 (0.69, 2.15) | 0.5 |
|  | Mixed interventions | Year of Publication | 0.88 (0.68, 1.14) | 0.35 |
|  |  | **Study design** | **9.44 (1.04, 85.59)** | **0.046** |
|  |  | HIV integration | 0.41 (0.05, 3.24) | 0.4 |
|  | Digital interventions | Year of Publication | 1.34 (0.84, 2.14) | 0.22 |
|  |  | HIV integration | 0.48 (0.15, 1.52) | 0.21 |
|  | Patient navigator and referral system | Year of Publication | 0.66 (0.11, 4.10) | 0.65 |
|  |  | Study design | 0.22 (0.00, 1321.79) | 0.73 |
| Treatment success | Incentives | **Year of Publication** | **1.03 (1.00, 1.05)** | **0.039** |
|  |  | Study design | 0.97 (0.91, 1.04) | 0.42 |
|  | Alcohol and tobacco control | **Year of Publication** | **0.88 (0.81, 0.96)** | **0.0026** |
|  |  | Study design | 0.40 (0.25, 0.65) | 2.00E-04 |
|  |  | Region | 0.42 (0.28, 0.64) | 5.00E-05 |
|  |  | HIV integration | 0.83 (0.43, 1.60) | 0.57 |
|  | Community-based intervention | Year of Publication | 1.00 (0.98, 1.02) | 0.93 |
|  |  | Study design | 1.13 (0.90, 1.42) | 0.29 |
|  | Digital interventions | Year of Publication | 0.99 (0.97, 1.02) | 0.54 |
|  |  | Study design | 0.87 (0.72, 1.05) | 0.14 |
|  | Education and counseling | Year of Publication | 0.97 (0.95, 0.99) | 1.40E-02 |
|  |  | Study design | 1.00 (0.75, 1.33) | 1 |
| Cured | Incentives | Year of Publication | 1.05 (0.94, 1.18) | 0.36 |
|  |  | Study design | 1.10 (0.65, 1.86) | 0.72 |
|  | Community-based intervention | Year of Publication | 1.02 (1.00, 1.05) | 0.075 |
|  |  | **Study design** | **1.46 (1.00, 2.14)** | **0.051** |
|  | Digital interventions | **Year of Publication** | **0.93 (0.89, 0.97)** | **0.00033** |
|  |  | **Study design** | **1.67 (1.11, 2.51)** | **0.013** |
|  |  | HIV integration | 3.81 (2.27, 6.39) | 3.80E-07 |
|  | Education and counseling | Year of Publication | 0.96 (0.94, 0.98) | 5.50E-05 |
|  |  | Study design | 1.09 (0.72, 1.66) | 0.67 |
| Treatment completed | Incentives | Year of Publication | 1.00 (0.98, 1.02) | 0.85 |
|  |  | Study design | 1.05 (0.88, 1.25) | 0.56 |
|  |  | Region | 0.92 (0.79, 1.08) | 0.32 |
|  | Community-based intervention | **Year of Publication** | **0.96 (0.93, 0.99)** | **0.014** |
|  |  | Study design | 0.81 (0.48, 1.38) | 0.44 |
|  | Mixed interventions | Year of Publication | 1.02 (0.94, 1.11) | 0.6 |
|  |  | Region | 1.69 (0.72, 3.95) | 0.23 |
|  | Digital interventions | Year of Publication | 1.15 (1.08, 1.22) | 8.00E-06 |
|  |  | Study design | 2.52 (1.64, 3.89) | 2.80E-05 |
|  |  | Region | 0.46 (0.33, 0.64) | 4.80E-06 |
|  |  | HIV integration | 2.94 (1.73, 5.01) | 7.20E-05 |
|  | Education and counseling | Year of Publication | 1.09 (1.05, 1.13) | 1.40E-05 |
|  |  | **Study design** | **0.25 (0.12, 0.52)** | **0.00022** |
|  |  | **Region** | **0.76 (0.63, 0.92)** | **0.0041** |

# Table E in S1 File Showing Outcome of Risk of Bias Assessment for 84 RCTs Studies Using the Cochrane Risk of Bias Assessment Tool

| **ID** | Selection bias | Performance bias | Detection bias | Attrition bias | Reporting bias | Other bias |  |
| --- | --- | --- | --- | --- | --- | --- | --- |
|  | **Random sequence generation** | **Allocation concealment** | **Blinding (participants and personnel)** | **Blinding (outcome assessment)** | **Incomplete outcome data** | **Selective reporting** | **Other sources of bias** |
| Mohammed 2016 | Low | Unclear | Low | Low | Low | Unclear | Low |
| Acosta2022 | Low | Low | High | Low | Low | Low | Unclear |
| Adane2019 | Unclear | Unclear | Unclear | Unclear | High | Unclear | Unclear |
| Al. Sahafi2021 | High | Low | Unclear | Unclear | Low | High | High |
| Aldridge2015 | Low | Low | High | Low | Low | Unclear | High |
| Alisjahbana2005 | Low | Unclear | High | High | Low | Low | High |
| Al-Sayali2021 | Unclear | Unclear | High | Unclear | Unclear | Unclear | Unclear |
| Bassett2016 | Unclear | High | High | Low | Low | Unclear | Unclear |
| Bediang2018 | High | Unclear | High | High | High | Unclear | Unclear |
| Bello2017 | Low | Low | Low | Low | Low | Unclear | Unclear |
| Burzynski2022 | Low | Unclear | High | Low | Low | Unclear | Unclear |
| Calligaro2017 | High | High | High | High | Low | Unclear | Unclear |
| Cattamanchi2021 | Unclear | High | Low | Low | Low | High | High |
| Chaisson2001 | Unclear | Unclear | High | Unclear | Low | Unclear | High |
| Churchyard2011 | Unclear | Unclear | High | Unclear | High | Unclear | High |
| Corbett2010 | Low | Unclear | Low | Unclear | High | High | High |
| Datiko2009 | High | Low | Low | Low | Low | Unclear | Unclear |
| Durovni2013 | Unclear | Unclear | High | High | High | Unclear | High |
| Durovni2014 | High | Low | High | High | High | Low | Unclear |
| Eom2022 | Unclear | Unclear | High | Low | High | Unclear | Unclear |
| Fairall 2005 | Low | Low | High | Low | Unclear | Unclear | Unclear |
| Fang2017 | Low | Unclear | High | Low | High | Unclear | Unclear |
| Farooqi2017 | Low | Low | High | High | Low | Unclear | High |
| Fatima2022 | Unclear | Unclear | High | High | High | Unclear | Unclear |
| Gengiah2021 | Unclear | Unclear | Low | Low | Low | Unclear | Unclear |
| Griffiths2007 | High | Low | High | Low | Low | High | High |
| Guo2020 | Unclear | Unclear | High | High | Low | High | High |
| Hsieh2008 | High | High | High | High | Low | Unclear | Unclear |
| Iribarren2013 | Unclear | Low | High | Low | Low | Unclear | High |
| Jahnavi G2010 | Low | Low | High | Low | Low | High | High |
| Janmeja2005 | Unclear | Unclear | High | Unclear | Low | Unclear | High |
| Jenum2018 | Low | Low | Low | Low | Low | High | High |
| Kaswaswa2022 | Unclear | Unclear | High | Low | High | Unclear | Unclear |
| Khan2007 | Low | Unclear | High | Unclear | High | Unclear | Unclear |
| Khan2016 One | Unclear | Unclear | High | Unclear | High | High | High |
| Khatana2019 | Unclear | Unclear | High | High | Low | Unclear | Unclear |
| Kufa2018 | Unclear | Unclear | High | Unclear | High | Unclear | Unclear |
| Kunawararak2011 | Unclear | Unclear | Low | Unclear | High | High | High |
| Lee2011 | Low | Unclear | High | Low | Low | High | High |
| Lewin2005 | High | Unclear | High | High | Low | Low | Low |
| Liefooghe1999 | Unclear | Unclear | High | High | High | Unclear | Unclear |
| Louwagie2022 | High | Low | High | High | Unclear | Unclear | High |
| Lutge2013 | High | Unclear | High | Low | Low | Low | Unclear |
| MacPherson2021 | Low | Unclear | High | Unclear | Low | High | High |
| Majella2021 | Unclear | Low | High | High | Low | Unclear | Unclear |
| Manyazewal2022 | Low | Unclear | High | Unclear | High | Unclear | Unclear |
| Martins2009 | Unclear | Unclear | High | High | High | Unclear | Unclear |
| Martinson2022 | Low | Unclear | High | Unclear | High | High | High |
| Mhalu2015 | Unclear | Unclear | Unclear | Unclear | Low | High | High |
| Miller2010 | Unclear | Unclear | High | High | Low | Unclear | Unclear |
| Mohammed2016 | Unclear | Unclear | High | Unclear | Low | Unclear | Unclear |
| Mohan2003 | Unclear | Unclear | High | Unclear | High | Unclear | Unclear |
| Morisky2001 | Unclear | Unclear | High | Unclear | High | Unclear | Unclear |
| Moulding2002 | Low | Low | High | Low | Unclear | Unclear | Unclear |
| Mukoka2022 | Low | Low | High | Low | Low | Unclear | High |
| Müller2019 | Unclear | Unclear | Low | Low | Low | High | High |
| Mwansa-Kambafwile2022 | Unclear | Unclear | High | Unclear | High | Unclear | High |
| Niazi2003 | High | Unclear | Unclear | Unclear | High | Unclear | Unclear |
| Nyamathi 2006 | Unclear | Unclear | Unclear | Unclear | Low | Unclear | High |
| Parwati2021 | Low | Low | High | Unclear | Low | High | High |
| Peltzer2013 | Unclear | Low | Low | Low | Low | Low | Low |
| Puchalski2015 | Unclear | Low | Low | Low | Low | Low | Low |
| Rajasekaran1993 | Low | Low | Low | High | Unclear | Low | Low |
| Ravenscroft2020 | Low | Low | Low | Low | Low | Low | Low |
| Rudolf2021 | Unclear | Unclear | Low | Unclear | Unclear | Unclear | High |
| Rüütel2011 | Unclear | Low | Low | Low | Unclear | Unclear | Low |
| Sequeira-Aymar2021 | High | High | Low | Low | Low | Low | Low |
| Shah, Lena2020 | Low | Low | Low | Low | Low | Low | Low |
| Shargi2006 | Low | Low | Low | Low | Low | Low | Low |
| Shin2013 | Low | Low | Unclear | Low | Unclear | Low | Low |
| Sudarsanam2011 | High | Low | Low | Unclear | Low | Low | Low |
| Thiam, S.2007 | Low | Low | Low | Low | Low | Low | Low |
| Thomas, B.2017 | Low | Low | Low | Low | Low | Low | Low |
| Uwimana2012 | High | Low | Low | Low | Unclear | Low | Low |
| Venkatapraveen2012 | Low | Low | Low | Unclear | Unclear | Unclear | Low |
| Wagstaff, Adam2019 | Low | Low | Low | Low | Low | Low | Low |
| White1998 | Low | Low | Low | Low | Low | Low | Low |
| Wingfield2017 | Low | Low | High | Low | Unclear | Unclear | Low |
| Wongduan2015 | Low | Low | Low | Low | Unclear | Unclear | Low |
| Yao2008 | Unclear | Low | Low | Unclear | Unclear | Low | Low |
| Yassi2016 | Low | Low | Low | Low | Low | Low | Low |
| Yellappa2018 | Low | Low | High | Low | Low | Low | High |
| Zwarenstein2011 | Unclear | Low | Low | Low | High | Low | Low |

# Table F in S1 File Showing Quality Assessment of Included Studies Using the EPHPP Quality Assessment Tool.

| ID | Selection Bias | Study Design | Confounders | Blinding | Data Collection | Withdraw | Overall |
| --- | --- | --- | --- | --- | --- | --- | --- |
| Ali2020 | 2 | 1 | 3 | 3 | 1 | 1 | Weak |
| Awaisu2011 | 2 | 1 | 1 | 3 | 1 | 2 | Moderate |
| Bai2008 | 1 | 1 | 3 | 3 | 1 | 1 | Weak |
| Balakrishnan2021 | 1 | 1 | 2 | 1 | 1 | 1 | Strong |
| Baluku2021 | 1 | 1 | 1 | 2 | 1 | 1 | Strong |
| Becx2001 | 1 | 1 | 1 | 1 | 1 | 1 | Strong |
| Bjerrum2016 | 1 | 1 | 2 | 3 | 1 | 1 | Moderate |
| Broomhead2012 | 1 | 1 | 1 | 3 | 1 | 2 | Moderate |
| Chaisson1996 | 1 | 1 | 1 | 3 | 1 | 1 | Moderate |
| Chua2015 | 1 | 1 | 2 | 2 | 3 | 2 | Moderate |
| Chuck2015 | 2 | 1 | 1 | 3 | 1 | 2 | Moderate |
| Datiko2017 | 3 | 1 | 1 | 2 | 1 | 1 | Moderate |
| Davis2011 | 2 | 1 | 2 | 2 | 1 | 3 | Moderate |
| Demissie2003 | 2 | 1 | 2 | 2 | 1 | 2 | Moderate |
| Diaw2018 | 2 | 1 | 1 | 2 | 1 | 3 | Moderate |
| Dudley2003 | 1 | 1 | 1 | 2 | 1 | 2 | Strong |
| Ekwueme2014 | 1 | 1 | 1 | 2 | 1 | 3 | Moderate |
| Fatima2016 | 1 | 1 | 2 | 3 | 1 | 2 | Moderate |
| Fitzgerald 1999 | 2 | 1 | 1 | 2 | 1 | 3 | Moderate |
| Garg2020 | 3 | 2 | 1 | 3 | 1 | 3 | Weak |
| Geldenhuys2014 | 2 | 1 | 3 | 2 | 1 | 1 | Moderate |
| Harstad 2014 | 2 | 1 | 1 | 2 | 1 | 2 | Moderate |
| Hermans2017 | 1 | 1 | 1 | 3 | 1 | 2 | Moderate |
| John2015 | 2 | 1 | 2 | 3 | 1 | 3 | Weak |
| Khan2011 | 1 | 1 | 1 | 3 | 1 | 3 | Weak |
| Khan2016 Two | 2 | 1 | 2 | 2 | 1 | 3 | Weak |
| Khortwong2013 | 2 | 1 | 1 | 3 | 3 | 3 | Weak |
| Lambert2005 | 2 | 1 | 3 | 2 | 1 | 1 | Moderate |
| Law2021 | 3 | 1 | 1 | 3 | 1 | 2 | Weak |
| Lee2013 | 2 | 1 | 1 | 2 | 1 | 3 | Moderate |
| Lee2015 | 2 | 1 | 1 | 2 | 1 | 1 | Moderate |
| Lisboa2020 | 2 | 1 | 1 | 3 | 1 | 1 | Moderate |
| Lu2013 | 2 | 1 | 1 | 2 | 3 | 3 | Weak |
| Malotte1999 | 2 | 1 | 1 | 2 | 1 | 3 | Moderate |
| Miti2003 | 2 | 1 | 3 | 2 | 2 | 2 | Moderate |
| Parija2014 | 2 | 1 | 3 | 2 | 3 | 1 | Weak |
| Perlman2003 | 1 | 1 | 1 | 2 | 1 | 3 | Moderate |
| Plokhykh2021 | 1 | 2 | 1 | 3 | 1 | 2 | Moderate |
| Qureshi2010 | 2 | 1 | 2 | 2 | 2 | 2 | Moderate |
| Rocha2011 | 1 | 1 | 1 | 2 | 2 | 1 | Strong |
| Sah2021 | 3 | 2 | 1 | 3 | 1 | 1 | Weak |
| Simwaka2012 | 1 | 1 | 2 | 2 | 1 | 3 | Moderate |
| Soares2013 | 3 | 1 | 2 | 2 | 1 | 3 | Weak |
| Taneja2017 | 2 | 1 | 2 | 2 | 3 | 2 | Moderate |
| Thekkur2019 | 1 | 1 | 3 | 1 | 1 | 3 | Moderate |
| Thu2019 | 1 | 2 | 3 | 3 | 1 | 1 | Weak |
| Timire2018 | 1 | 1 | 2 | 2 | 1 | 2 | Strong |
| Torrens2016 | 1 | 1 | 1 | 1 | 1 | 1 | Strong |
| Turnbull2021 | 2 | 2 | 3 | 3 | 1 | 1 | Weak |
| Ukwaja2017 | 3 | 1 | 1 | 2 | 2 | 1 | Weak |
| Uwimana2013 | 1 | 1 | 1 | 2 | 1 | 2 | Strong |
| Vo2020 | 1 | 1 | 1 | 3 | 1 | 1 | Moderate |
| Volkmann2016 | 1 | 1 | 1 | 2 | 2 | 2 | Strong |
| Wang 2009 | 3 | 1 | 3 | 2 | 1 | 2 | Weak |
| Wei2012 | 3 | 1 | 2 | 2 | 3 | 1 | Moderate |
| Wei2015 | 1 | 1 | 1 | 2 | 1 | 2 | Strong |
| Yassin2013 | 1 | 1 | 2 | 2 | 1 | 2 | Strong |
| Zaeh2013 | 1 | 1 | 1 | 2 | 3 | 2 | Moderate |
| Zhu2012 | 1 | 1 | 2 | 2 | 1 | 2 | Strong |
| Zou2013 | 1 | 1 | 2 | 2 | 3 | 2 | Moderate |
| ^Note: The EPHPP tool assessed each study in seven main domains (selection bias, study design, confounders, blinding, data collection, methods, and withdrawals and dropouts of patients) and rated each aspect as strong (strong), moderate (2), or weak quality (3). Studies graded as weak quality (3) in two or more domains as having an overall poor quality [137].^ | | | | | | | |

# Table G in S1 File Showing GRADE Outcomes

## Summary of GRADE Assessment of Quality of Evidence Certainty for each Outcome of interest for TB testing

| **Certainty assessment** | | | | | | | **№ of patients** | | **Effect** | | **Certainty** | **Importance** |
| --- | --- | --- | --- | --- | --- | --- | --- | --- | --- | --- | --- | --- |
| **№ of studies** | **Study design** | **Risk of bias** | **Inconsistency** | **Indirectness** | **Imprecision** | **Other considerations** | **Intervention** | **[Standard-of-care]** | **Relative (95% CI)** | **Absolute (95% CI)** |  |  |
| **Digital Interventions** | | | | | | | | | | | | |
| 2 | Randomized trials | not serious | not serious | not serious | not serious | none | 529/8859 (6.0%) | 291/8293 (3.5%) | **OR 1.97** (1.28 to 3.04) | **32 more per 1,000**  (from 9 more to 64 more) | ⨁⨁⨁⨁  High | CRITICAL |
| **Incentives** | | | | | | | | | | | | |
| 5 | Observational studies | not serious | Serious ^b,c^ | not serious | not serious | strong association | 1101/1430 (77.0%) | 654/1475 (44.3%) | **OR 1.74** (1.63 to 1.85) | **138 more per 1,000** (from 122 more to 152 more) | ⨁⨁◯◯  Low | IMPORTANT |
| Explanation: **C.I =**confidence interval; **OR=**odds ratio; b. high risk of selection bias; c. Variations in sample sizes and types of incentives | | | | | | | | | | | | |
| **Education and counselling** | | | | | | | | | | | | |
| 7 | 3RCT, 4 non-RCT | Serious ^a^ | Serious ^b^ | not serious | not serious | very strong association | 20920/36335 (57.6%) | 2763/35033 (7.9%) | **OR 8.82** (1.71 to 45.43) | **351 more per 1,000** (from 49 more to 717 more) | ⨁⨁⨁⨁  High |  |
| 3 | Randomized trials | serious ^c^ | serious ^d^ | not serious | not serious | strong association | 20290/35475 (57.2%) | 2432/33951 (7.2%) | **OR 12.81** (0.41 to 402.75) | **425 more per 1,000** (from 41 fewer to 897 more) | ⨁⨁⨁◯  Moderate |  |
| 4 | Observational studies | serious ^e^ | not serious | not serious | Serious ^f^ | none | 630/860 (73.3%) | 331/1082 (30.6%) | **OR 6.63** (1.11 to 39.55) | **439 more per 1,000** (from 23 more to 640 more) | ⨁◯◯◯  Very low |  |
| Explanations: c. Two studies had high risks of selection, performance, and attrition bias, one study had unclear risks of bias; d. variations in counseling and education strategies may have affected intervention outcomes; e. Two studies had moderate risks of selection and study design bias; f. Variations in study sample sizes (some studies had small sample sizes) | | | | | | | | | | | | |
| **Active Case Finding** | | | | | | | | | | | | |
| 7 | 3RCT, 4 non-RCTs | Serious ^a^ | Serious ^b^ | not serious | not serious | none | 5047/283046 (1.8%) | 3651/185264 (2.0%) | **OR 1.68** (0.68 to 4.13) | **13 more per 1,000** (from 6 fewer to 57 more) | ⨁⨁◯◯ Low | IMPORTANT |
| 3 | Randomized trials | serious ^c^ | not serious | not serious | not serious | none | 2768/19412 (14.3%) | 2141/22825 (9.4%) | **OR 1.56** (0.35 to 6.95) | **45 more per 1,000** (from 59 fewer to 325 more) | ⨁⨁⨁◯  Moderate |  |
| 4 | Observational studies | not serious | Serious ^d^ | not serious | serious ^e^ | all plausible residual confounding would reduce the demonstrated effect | 2279/263634 (0.9%) | 1510/162439 (0.9%) | **OR 1.81** (0.48 to 6.75) | **7 more per 1,000** (from 5 fewer to 50 more) | ⨁◯◯◯  Very low |  |
| **Explanations** c. No blinding in one study; d. Inconsistent results (some studies have inconsistent ORs); e. Variations in sample size/type of intervention implementation approach | | | | | | | | | | | | |
| **Multi-Sector Collaborations** | | | | | | | | | | | | |
| 2 | 1RCT, 1 Observational | not serious | not serious | not serious | not serious | none | 666/7086 (9.4%) | 0.0% | **OR 4.14** (3.42 to 5.01) | **0 fewer per 1,000** (from 0 fewer to 0 fewer) | ⨁⨁◯◯ Low | CRITICAL |
| 1 | Randomized trials | not serious | not serious | not serious | not serious | none | 557/6963 (8.0%) | 164/8200 (2.0%) | **OR 4.00** (3.37 to 4.75) | **55 more per 1,000** (from 44 more to 68 more) | ⨁⨁⨁⨁  High |  |
| 1 | Observational studies | not serious | Serious ^a^ | not serious | not serious | none | 109/123 (88.6%) | 97/134 (72.4%) | **OR 1.22** (1.08 to 1.38) | **38 more per 1,000** (from 15 more to 60 more) | ⨁◯◯◯  Very low |  |
| **Explanations:** a. The sample size in the non-RCT was smaller | | | | | | | | | | | | |
| **Staff training** | | | | | | | | | | | | |
| 1 | Randomized trials | not serious | not serious | not serious | not serious | none | 2637/200714 (1.3%) | 2658/209564 (1.3%) | **OR 1.04** (0.98 to 1.09) | **1 more per 1,000** (from 0 fewer to 1 more) | ⨁⨁⨁⨁ High | IMPORTANT |

| Summary of GRADE Assessment of Quality of Evidence Certainty for each Outcome of interest for TB Diagnosis | | | | | | | | | | | | |
| --- | --- | --- | --- | --- | --- | --- | --- | --- | --- | --- | --- | --- |
| **Certainty assessment** | | | | | | | **№ of patients** | | **Effect** | | **Certainty** | **Importance** |
| **№ of studies** | **Study design** | **Risk of bias** | **Inconsistency** | **Indirectness** | **Imprecision** | **Other considerations** | **[Intervention]** | **[Standard-of-care]** | **Relative (95% CI)** | **Absolute (95% CI)** |  |  |
| **Counseling and Education** | | | | | | | | | | | | |
| 9 | 7RCTs, 2 non-RCTs | not serious ^a^ | not serious | not serious | not serious | none | 11411/2430214 (0.5%) | 5176/782446 (0.7%) | **OR 1.44** (1.08 to 1.92) | **3 more per 1,000** (from 1 more to 6 more) | ⨁⨁⨁⨁ High | CRITICAL |
| 7 | Randomized trials | not serious | not serious | not serious | not serious | strong association | 11312/2429472 (0.5%) | 5096/781775 (0.7%) | **OR 1.58** (1.09 to 2.29) | **4 more per 1,000** (from 1 more to 8 more) | ⨁⨁⨁⨁ High | CRITICAL |
| 2 | observational studies | not serious ^b^ | not serious | not serious | not serious | none | 99/742 (13.3%) | 80/671 (11.9%) | **OR 1.13** (0.86 to 1.49) | **13 more per 1,000** (from 15 fewer to 49 more) | ⨁⨁◯◯ Low | IMPORTANT |
| Explanation: b. Randomization and blinding were impossible due to study design | | | | | | | | | | | | |
| **Active Case Finding** | | | | | | | | | | | | |
| 12 | 6RCTs, 6n non-RCTs | Serious ^a^ | Serious ^b^ | not serious | not serious | none | 614622/7005056 (8.8%) | 9886/9273133 (0.1%) | **OR 1.24** (0.96 to 1.60) | **0 fewer per 1,000** (from 0 fewer to 1 more) | ⨁⨁◯◯ Low | IMPORTANT |
| 6 | Randomized trials | Serious ^c^ | Serious ^b^ | not serious | not serious | none | 1044/188508 (0.6%) | 800/127858 (0.6%) | **OR 1.44** (1.21 to 1.70) | **3 more per 1,000** (from 1 more to 4 more) | ⨁⨁◯◯ Low | IMPORTANT |
| 6 | observational studies | not serious | not serious ^d^ | not serious | not serious | none | 13578/6816548 (0.2%) | 9086/9145275 (0.1%) | **OR 1.16** (0.67 to 1.80) | **0 fewer per 1,000** (from 0 fewer to 1 more) | ⨁⨁◯◯ Low | CRITICAL |
| Explanations: c= Risk of bias unclear; d= other risks of bias (confounders); e=selection bias, confounders and two studies of weak quality; f=Variations in sample sizes | | | | | | | | | | | | |
| **Multi-sector Collaborations** | | | | | | | | | | | | |
| 5 | 1RCT, 4 non-RCTs | not serious ^a^ | very serious ^b^ | not serious | not serious | none | 3018/68262 (4.4%) | 1332/47428 (2.8%) | **RR 11.50** (1.65 to 80.37) | **295 more per 1,000** (from 18 more to 1,000 more) | ⨁⨁◯◯ Low | CRITICAL |
| 1 | Randomized trials | Serious ^c^ | not serious | not serious | Serious ^d^ | none | 98/700 (14.0%) | 42/700 (6.0%) | **OR 2.33** (1.65 to 3.30) | **69 more per 1,000** (from 35 more to 114 more) | ⨁⨁◯◯ Low | CRITICAL |
| 4 | observational studies | Serious ^e^ | Serious ^b^ | not serious | Serious ^f^ | none | 2920/46728 (6.2%) | 1290/67562 (1.9%) | **OR 10.70** (1.42 to 80.49) | **153 more per 1,000** (from 8 more to 591 more) | ⨁◯◯◯ Very low | IMPORTANT |
| Explanation: b. Studies have inconsistent ORs; c. Some studies had a moderate risk of selection bias, were not blinded, and had a moderate risk of confounders, d. All studies were of high quality except for one | | | | | | | | | | | | |
| **Improved diagnosis technology and efficiency** | | | | | | | | | | | | |
| 8 | 7RCTs, 1 non-RCT | not serious ^a^ | not serious | not serious | not serious | none | 30429/122737 (24.8%) | 31652/113411 (27.9%) | **OR 1.45** (0.91 to 2.33) | **80 more per 1,000** (from 19 fewer to 195 more) | ⨁⨁⨁⨁ High | IMPORTANT |
| 7 | Randomized trials | not serious | not serious | not serious | not serious | none | 2270/14396 (15.8%) | 1586/13027 (12.2%) | **OR 1.68** (1.16 to 2.41) | **67 more per 1,000** (from 17 more to 129 more) | ⨁⨁⨁⨁ High | IMPORTANT |
| 1 | observational studies | not serious ^b^ | Serious ^c^ | not serious | not serious | strong association | 28159/108341 (26.0%) | 30066/100384 (30.0%) | **OR 0.87** (0.86 to 0.88) | **28 fewer per 1,000** (from 31 fewer to 26 fewer) | ⨁⨁◯◯ Low |  |
| Explanations: b. Blinding and randomization not feasible due to study design; c. Confounders | | | | | | | | | | | | |
| **Improved facility-based TB Care** | | | | | | | | | | | | |
| 9 | 2RCTs, 7 non-RCTs | very serious ^a^ | not serious | not serious | not serious | strong association | 51739/209776 (24.7%) | 21546/262855 (8.2%) | **OR 2.69** (1.16 to 6.22) | **112 more per 1,000** (from 12 more to 275 more) | ⨁⨁⨁◯ Moderate | CRITICAL |
| 2 | Randomized trials | Serious ^b^ | Serious ^c^ | not serious | not serious | strong association | 630/6063 (10.4%) | 382/4588 (8.3%) | **OR 1.83** (0.76 to 4.40) | **59 more per 1,000** (from 19 fewer to 202 more) | ⨁⨁⨁◯ Moderate |  |
| 7 | observational studies | Serious ^d^ | not serious | not serious | not serious ^e^ | strong association all plausible residual confounding would reduce the demonstrated effect | 51109/203713 (25.1%) | 21164/258267 (8.2%) | **OR 2.97** (1.01 to 8.73) | **128 more per 1,000** (from 1 more to 356 more) | ⨁⨁⨁◯ Moderate |  |
| Explanations: b= Method of randomization sequence generation unclear; c= Small sample size; d= 3 studies were of weak quality; e= Some studies have a small sample size | | | | | | | | | | | | |

| Summary of GRADE Assessment of Quality of Evidence Certainty for each Outcome of interest for linkage-to-care | | | | | | | | | | | | |
| --- | --- | --- | --- | --- | --- | --- | --- | --- | --- | --- | --- | --- |
| **Certainty assessment** | | | | | | | **№ of patients** | | **Effect** | | **Certainty** | **Importance** |
| **№ of studies** | **Study design** | **Risk of bias** | **Inconsistency** | **Indirectness** | **Imprecision** | **Other considerations** | **[Intervention]** | **[Standard-of-care]** | **Relative (95% CI)** | **Absolute (95% CI)** |  |  |
| **Patient Navigation & Referral Systems** | | | | | | | | | | | | |
| 4 | Randomized trials | not serious | not serious | not serious | Serious ^a^ | none | 4504/26214 (17.2%) | 6923/33344 (20.8%) | **OR 1.99** (1.23 to 3.24) | **206 more per 1,000** (from 48 more to 465 more) | ⨁⨁⨁◯ Moderate |  |
| Explanations: **CI:** confidence interval; O**R:** Odds ratio; a. One study had very large sample size | | | | | | | | | | | | |
| **Multi-sector Collaborations** | | | | | | | | | | | | |
| 1 | Randomized trials | not serious | not serious | not serious | Serious ^b^ | none | 55/94 (58.5%) | 40/95 (42.1%) | **OR 1.94** (1.09 to 3.46) | **164 more per 1,000** (from 21 more to 295 more) | ⨁⨁⨁◯ Moderate | CRITICAL |
| 2 | Observational studies | not serious | not serious | not serious | not serious | strong association | 786/1061 (74.1%) | 357/665 (53.7%) | **OR 3.66** (2.90 to 4.62) | **272 more per 1,000** (from 234 more to 306 more) | ⨁⨁⨁◯ Moderate | CRITICAL |
| Explanation: b. small sample size | | | | | | | | | | | | |
| **Community-based Interventions** | | | | | | | | | | | | |
| 1 | Randomized trials | not serious | not serious | not serious | Serious ^b^ | strong association | 97/100 (97.0%) | 76/100 (76.0%) | **OR 10.21** (2.96 to 35.19) | **210 more per 1,000** (from 144 more to 231 more) | ⨁⨁⨁⨁ High | IMPORTANT |
| 2 | Observational studies | not serious | not serious | not serious | Serious ^c^ | very strong association | 592/2223 (26.6%) | 15/1685 (0.9%) | **OR 37.08** (22.09 to 62.24) | **241 more per 1,000** (from 157 more to 350 more) | ⨁⨁⨁◯ Moderate |  |
| Explanations: b. small sample size; c. One study has a small sample size | | | | | | | | | | | | |
| **Active Case Finding** | | | | | | | | | | | | |
| 1 | Randomized trials | serious ^b^ | not serious | not serious | not serious | none | 2215/2215 (100.0%) | 2167/2167 (100.0%) | **OR 1.02** (0.02 to 51.54) | **0 fewer per 1,000** (from 0 fewer to 0 fewer) | ⨁⨁⨁◯ Moderate |  |
| 2 | Observational studies | not serious | not serious ^c^ | not serious | serious ^d^ | none | 24852/26953 (92.2%) | 11777/12778 (92.2%) | **OR 1.69** (0.49 to 5.81) | **30 more per 1,000** (from 69 fewer to 64 more) | ⨁◯◯◯ Very low |  |
| Explanation: b. Unclear risk of bias; c. One study was of weak quality; d. One study has a small sample size | | | | | | | | | | | | |
| **Tobacco and Alcohol Use Control** | | | | | | | | | | | | |
| 1 | observational studies | Serious ^a^ | not serious | not serious | not serious | none | 40/40 (100.0%) | 42/46 (91.3%) | **OR 1.09** (0.99 to 1.21) | **7 more per 1,000** (from 1 fewer to 14 more) | ⨁◯◯◯ Very low | NOT IMPORTANT |
| Explanation: a. Study has moderate quality | | | | | | | | | | | | |
| **Incentives** | | | | | | | | | | | | |
| 4 | 2RCTs, 2non-RCTs | not serious ^a^ | not serious | not serious | not serious | publication bias strongly suspected ^b^ | 201/355 (56.6%) | 196/473 (41.4%) | **OR 2.86** (1.25 to 6.50) | **255 more per 1,000** (from 55 more to 407 more) | ⨁⨁⨁◯ Moderate |  |
| 2 | Randomized trials | Serious ^c^ | not serious | not serious | not serious | none | 99/237 (41.8%) | 60/234 (25.6%) | **OR 1.64** (1.25 to 2.13) | **105 more per 1,000** (from 45 more to 167 more) | ⨁⨁⨁◯ Moderate | IMPORTANT |
| 2 | observational studies | Serious ^d^ | not serious | not serious | not serious | none | 102/118 (86.4%) | 136/239 (56.9%) | **OR 4.79** (1.28 to 17.94) | **294 more per 1,000** (from 59 more to 390 more) | ⨁◯◯◯ Very low | IMPORTANT |
| Explanation: b. selection bias and high attrition bias; c. High withdrawal in one study | | | | | | | | | | | | |
| **Home-based Care** | | | | | | | | | | | | |
| 2 | Randomized trials | Serious ^a^ | not serious | not serious | not serious | none | 372/838 (44.4%) | 339/833 (40.7%) | **OR 2.24** (0.42 to 11.88) | **199 more per 1,000** (from 183 fewer to 484 more) | ⨁⨁⨁◯ Moderate |  |
| Explanations: b. Unclear risk of selection bias and participants & personnel were not blinded; c. small sample size | | | | | | | | | | | | |
| **Education and Counseling** | | | | | | | | | | | | |
| 1 | Randomized trials | not serious ^a^ | not serious | not serious | not serious | none | 75/8874 (0.8%) | 25/9158 (0.3%) | **OR 3.10** (1.97 to 4.86) | **6 more per 1,000** (from 3 more to 10 more) | ⨁⨁⨁⨁ High | IMPORTANT |
| Explanation: Overall low risk of bias in both studies | | | | | | | | | | | | |
| **Digital interventions** | | | | | | | | | | | | |
| 4 | Randomized trials | not serious | Serious ^a^ | not serious | not serious ^b^ | none | 579/1162 (49.8%) | 291/833 (34.9%) | **OR 1.10** (1.04 to 1.17) | **22 more per 1,000** (from 9 more to 36 more) | ⨁⨁⨁◯ Moderate | IMPORTANT |
| Explanations: a. Weak association with outcome; b. small sample size | | | | | | | | | | | | |
| **Active Case Finding** | | | | | | | | | | | | |
| 5 | 3RCTs, 2 non-RCTs | Serious ^a^ | not serious | not serious | not serious | none | 27330/29739 (91.9%) | 14212/15562 (91.3%) | **OR 1.42** (0.84 to 2.39) | **24 more per 1,000** (from 15 fewer to 49 more) | ⨁⨁⨁◯ Moderate |  |
| 3 | Randomized trials | Serious ^b^ | not serious | not serious | not serious | none | 2478/2786 (88.9%) | 2435/2784 (87.5%) | **OR 1** (1 to 1) | **0 fewer per 1,000** (from 0 fewer to 0 fewer) | ⨁⨁⨁◯ Moderate |  |
| 2 | observational studies | not serious | not serious ^c^ | not serious | Serious ^d^ | none | 24852/26953 (92.2%) | 11777/12778 (92.2%) | **OR 1.69** (0.49 to 5.87) | **30 more per 1,000** (from 69 fewer to 64 more) | ⨁◯◯◯ Very low |  |
| Explanations: a. 3 RCT and 2 Non-RCTs, b. Unclear risk of bias, c. One study was of weak quality, d. One study has a small sample size | | | | | | | | | | | | |
| **Community-based interventions** | | | | | | | | | | | | |
| 4 | 2RCTs, 2non-RCTs | Serious ^a^ | not serious | not serious | not serious | none | 768/2425 (31.7%) | 154/5274 (2.9%) | **OR 9.91** (1.86 to 52.74) | **200 more per 1,000** (from 24 more to 584 more) | ⨁⨁⨁◯ Moderate | IMPORTANT |
| 2 | Randomized trials | not serious | not serious | not serious | Serious ^b^ | none | 176/202 (87.1%) | 139/180 (77.2%) | **OR 2.93** (0.32 to 26.82) | **136 more per 1,000** (from 252 fewer to 217 more) | ⨁⨁⨁◯ Moderate | IMPORTANT |
| 2 | observational studies | not serious | not serious | not serious | Serious ^b^ | very strong association | 592/2223 (26.6%) | 15/1685 (0.9%) | **OR 26.93** (16.21 to 44.75) | **186 more per 1,000** (from 118 more to 278 more) | ⨁⨁⨁◯ Moderate |  |
| Explanations: a. 2RCT, 2 non-RCTs, b. small sample size | | | | | | | | | | | | |
| **Multi-sector Collaborations** | | | | | | | | | | | | |
| 3 | 1RCT, 2 non-RCTs | Serious ^a^ | not serious | not serious | not serious | none | 841/1155 (72.8%) | 397/760 (52.2%) | **OR 3.25** (2.05 to 5.14) | **258 more per 1,000** (from 169 more to 327 more) | ⨁⨁⨁◯ Moderate |  |
| 1 | Randomized trials | not serious | not serious | not serious | Serious ^b^ | none | 55/94 (58.5%) | 40/95 (42.1%) | **OR 1.39** (1.04 to 1.86) | **82 more per 1,000** (from 10 more to 154 more) | ⨁⨁⨁◯ Moderate | CRITICAL |
| 2 | observational studies | not serious | not serious | not serious | not serious | strong association | 786/1061 (74.1%) | 357/665 (53.7%) | **OR 3.80** (2.73 to 5.28) | **278 more per 1,000** (from 223 more to 323 more) | ⨁⨁⨁◯ Moderate | CRITICAL |
| Explanations: a. 1 RCT and 2 Non-RCTs, b. small sample size | | | | | | | | | | | | |
| **Patient navigator and referral system** | | | | | | | | | | | | |
| 4 | 1RCT, 3 non-RCTs | not serious | not serious | not serious | Serious ^a^ | none | 21777/64580 (33.7%) | 7258/64472 (11.3%) | **OR 1.65** (0.85 to 3.21) | **61 more per 1,000** (from 15 fewer to 177 more) | ⨁⨁⨁◯ Moderate |  |
| 1 | Randomized trials | not serious | not serious | not serious | not serious | none | 212/967 (21.9%) | 197/932 (21.1%) | **OR 1.04** (0.87 to 1.23) | **7 more per 1,000** (from 22 fewer to 37 more) | ⨁⨁⨁⨁ High |  |
| 3 | observational studies | not serious | not serious | not serious | not serious | publication bias strongly suspected ^b^ | 21565/63613 (33.9%) | 7061/63540 (11.1%) | **OR 1.92** (0.84 to 4.43) | **82 more per 1,000** (from 16 fewer to 245 more) | ⨁◯◯◯ Very low |  |
| Explanations: a. One study had very large sample size, b. Evidence of publication bias per funnel plot | | | | | | | | | | | | |
| **Mixed Interventions** | | | | | | | | | | | | |
| 7 | 4RCT, 3 non-RCTs | not serious ^a^ | not serious | not serious | not serious | publication bias strongly suspected ^b^ | 1685/5126 (32.9%) | 1385/6596 (21.0%) | **OR 5.71** (0.94 to 34.75) | **393 more per 1,000** (from 10 fewer to 692 more) | ⨁⨁⨁◯ Moderate | IMPORTANT |
| 4 | Randomized trials | not serious | not serious | not serious | Serious ^c^ | none | 1312/4650 (28.2%) | 1330/6328 (21.0%) | **OR 1.44** (0.44 to 2.34) | **67 more per 1,000** (from 105 fewer to 174 more) | ⨁⨁⨁◯ Moderate |  |
| 3 | observational studies | not serious | Serious ^d^ | not serious | not serious | none | 373/476 (78.4%) | 55/268 (20.5%) | **OR 22.27** (0.52 to 954.74) | **647 more per 1,000** (from 87 fewer to 791 more) | ⨁◯◯◯ Very low | NOT IMPORTANT |
| Explanations: a. 4 RCTs and 3 non-RCTs, b. Possible publication per funnel plot, c. One study has a small sample size, d. Very large OR | | | | | | | | | | | | |
| **Staff Training** | | | | | | | | | | | | |
| 3 | Randomized trials | not serious | not serious | not serious | not serious | publication bias strongly suspected ^a^ | 4429/17340 (25.5%) | 6898/24186 (28.5%) | **OR 1.76** (1.00 to 3.08) | **127 more per 1,000** (from 0 fewer to 266 more) | ⨁⨁⨁◯ Moderate |  |
| a. Possible publication bias per funnel plot | | | | | | | | | | | | |
| **Improve facility TB care** | | | | | | | | | | | | |
| 1 | Randomized trials | not serious | not serious | not serious | Serious ^a^ | none | 13/159 (8.2%) | 14/162 (8.6%) | **OR 0.95** (0.46 to 1.95) | **4 fewer per 1,000** (from 45 fewer to 69 more) | ⨁⨁⨁◯ Moderate |  |
| Explanations: b. One study has a small sample size c. Very large OR | | | | | | | | | | | | |

| Summary of GRADE Assessment of Quality of Evidence Certainty for each Outcome of interest for TB cure | | | | | | | | | | | | |
| --- | --- | --- | --- | --- | --- | --- | --- | --- | --- | --- | --- | --- |
| **Certainty assessment** | | | | | | | **№ of patients** | | **Effect** | | **Certainty** | **Importance** |
| **№ of studies** | **Study design** | **Risk of bias** | **Inconsistency** | **Indirectness** | **Imprecision** | **Other considerations** | **[Intervention]** | **[Standard-of-care]** | **Relative (95% CI)** | **Absolute (95% CI)** |  |  |
| **Digital Interventions** | | | | | | | | | | | | |
| 7 | 5RCTs, 2 non-RCTs | not serious ^a^ | not serious | not serious | Serious ^b^ | none | 1440/3368 (42.8%) | 1599/3510 (45.6%) | **OR 2.17** (0.65 to 7.25) | **189 more per 1,000** (from 103 fewer to 403 more) | ⨁⨁⨁◯ Moderate | IMPORTANT |
| 5 | Randomized trials | not serious ^c^ | not serious | not serious | Serious ^b^ | none | 1327/2474 (53.6%) | 1275/2453 (52.0%) | **OR 2.74** (0.68 to 10.97) | **228 more per 1,000** (from 96 fewer to 403 more) | ⨁⨁⨁◯ Moderate | CRITICAL |
| 2 | observational studies | not serious | not serious | not serious | Serious ^b^ | very strong association | 113/894 (12.6%) | 324/1057 (30.7%) | **OR 1.27** (0.06 to 26.89) | **53 more per 1,000** (from 281 fewer to 616 more) | ⨁⨁⨁◯ Moderate |  |
| Explanations: b. Some studies have unclear risk of bias, c. Some studies have small sample size | | | | | | | | | | | | |
| **Home-based Care** | | | | | | | | | | | | |
| 2 | observational studies | not serious | not serious | not serious | Serious ^a^ | none | 25/64 (39.1%) | 8/73 (11.0%) | **OR 4.94** (0.69 to 35.18) | **269 more per 1,000** (from 31 fewer to 703 more) | ⨁◯◯◯ Very low | IMPORTANT |
| Explanation: a. small sample size | | | | | | | | | | | | |
| **Incentives** | | | | | | | | | | | | |
| 5 | 2RCTs, 3 non-RCTs | not serious ^a^ | not serious | not serious | not serious | none | 12053/17472 (69.0%) | 4665/9893 (47.2%) | **OR 1.62** (106.00 to 2.48) | **120 more per 1,000** (from 217 more to 518 more) | ⨁⨁⨁⨁ High | IMPORTANT |
| 2 | Randomized trials | not serious | not serious | not serious | not serious | none | 766/2242 (34.2%) | 763/2131 (35.8%) | **OR 1.24** (0.60 to 2.54) | **51 more per 1,000** (from 107 fewer to 228 more) | ⨁⨁⨁⨁ High | IMPORTANT |
| 3 | observational studies | not serious | not serious | not serious | not serious | very strong association | 11287/15230 (74.1%) | 3902/7762 (50.3%) | **OR 1.97** (1.21 to 3.19) | **163 more per 1,000** (from 47 more to 261 more) | ⨁⨁⨁⨁ High | IMPORTANT |
| **Education and Counseling** | | | | | | | | | | | | |
| 4 | 2RCTs, 2 non-RCTs | not serious | not serious | not serious | Serious ^a^ | none | 210/426 (49.3%) | 155/282 (55.0%) | **OR 2.08** (1.11 to 3.88) | **168 more per 1,000** (from 26 more to 276 more) | ⨁⨁⨁◯ Moderate | IMPORTANT |
| 2 | Randomized trials | not serious | not serious | not serious | Serious ^a^ | strong association | 152/180 (84.4%) | 131/189 (69.3%) | **OR 3.45** (1.92 to 6.18) | **193 more per 1,000** (from 119 more to 240 more) | ⨁⨁⨁⨁ High | IMPORTANT |
| 2 | observational studies | not serious | not serious | not serious | Serious ^a^ | none | 58/246 (23.6%) | 24/93 (25.8%) | **OR 1.31** (0.87 to 1.96) | **55 more per 1,000** (from 26 fewer to 147 more) | ⨁◯◯◯ Very low | IMPORTANT |
| Explanation: a. small sample size | | | | | | | | | | | | |
| **Tobacco and Alcohol Use Control** | | | | | | | | | | | | |
| 3 | 2RCTs, 1 non-RCT | not serious | not serious | not serious | Serious ^a^ | none | 252/690 (36.5%) | 183/992 (18.4%) | **OR 2.39** (1.06 to 5.40) | **166 more per 1,000** (from 9 more to 365 more) | ⨁⨁⨁◯ Moderate |  |
| 2 | Randomized trials | not serious | not serious | not serious | Serious ^a^ | none | 220/650 (33.8%) | 159/946 (16.8%) | **OR 2.06** (0.65 to 6.54) | **126 more per 1,000** (from 52 fewer to 401 more) | ⨁⨁⨁◯ Moderate | IMPORTANT |
| 1 | observational studies | not serious | not serious | not serious | not serious | strong association | 32/40 (80.0%) | 24/46 (52.2%) | **OR 1.53** (1.12 to 2.11) | **104 more per 1,000** (from 28 more to 175 more) | ⨁⨁⨁◯ Moderate |  |
| Explanation: a. small sample size | | | | | | | | | | | | |
| **Active Case Finding** | | | | | | | | | | | | |
| 1 | observational studies | not serious | not serious | not serious | not serious | strong association | 2501/2934 (85.2%) | 1852/2298 (80.6%) | **OR 1.06** (1.03 to 1.08) | **9 more per 1,000** (from 5 more to 12 more) | ⨁⨁⨁◯ Moderate | NOT IMPORTANT |
| **Community-based Interventions** | | | | | | | | | | | | |
| 9 | 5RCTs, 4 non-RCTs | Serious ^a^ | not serious | not serious | not serious | none | 14845/25044 (59.3%) | 2619/8126 (32.2%) | **OR 2.53** (1.92 to 3.35) | **224 more per 1,000** (from 155 more to 292 more) | ⨁⨁⨁◯ Moderate |  |
| 5 | Randomized trials | Serious ^b^ | not serious | not serious | Serious ^c^ | strong association | 522/730 (71.5%) | 311/558 (55.7%) | **OR 1.67** (1.08 to 2.57) | **120 more per 1,000** (from 19 more to 207 more) | ⨁⨁⨁◯ Moderate |  |
| 4 | observational studies | not serious | not serious | not serious | not serious ^c^ | strong association | 14323/24314 (58.9%) | 2308/7568 (30.5%) | **OR 2.92** (2.08 to 4.10) | **257 more per 1,000** (from 172 more to 338 more) | ⨁⨁⨁◯ Moderate |  |
| Explanation: b. Both studies have high risk of bias; c. small sample size | | | | | | | | | | | | |
| **Improved Facility-based TB Care** | | | | | | | | | | | | |
| 2 | Randomized trials | not serious | very serious ^a^ | not serious | not serious | none | 80/359 (22.3%) | 41/215 (19.1%) | **OR 0.81** (0.10 to 6.84) | **30 fewer per 1,000** (from 168 fewer to 426 more) | ⨁⨁◯◯ Low | IMPORTANT |
| Explanation: a. Inconsistent OR | | | | | | | | | | | | |
| **Staff Training** | | | | | | | | | | | | |
| 3 | Randomized trials | not serious | not serious | not serious | Serious ^a^ | none | 343/725 (47.3%) | 303/714 (42.4%) | **OR 3.13** (0.82 to 11.93) | **273 more per 1,000** (from 48 fewer to 474 more) | ⨁⨁⨁◯ Moderate | IMPORTANT |
| Explanation: b. Studies have inconsistent ORs | | | | | | | | | | | | |
| **Mixed Interventions** | | | | | | | | | | | | |
| 3 | Randomized trials | not serious ^a^ | not serious | not serious | not serious | none | 680/833 (81.6%) | 548/849 (64.5%) | **OR 1.19** (1.13 to 1.26) | **39 more per 1,000** (from 27 more to 51 more) | ⨁⨁⨁⨁ High | IMPORTANT |

| Summary of GRADE Assessment of Quality of Evidence Certainty for each Outcome of interest for treatment completion | | | | | | | | | | | | |
| --- | --- | --- | --- | --- | --- | --- | --- | --- | --- | --- | --- | --- |
| **Certainty assessment** | | | | | | | **№ of patients** | | **Effect** | | **Certainty** | **Importance** |
| **№ of studies** | **Study design** | **Risk of bias** | **Inconsistency** | **Indirectness** | **Imprecision** | **Other considerations** | **[Intervention]** | **[Standard-of-care]** | **Relative (95% CI)** | **Absolute (95% CI)** |  |  |
| **Digital Interventions** | | | | | | | | | | | | |
| 9 | 6RCTs, 3 non-RCTs | not serious ^a^ | Serious ^b^ | not serious | not serious | none | 1706/3772 (45.2%) | 1956/4204 (46.5%) | **OR 1.14** (0.87 to 1.48) | **33 more per 1,000** (from 34 fewer to 98 more) | ⨁⨁⨁◯ Moderate |  |
| 6 | Randomized trials | not serious | Serious ^b^ | not serious | not serious ^c^ | none | 1021/2670 (38.2%) | 1029/2674 (38.5%) | **OR 1.05** (1.01 to 1.10) | **12 more per 1,000** (from 2 more to 23 more) | ⨁⨁⨁◯ Moderate | CRITICAL |
| 3 | observational studies | not serious | not serious | not serious | not serious | none | 685/1102 (62.2%) | 927/1530 (60.6%) | **OR 1.11** (0.60 to 2.04) | **25 more per 1,000** (from 126 fewer to 152 more) | ⨁⨁◯◯ Low |  |
| Explanation: b. 1 study has inconsistent OR with other studies; c. small sample size in one study | | | | | | | | | | | | |
| **Home-based Care** | | | | | | | | | | | | |
| 3 | observational studies | not serious | very serious ^a^ | not serious | Serious ^b^ | none | 339/400 (84.8%) | 299/409 (73.1%) | **OR 1.32** (0.17 to 10.20) | **51 more per 1,000** (from 415 fewer to 234 more) | ⨁◯◯◯ Very low |  |
| Explanation: a. 2 studies have inconsistent ORs; b. small sample size | | | | | | | | | | | | |
| **Incentives** | | | | | | | | | | | | |
| 12 | 7RCT, 5 non-RCT | Serious ^a, b^ | not serious | not serious | not serious | none | 7762/9514 (81.6%) | 7731/9970 (77.5%) | **OR 1.37** (1.10 to 1.71) | **50 more per 1,000** (from 16 more to 80 more) | ⨁⨁⨁◯ Moderate | CRITICAL |
| 7 | Randomized trials | Serious ^b^ | not serious | not serious | not serious | none | 6632/8218 (80.7%) | 4749/6401 (74.2%) | **OR 1.18** (0.95 to 1.47) | **30 more per 1,000** (from 10 fewer to 67 more) | ⨁⨁⨁◯ Moderate | CRITICAL |
| 5 | observational studies | not serious | not serious | not serious | not serious | very strong association | 1130/1296 (87.2%) | 2982/3596 (82.9%) | **OR 1.76** (1.19 to 2.62) | **66 more per 1,000** (from 23 more to 98 more) | ⨁⨁⨁⨁ High | CRITICAL |
| Explanation: b. 2 studies have high risks of selection, performance, and detection bias | | | | | | | | | | | | |
| **Counseling and Education** | | | | | | | | | | | | |
| 8 | 5RCTS, 3 non-RCT | not serious ^a^ | not serious | not serious | not serious | none | 808/1440 (56.1%) | 579/1249 (46.4%) | **OR 1.48** (1.07 to 2.03) | **98 more per 1,000** (from 17 more to 173 more) | ⨁⨁⨁⨁ High | IMPORTANT |
| 5 | Randomized trials | not serious | not serious | not serious | not serious | strong association | 650/1172 (55.5%) | 541/1145 (47.2%) | **OR 1.47** (1.00 to 2.16) | **96 more per 1,000** (from 0 fewer to 187 more) | ⨁⨁⨁⨁ High |  |
| 3 | observational studies | not serious ^b^ | not serious | not serious | Serious ^c^ | none | 158/268 (59.0%) | 38/104 (36.5%) | **OR 1.28** (0.98 to 1.66) | **59 more per 1,000** (from 5 fewer to 123 more) | ⨁◯◯◯ Very low |  |
| Explanations: b. moderate quality; c. small sample size | | | | | | | | | | | | |
| **Reminders and Tracers** | | | | | | | | | | | | |
| 6 | Randomized trials | not serious | not serious | not serious | not serious | none | 586/832 (70.4%) | 673/960 (70.1%) | **OR 1.03** (1.00 to 1.07) | **6 more per 1,000** (from 0 fewer to 14 more) | ⨁⨁⨁⨁ High | IMPORTANT |
| **Tobacco and Alcohol Use Control** | | | | | | | | | | | | |
| 3 | 1RCT, 2 non-RCTs | Serious ^a^ | Serious ^b^ | not serious | not serious | none | 40/528 (7.6%) | 43/827 (5.2%) | **OR 0.97** (0.35 to 2.69) | **1 fewer per 1,000** (from 33 fewer to 77 more) | ⨁⨁◯◯ Low |  |
| 1 | Randomized trials | not serious ^c^ | not serious | not serious | not serious | none | 29/455 (6.4%) | 22/741 (3.0%) | **OR 2.15** (1.25 to 3.69) | **32 more per 1,000** (from 7 more to 72 more) | ⨁⨁⨁⨁ High |  |
| 2 | observational studies | not serious | not serious | not serious | Serious ^d^ | none | 11/73 (15.1%) | 21/86 (24.4%) | **OR 0.62** (0.32 to 1.20) | **77 fewer per 1,000** (from 150 fewer to 35 more) | ⨁◯◯◯ Very low |  |
| Explanation: b. 2 included studies have distinct ORs; c. Unclear risk of bias; d. small sample size | | | | | | | | | | | | |
| **Staff Training** | | | | | | | | | | | | |
| 3 | Randomized trials | not serious ^a^ | Serious ^b^ | not serious | not serious | none | 430/1089 (39.5%) | 363/967 (37.5%) | **OR 0.68** (0.30 to 1.57) | **85 fewer per 1,000** (from 223 fewer to 110 more) | ⨁⨁⨁◯ Moderate | IMPORTANT |
| Explanation: b. 1 study had a distinct OR. | | | | | | | | | | | | |
| **Active Case Finding** | | | | | | | | | | | | |
| 3 | 2RCTs, 1 non-RCT | Serious ^a^ | not serious | not serious | not serious | none | 215/3069 (7.0%) | 363/2428 (15.0%) | **OR 0.53** (0.24 to 1.16) | **64 fewer per 1,000** (from 109 fewer to 20 more) | ⨁⨁⨁◯ Moderate | IMPORTANT |
| 2 | Randomized trials | not serious | not serious | not serious | Serious ^b^ | none | 113/135 (83.7%) | 112/130 (86.2%) | **OR 0.97** (0.88 to 1.08) | **4 fewer per 1,000** (from 16 fewer to 9 more) | ⨁⨁⨁◯ Moderate | IMPORTANT |
| 1 | observational studies | not serious | not serious | not serious | not serious | none | 102/2934 (3.5%) | 251/2298 (10.9%) | **OR 0.32** (0.25 to 0.40) | **71 fewer per 1,000** (from 79 fewer to 62 fewer) | ⨁⨁◯◯ Low |  |
| Explanation: b. small sample size | | | | | | | | | | | | |
| **Case Management** | | | | | | | | | | | | |
| 2 | Randomized trials | not serious ^a^ | Serious ^b^ | not serious | Serious ^c^ | none | 53/64 (82.8%) | 44/64 (68.8%) | **OR 3.17** (0.24 to 41.55) | **187 more per 1,000** (from 342 fewer to 302 more) | ⨁⨁◯◯ Low | IMPORTANT |
| Explanations: b. Studies have inconsistent ORs; c. small sample size | | | | | | | | | | | | |
| **Community-based Interventions** | | | | | | | | | | | | |
| 9 | 4RCTs, 5 non-RCTs | Serious ^a^ | Serious ^b^ | not serious | not serious | none | 8429/24659 (34.2%) | 3212/7771 (41.3%) | **OR 1.01** (0.60 to 1.72) | **2 more per 1,000** (from 116 fewer to 135 more) | ⨁⨁◯◯ Low |  |
| 4 | Randomized trials | Serious ^c^ | not serious | not serious | Serious ^d^ | none | 111/644 (17.2%) | 97/472 (20.6%) | **OR 0.85** (0.69 to 1.06) | **25 fewer per 1,000** (from 54 fewer to 10 more) | ⨁⨁◯◯ Low | CRITICAL |
| 5 | observational studies | not serious | Serious ^b^ | not serious | not serious | all plausible residual confounding would reduce the demonstrated effect | 8362/24378 (34.3%) | 3165/7632 (41.5%) | **OR 1.04** (0.56 to 1.96) | **10 more per 1,000** (from 131 fewer to 167 more) | ⨁⨁◯◯ Low | CRITICAL |
| Explanations: b. Studies have inconsistent ORs; c. Both studies have unclear risk of bias; d. small sample size | | | | | | | | | | | | |
| **Improved Facility-based TB Care** | | | | | | | | | | | | |
| 3 | observational studies | not serious ^a^ | not serious | not serious | not serious | none | 234/410 (57.1%) | 167.28/277 (60.4%) | **OR 1.04** (0.98 to 1.10) | **9 more per 1,000** (from 5 fewer to 23 more) | ⨁⨁◯◯ Low | IMPORTANT |
| Explanation: a. Studies were of moderate quality | | | | | | | | | | | | |
| **Mixed Interventions** | | | | | | | | | | | | |
| 4 | Randomized trials | not serious ^a^ | Serious ^b^ | not serious | not serious | none | 210/1024 (20.5%) | 232/1038 (22.4%) | **OR 1.06** (0.97 to 1.16) | **10 more per 1,000** (from 5 fewer to 27 more) | ⨁⨁⨁◯ Moderate | IMPORTANT |
| Explanations: a. Low and unclear risk of bias; b. Studies have inconsistent ORs | | | | | | | | | | | | |

| Summary of GRADE Assessment of Quality of Evidence Certainty for each Outcome of interest for treatment success | | | | | | | | | | | | |
| --- | --- | --- | --- | --- | --- | --- | --- | --- | --- | --- | --- | --- |
| **Certainty assessment** | | | | | | | **№ of patients** | | **Effect** | | **Certainty** | **Importance** |
| **№ of studies** | **Study design** | **Risk of bias** | **Inconsistency** | **Indirectness** | **Imprecision** | **Other considerations** | **[Intervention]** | **[Standard-of-care]** | **Relative (95% CI)** | **Absolute (95% CI)** |  |  |
| **Digital Interventions** | | | | | | | | | | | | |
| 8 | 7RCTs, 1 non-RCT | Serious ^a^ | not serious | not serious | not serious | none | 3302/4201 (78.6%) | 3462/4406 (78.6%) | **OR 1.33** (0.81 to 2.20) | **44 more per 1,000** (from 38 fewer to 104 more) | ⨁⨁⨁◯ Moderate | IMPORTANT |
| 7 | Randomized trials | Serious ^a^ | not serious | not serious | not serious | none | 2697/3331 (81.0%) | 2726/3445 (79.1%) | **OR 1.08** (0.96 to 1.22) | **12 more per 1,000** (from 7 fewer to 31 more) | ⨁⨁⨁◯ Moderate |  |
| 1 | observational studies | not serious | not serious | not serious | not serious | none | 605/870 (69.5%) | 736/961 (76.6%) | **OR 0.91** (0.86 to 0.96) | **17 fewer per 1,000** (from 28 fewer to 7 fewer) | ⨁⨁◯◯ Low |  |
| Explanation: a. 2 studies had high risk of bias | | | | | | | | | | | | |
| **Home-based Care** | | | | | | | | | | | | |
| 3 | 1RCT, 2 non-RCTs | Serious ^a^ | not serious | not serious | not serious | none | 267/304 (87.8%) | 215/313 (68.7%) | **OR 1.24** (1.15 to 1.34) | **44 more per 1,000** (from 29 more to 59 more) | ⨁⨁⨁◯ Moderate |  |
| 1 | Randomized trials | not serious | not serious | not serious | not serious | strong association | 226/240 (94.2%) | 184/240 (76.7%) | **OR 1.23** (1.14 to 1.33) | **35 more per 1,000** (from 23 more to 47 more) | ⨁⨁⨁⨁ High | IMPORTANT |
| 2 | observational studies | not serious | not serious | not serious | Serious ^b^ | strong association | 41/64 (64.1%) | 31/73 (42.5%) | **OR 1.49** (1.08 to 2.05) | **99 more per 1,000** (from 19 more to 177 more) | ⨁⨁◯◯ Low | IMPORTANT |
| Explanation: b. small sample sizes | | | | | | | | | | | | |
| **Incentives** | | | | | | | | | | | | |
| 5 | 3RCTs, 2 non-RCTs | not serious ^a^ | not serious | not serious | not serious | none | 2490/3132 (79.5%) | 1836/2575 (71.3%) | **OR 1.08** (1.05 to 1.11) | **15 more per 1,000** (from 10 more to 21 more) | ⨁⨁⨁⨁ High | CRITICAL |
| 3 | Randomized trials | not serious ^b^ | not serious | not serious | not serious | strong association | 1727/2277 (75.8%) | 1517/2173 (69.8%) | **OR 1.08** (1.05 to 1.12) | **16 more per 1,000** (from 10 more to 23 more) | ⨁⨁⨁⨁ High |  |
| 2 | observational studies | not serious | not serious | not serious | not serious | none | 763/855 (89.2%) | 319/402 (79.4%) | **OR 1.83** (1.11 to 3.03) | **82 more per 1,000** (from 17 more to 127 more) | ⨁⨁◯◯ Low |  |
| b. unclear risk of bias | | | | | | | | | | | | |
| **Education and Counseling** | | | | | | | | | | | | |
| 5 | 2RCTs, 3 non-RCTs | Serious ^a^ | not serious | not serious | not serious | none | 402/475 (84.6%) | 204/311 (65.6%) | **OR 3.24** (1.88 to 5.55) | **205 more per 1,000** (from 126 more to 258 more) | ⨁⨁⨁◯ Moderate |  |
| 2 | Randomized trials | Serious ^b^ | not serious | not serious | Serious ^c^ | strong association | 186/207 (89.9%) | 142/207 (68.6%) | **OR 4.85** (2.75 to 8.58) | **228 more per 1,000** (from 171 more to 263 more) | ⨁⨁⨁◯ Moderate | CRITICAL |
| 3 | observational studies | not serious | not serious | not serious | not serious | none | 216/268 (80.6%) | 62/104 (59.6%) | **OR 1.28** (1.08 to 1.51) | **58 more per 1,000** (from 18 more to 94 more) | ⨁⨁◯◯ Low |  |
| Explanations: b. Unclear risk of selection bias; c. small sample size | | | | | | | | | | | | |
| **Tobacco and Alcohol Use Control** | | | | | | | | | | | | |
| 6 | 4RCTs, 2 non-RCTs | not serious ^a^ | not serious | not serious | not serious | none | 580/1022 (56.8%) | 542/1401 (38.7%) | **OR 1.91** (0.89 to 4.08) | **160 more per 1,000** (from 27 fewer to 333 more) | ⨁⨁⨁⨁ High |  |
| 4 | Randomized trials | Serious ^b^ | not serious | not serious | not serious | strong association | 537/949 (56.6%) | 497/1315 (37.8%) | **OR 2.03** (0.95 to 4.32) | **174 more per 1,000** (from 12 fewer to 346 more) | ⨁⨁⨁⨁ High | IMPORTANT |
| 2 | observational studies | not serious | not serious | not serious | Serious ^c^ | none | 43/73 (58.9%) | 45/86 (52.3%) | **OR 2.02** (0.10 to 42.91) | **166 more per 1,000** (from 424 fewer to 456 more) | ⨁◯◯◯ Very low |  |
| Explanations: b. All studies had high risk of bias; c. small sample size | | | | | | | | | | | | |
| Reminders and Tracers | | | | | | | | | | | | |
| 2 | Randomized trials | Serious ^a^ | not serious | not serious | not serious | none | 184/201 (91.5%) | 176/207 (85.0%) | **OR 1.09** (1.01 to 1.16) | **11 more per 1,000** (from 1 more to 18 more) | ⨁⨁⨁◯ Moderate |  |
| Explanations: a. one study has some unclear risks of bias | | | | | | | | | | | | |
| **Active Case Finding** | | | | | | | | | | | | |
| 1 | observational studies | Serious ^a^ | not serious | not serious | not serious | none | 2603/2934 (88.7%) | 2103/2298 (91.5%) | **OR 0.97** (0.95 to 0.99) | **2 fewer per 1,000** (from 4 fewer to 1 fewer) | ⨁◯◯◯ Very low | NOT IMPORTANT |
| Explanations: a. Weak quality | | | | | | | | | | | | |
| **Case Management** | | | | | | | | | | | | |
| 2 | Randomized trials | Serious ^a^ | not serious | not serious | Serious ^b^ | none | 52/64 (81.3%) | 44/64 (68.8%) | **OR 2.63** (0.36 to 15.32) | **165 more per 1,000** (from 246 fewer to 284 more) | ⨁⨁◯◯ Low | IMPORTANT |
| Explanations: a. 1 study had high risk of bias; b. small sample size | | | | | | | | | | | | |
| **Community-based Interventions** | | | | | | | | | | | | |
| 10 | 5RCTs, 5 non-RCTs | Serious ^a^ | not serious | not serious | not serious | none | 24799/26888 (92.2%) | 6533/9113 (71.7%) | **OR 2.91** (2.01 to 4.21) | **164 more per 1,000** (from 119 more to 197 more) | ⨁⨁⨁◯ Moderate | CRITICAL |
| 5 | Randomized trials | not serious ^b^ | not serious | not serious | Serious ^c^ | none | 685/803 (85.3%) | 514/693 (74.2%) | **OR 2.69** (1.14 to 6.33) | **144 more per 1,000** (from 24 more to 206 more) | ⨁⨁⨁◯ Moderate | IMPORTANT |
| 5 | observational studies | not serious | not serious | not serious | not serious | very strong association | 24114/26085 (92.4%) | 6019/8420 (71.5%) | **OR 3.38** (2.48 to 4.61) | **180 more per 1,000** (from 147 more to 206 more) | ⨁⨁⨁⨁ High | CRITICAL |
| Explanations: a. 5 non-RCTs, b. Low and unclear risk of bias; c. small sample size | | | | | | | | | | | | |
| **Improved Facility-based TB Care** | | | | | | | | | | | | |
| 2 | observational studies | not serious | Serious ^a^ | not serious | not serious | none | 263/359 (73.3%) | 150/215 (69.8%) | **OR 0.59** (0.04 to 8.41) | **121 fewer per 1,000** (from 613 fewer to 253 more) | ⨁◯◯◯ Very low | NOT IMPORTANT |
| Explanation: a. Inconsistent ORs | | | | | | | | | | | | |
| **Staff Training** | | | | | | | | | | | | |
| 3 | 2RCTs, 1 non-RCT | Serious ^a^ | not serious | not serious | not serious | none | 526/824 (63.8%) | 536/855 (62.7%) | **OR 1.04** (0.97 to 1.11) | **9 more per 1,000** (from 7 fewer to 24 more) | ⨁⨁⨁◯ Moderate | IMPORTANT |
| 2 | Randomized trials | not serious ^b^ | not serious | not serious | not serious | none | 404/668 (60.5%) | 398/657 (60.6%) | **OR 1.00** (0.92 to 1.09) | **0 fewer per 1,000** (from 20 fewer to 20 more) | ⨁⨁⨁⨁ High |  |
| 1 | observational studies | not serious | not serious | not serious | Serious ^c^ | none | 122/156 (78.2%) | 138/198 (69.7%) | **OR 1.12** (0.99 to 1.27) | **23 more per 1,000** (from 2 fewer to 48 more) | ⨁◯◯◯ Very low |  |
| Explanations: b. Low risk of bias; c. small sample size | | | | | | | | | | | | |
| **Mixed Interventions** | | | | | | | | | | | | |
| 3 | Randomized trials | not serious ^a^ | not serious | not serious | Serious ^b^ | none | 728/833 (87.4%) | 633/849 (74.6%) | **OR 1.14** (1.09 to 1.19) | **24 more per 1,000** (from 16 more to 32 more) | ⨁⨁⨁◯ Moderate | IMPORTANT |
| Explanation: b. One study had a small sample size | | | | | | | | | | | | |

# Table H in S1 File PRISMA checklist for protocol

| **PRISMA-P (Preferred Reporting Items for Systematic Review and Meta-Analysis Protocols) 2015 checklist: recommended items to address in a systematic review protocol*** | | | |
| --- | --- | --- | --- |
| **Section and topic** | **Item No** | **Checklist item** | **Pages** |
| **ADMINISTRATIVE INFORMATION** | | |  |
| Title: |  |  |  |
| Identification | 1a | Identify the report as a protocol of a systematic review |  |
| Update | 1b | If the protocol is for an update of a previous systematic review, identify as such |  |
| Registration | 2 | If registered, provide the name of the registry (such as PROSPERO) and registration number |  |
| Authors: |  |  |  |
| Contact | 3a | Provide name, institutional affiliation, the e-mail address of all protocol authors; provide the physical mailing address of the corresponding author |  |
| Contributions | 3b | Describe contributions of protocol authors and identify the guarantor of the review |  |
| Amendments | 4 | If the protocol represents an amendment of a previously completed or published protocol, identify as such and list changes; otherwise, state plan for documenting important protocol amendments |  |
| Support: |  |  |  |
| Sources | 5a | Indicate sources of financial or other support for the review |  |
| Sponsor | 5b | Provide a name for the review funder and/or sponsor |  |
| Role of sponsor or funder | 5c | Describe roles of funder(s), sponsor(s), and/or institution(s), if any, in developing the protocol |  |
| **INTRODUCTION** | | |  |
| Rationale | 6 | Describe the rationale for the review in the context of what is already known |  |
| Objectives | 7 | Provide an explicit statement of the question(s) the review will address concerning participants, interventions, comparators, and outcomes (PICO) |  |
| **METHODS** | | |  |
| Eligibility criteria | 8 | Specify the study characteristics (such as PICO, study design, setting, time frame) and report characteristics (such as years considered, language, publication status) to be used as criteria for eligibility for the review |  |
| Information sources | 9 | Describe all intended information sources (such as electronic databases, contact with study authors, trial registers, or other grey literature sources) with planned dates of coverage |  |
| Search strategy | 10 | The present draft of the search strategy to be used for at least one electronic database, including planned limits, such that it could be repeated |  |
| Study records: |  |  |  |
| Data management | 11a | Describe the mechanism(s) that will be used to manage records and data throughout the review |  |
| Selection process | 11b | State the process that will be used for selecting studies (such as two independent reviewers) through each phase of the review (that is, screening, eligibility, and inclusion in meta-analysis) |  |
| Data collection process | 11c | Describe the planned method of extracting data from reports (such as piloting forms, done independently, in duplicate), and processes for obtaining and confirming data from investigators |  |
| Data items | 12 | List and define all variables for which data will be sought (such as PICO items, funding sources), any pre-planned data assumptions, and simplifications |  |
| Outcomes and prioritization | 13 | List and define all outcomes for which data will be sought, including prioritization of main and additional outcomes, with rationale |  |
| Risk of bias in individual studies | 14 | Describe anticipated methods for assessing the risk of bias of individual studies, including whether this will be done at the outcome or study level, or both; state how this information will be used in data synthesis |  |
| Data synthesis | 15a | Describe criteria under which study data will be quantitatively synthesized |  |
|  | 15b | If data are appropriate for quantitative synthesis, describe planned summary measures, methods of handling data, and methods of combining data from studies, including any planned exploration of consistency (such as I^2^, Kendall’s τ) |  |
|  | 15c | Describe any proposed additional analyses (such as sensitivity or subgroup analyses, meta-regression) |  |
|  | 15d | If quantitative synthesis is not appropriate, describe the type of summary planned |  |
| Meta-bias(es) | 16 | Specify any planned assessment of meta-bias(es) (such as publication bias across studies, selective reporting within studies) |  |
| Confidence in cumulative evidence | 17 | Describe how the strength of the body of evidence will be assessed (such as GRADE) |  |
| **Note:** It is strongly recommended that this checklist be read in conjunction with the PRISMA-P Explanation and Elaboration (cite when available) for important clarification on the items ^[138].^ | | | |

**References:**

1. Aldridge RW, Hayward AC, Hemming S, Possas L, Ferenando G, Garber E, et al. Effectiveness of peer educators on the uptake of mobile X-ray tuberculosis screening at homeless hostels: a cluster randomised controlled trial. BMJ Open. 2015;5(9):e008050.

2. Bello G, Faragher B, Sanudi L, Namakhoma I, Banda H, Malmborg R, et al. The effect of engaging unpaid informal providers on case detection and treatment initiation rates for TB and HIV in rural Malawi (Triage Plus): A cluster randomised health system intervention trial. PloS one. 2017;12(9):e0183312.

3. Chaisson RE, Keruly JC, McAvinue S, Gallant JE, Moore RD. Effects of an incentive and education program on return rates for PPD test reading in patients with HIV infection. J Acquir Immune Defic Syndr Hum Retrovirol. 1996;11(5):455-9.

4. Churchyard GJ, Fielding K, Roux S, Corbett EL, Chaisson RE, De Cock KM, et al. Twelve-monthly versus six-monthly radiological screening for active case-finding of tuberculosis: a randomised controlled trial. Thorax. 2011;66(2):134-9.

5. Durovni B, Saraceni V, Moulton LH, Pacheco AG, Cavalcante SC, King BS, et al. Effect of improved tuberculosis screening and isoniazid preventive therapy on incidence of tuberculosis and death in patients with HIV in clinics in Rio de Janeiro, Brazil: a stepped wedge, cluster-randomised trial. The Lancet Infectious diseases. 2013;13(10):852-8.

6. Ekwueme OE, Omotowo BI, Agwuna KK. Strengthening contact tracing capacity of pulmonary tuberculosis patients in Enugu, southeast Nigeria: a targeted and focused health education intervention study. BMC Public Health. 2014;14:1175.

7. FitzGerald JM, Patrick DM, Strathdee S, Rekart M, Elwood RK, Schecter MT, et al. Use of incentives to increase compliance for TB screening in a population of intravenous drug users. Vancouver Injection Drug Use Study Group. Int J Tuberc Lung Dis. 1999;3(2):153-5.

8. Griffiths C, Sturdy P, Brewin P, Bothamley G, Eldridge S, Martineau A, et al. Educational outreach to promote screening for tuberculosis in primary care: a cluster randomised controlled trial. Lancet (London, England). 2007;369(9572):1528-34.

9. Harstad I, Henriksen AH, Sagvik E. Collaboration between municipal and specialist public health care in tuberculosis screening in Norway. BMC Health Serv Res. 2014;14:238.

10. Malotte CK, Hollingshead JR, Rhodes F. Monetary versus nonmonetary incentives for TB skin test reading among drug users. Am J Prev Med. 1999;16(3):182-8.

11. Sequeira-Aymar E, Cruz A, Serra-Burriel M, di Lollo X, Gonçalves AQ, Camps-Vilà L, et al. Improving the detection of infectious diseases in at-risk migrants with an innovative integrated multi-infection screening digital decision support tool (IS-MiHealth) in primary care: A pilot cluster-randomized controlled trial. Journal of travel medicine. 2021.

12. Shah L, Rojas Peña M, Mori O, Zamudio C, Kaufman JS, Otero L, et al. A pragmatic stepped-wedge cluster randomized trial to evaluate the effectiveness and cost-effectiveness of active case finding for household contacts within a routine tuberculosis program, San Juan de Lurigancho, Lima, Peru. Int J Infect Dis. 2020;100:95-103.

13. Simwaka BN, Theobald S, Willets A, Salaniponi FM, Nkhonjera P, Bello G, et al. Acceptability and effectiveness of the storekeeper-based TB referral system for TB suspects in sub-districts of Lilongwe in Malawi. PloS one. 2012;7(9):e39746.

14. Uwimana J, Zarowsky C, Hausler H, Jackson D. Training community care workers to provide comprehensive TB/HIV/PMTCT integrated care in KwaZulu-Natal: lessons learnt. Trop Med Int Health. 2012;17(4):488-96.

15. Uwimana J, Zarowsky C, Hausler H, Swanevelder S, Tabana H, Jackson D. Community-based intervention to enhance provision of integrated TB-HIV and PMTCT services in South Africa. Int J Tuberc Lung Dis. 2013;17(10 Suppl 1):48-55.

16. Vo LNQ, Forse RJ, Codlin AJ, Vu TN, Le GT, Do GC, et al. A comparative impact evaluation of two human resource models for community-based active tuberculosis case finding in Ho Chi Minh City, Viet Nam. BMC Public Health. 2020;20(1):934.

17. Volkmann T, Okelloh D, Agaya J, Cain K, Ooko B, Malika T, et al. Pilot implementation of a contact tracing intervention for tuberculosis case detection in Kisumu County, Kenya. Public Health Action. 2016;6(4):217-9.

18. Yassi A, Adu PA, Nophale L, Zungu M. Learning from a cluster randomized controlled trial to improve healthcare workers' access to prevention and care for tuberculosis and HIV in Free State, South Africa: the pivotal role of information systems. Global health action. 2016;9:30528.

19. Yellappa V, Battaglioli T, Gurum SK, Narayanan D, Van der Stuyft P. Involving private practitioners in the Indian tuberculosis programme: a randomised trial. Trop Med Int Health. 2018;23(5):570-9.

20. Zaeh S, Kempker R, Stenehjem E, Blumberg HM, Temesgen O, Ofotokun I, et al. Improving tuberculosis screening and isoniazid preventive therapy in an HIV clinic in Addis Ababa, Ethiopia. Int J Tuberc Lung Dis. 2013;17(11):1396-401.

21. Alisjahbana B, van Crevel R, Danusantoso H, Gartinah T, Soemantri ES, Nelwan RH, et al. Better patient instruction for sputum sampling can improve microscopic tuberculosis diagnosis. Int J Tuberc Lung Dis. 2005;9(7):814-7.

22. Bai LQ, Yang HL, Jian XW, He XG, Chen YF, Tang Y, et al. Increasing tuberculosis case detection through intensive referral and tracing in Hunan, China. Int J Tuberc Lung Dis. 2008;12(12):1431-5.

23. Balakrishnan S, Ps R, M S, Sankar B, Ramachandran R, Ka A, et al. STEPS: A Solution for Ensuring Standards of TB Care for Patients Reaching Private Hospitals in India. Glob Health Sci Pract. 2021;9(2):286-95.

24. Becx-Bleumink M, Wibowo H, W. Apriani, Vrakking H. High tuberculosis notification and treatment success rates through community participation in central sulawesi, Republic of Indonesia. International Journal of Tuberculosis and Lung Disease. 2001;5(10):920-5.

25. Bjerrum S, Bonsu F, Hanson-Nortey NN, Kenu E, Johansen IS, Andersen AB, et al. Tuberculosis screening in patients with HIV: use of audit and feedback to improve quality of care in Ghana. Global health action. 2016;9:32390.

26. Calligaro GL, Zijenah LS, Peter JG, Theron G, Buser V, McNerney R, et al. Effect of new tuberculosis diagnostic technologies on community-based intensified case finding: a multicentre randomised controlled trial. Lancet Infectious Diseases. 2017;(no pagination).

27. Corbett EL, Bandason T, Duong T, Dauya E, Makamure B, Churchyard GJ, et al. Comparison of two active case-finding strategies for community-based diagnosis of symptomatic smear-positive tuberculosis and control of infectious tuberculosis in Harare, Zimbabwe (DETECTB): a cluster-randomised trial. Lancet (London, England). 2010;376(9748):1244-53.

28. Datiko DG, Lindtjorn B. Health extension workers improve tuberculosis case detection and treatment success in southern Ethiopia: a community randomized trial. PLoS One 2009;4(5):e5443.

29. Dudley L, Azevedo V, Grant R, Schoeman JH, Dikweni L, Maher D. Evaluation of community contribution to tuberculosis control in Cape Town, South Africa. International Journal of Tuberculosis and Lung Disease. 2003;7(9):548-55.

30. Durovni B, Saraceni V, van den Hof S, Trajman A, Cordeiro-Santos M, Cavalcante S, et al. Impact of replacing smear microscopy with Xpert MTB/RIF for diagnosing tuberculosis in Brazil: a stepped-wedge cluster-randomized trial. PLoS Medicine 2014;11(12):e1001766.

31. Eom JS, Park S, Jang H, Kim S, Yoo WH, Kim SH, et al. Bronchial washing using a thin versus a thick bronchoscope to diagnose pulmonary tuberculosis: A randomized trial. Clinical infectious diseases : an official publication of the Infectious Diseases Society of America. 2022.

32. Fairall LR, Mayers P. Erratum: Effect of educational outreach to nurses on tuberculosis case detection and primary care of respiratory illness: Pragmatic cluster randomised controlled trial (British Medical Journal (October 1, 2005) 331 (750-754)). Br Med J. 2005;331(7525):1120.

33. Garg T, Bhardwaj M, Deo S, bmjopen. Role of community health workers in improving cost efficiency in an active case finding tuberculosis programme: an operational research study from rural Bihar, India. BMJ Open. 2020;10(10):e036625.

34. Geldenhuys HD, Whitelaw A, Tameris MD, Van As D, Luabeya KK, Mahomed H, et al. A controlled trial of sputum induction and routine collection methods for TB diagnosis in a South African community. Eur J Clin Microbiol Infect Dis. 2014;33(12):2259-66.

35. Gengiah S, Barker PM, Yende-Zuma N, Mbatha M, Naidoo S, Taylor M, et al. A cluster-randomized controlled trial to improve the quality of integrated HIV-tuberculosis services in primary healthcareclinics in South Africa. J Int AIDS Soc. 2021;24(9):e25803.

36. Kaswaswa K, MacPherson P, Kumwenda M, Mpunga J, Thindwa D, Nliwasa M, et al. Effect of patient-delivered household contact tracing and prevention for tuberculosis: A household cluster-randomised trial in Malawi. PloS one. 2022;17(9):e0269219.

37. Khan MS, Dar O, Sismanidis C, Shah K, Godfrey-Faussett P. Improvement of tuberculosis case detection and reduction of discrepancies between men and women by simple sputum-submission instructions: a pragmatic randomised controlled trial. Lancet (London, England). 2007;369(9577):1955-60.

38. Khan MA, Anil S, Ahmed M, Athar A, Ghafoor A, Brouwer M. Active Case Finding of Tuberculosis: Randomized Evaluation of Simple and Infotainment Chest Camps. Ann Glob Health. 2016;82(5):813-8.

39. Khan MA, Munir MA, Anil S, Ahmad M, Walley J, Qadeer E, et al. Structured performance monitoring of TB-care at facility, district and province levels - Pakistan experience. J Pak Med Assoc. 2016;66(4):418-24.

40. Lisboa M, Fronteira I, Mason PH, Martins M. Using hospital auxiliary worker and 24-h TB services as potential tools to overcome in-hospital TB delays: a quasi-experimental study. Hum Resour Health. 2020;18(1):28.

41. MacPherson P, Webb EL, Kamchedzera W, Joekes E, Mjoli G, Lalloo DG, et al. Computer-aided X-ray screening for tuberculosis and HIV testing among adults with cough in Malawi (the PROSPECT study): A randomised trial and cost-effectiveness analysis. PLoS medicine. 2021;18(9):e1003752.

42. Martinson NA, Lebina L, Webb EL, Ratsela A, Varavia E, Kinghorn A, et al. Household Contact Tracing With Intensified Tuberculosis and Human Immunodeficiency Virus Screening in South Africa: A Cluster-Randomized Trial. Clinical infectious diseases : an official publication of the Infectious Diseases Society of America. 2022;75(5):849-56.

43. Mhalu G, Hella J, Doulla B, Mhimbira F, Mtutu H, Hiza H, et al. Do Instructional Videos on Sputum Submission Result in Increased Tuberculosis Case Detection? A Randomized Controlled Trial. PloS one. 2015;10(9):e0138413.

44. Parija D, Patra TK, Kumar AM, Swain BK, Satyanarayana S, Sreenivas A, et al. Impact of awareness drives and community-based active tuberculosis case finding in Odisha, India. Int J Tuberc Lung Dis. 2014;18(9):1105-7.

45. Qureshi H, Arif A, Alam E, Qadir N. Integration of informal medical practitioners in DOTS implementation to improve case detection rate. J Pak Med Assoc. 2010;60(1):33-7.

46. Rudolf F, Wejse C, S X. Tuberculosis case detection revisited: better testing might not improve outcomes. The Lancet Global Health. 2015;3(8):e424‐e5.

47. Ruutel K, Loit HM, Sepp T, Kliiman K, McNutt LA, Uuskula A. Enhanced tuberculosis case detection among substitution treatment patients: a randomized controlled trial. BMC Res Notes. 2011;4:192.

48. Sah R, Singh UK, Mainali R, Sanaie A, Pande T, Vasquez NA, et al. Engaging Private Health Care Providers to Identify Individuals with TB in Nepal. Int J Environ Res Public Health. 2021;18(22).

49. Thu TD, Kumar AMV, Ramaswamy G, Htun T, Van HL, Quang LVN, et al. An Innovative Public-Private Mix Model for Improving Tuberculosis Care in Vietnam: How Well are We Doing? Tropical medicine and infectious disease. 2020;5(1).

50. Timire C, Takarinda KC, Sandy C, Zishiri C, Kumar AMV, Harries AD. Has TB CARE I sputum transport improved access to culture services for retreatment tuberculosis patients in Zimbabwe? Public Health Action. 2018;8(2):66-71.

51. Wang L, Cheng S, Xu M, Huang F, Xu W, Li R, et al. Model collaboration between hospitals and public health system to improve tuberculosis control in China. Int J Tuberc Lung Dis. 2009;13(12):1486-92.

52. Wei X, Zou G, Chong MK, Xu L. An intervention of active TB case finding among smokers attending routine primary care facilities in China: an exploratory study. Transactions of the Royal Society of Tropical Medicine and Hygiene. 2015;109(9):545-52.

53. Yassin MA, Datiko DG, Tulloch O, Markos P, Aschalew M, Shargie EB, et al. Innovative community-based approaches doubled tuberculosis case notification and improve treatment outcome in Southern Ethiopia. PloS one. 2013;8(5):e63174.

54. Adane K, Spigt M, Winkens B, Dinant GJ, s x. Tuberculosis case detection by trained inmate peer educators in a resource-limited prison setting in Ethiopia: a cluster-randomised trial. The Lancet Global health. 2019;7(4):e482-e91.

55. Al-Sahafi A, Al-Sayali MM, Mandoura N, Shah HBU, Al Sharif K, Almohammadi EL, et al. Treatment outcomes among tuberculosis patients in Jeddah, Saudi Arabia: Results of a community mobile outreach directly observed Treatment, Short-course (DOTS) project, compared to a standard facility-based DOTS: A randomized controlled trial. Journal of clinical tuberculosis and other mycobacterial diseases. 2021;22:100210.

56. Bassett IV, Coleman SM, Giddy J, Bogart LM, Chaisson CE, Ross D, et al. Sizanani: A Randomized Trial of Health System Navigators to Improve Linkage to HIV and TB Care in South Africa. Journal of acquired immune deficiency syndromes (1999). 2016;73(2):154-60.

57. Jenum S, Selvam S, Jesuraj N, Ritz C, Hesseling AC, Cardenas V, et al. Incidence of tuberculosis and the influence of surveillance strategy on tuberculosis case-finding and all-cause mortality: a cluster randomised trial in Indian neonates vaccinated with BCG. BMJ Open Respir Res. 2018;5(1):e000304.

58. Lee JE, Kim YK, Kim TH, Kim KH, Lee EJ, Uh ST, et al. What strategy can be applied to the patients with culture positive tuberculosis to reduce treatment delay in a private tertiary healthcare center? Infection and Chemotherapy. 2011;43(1):42-7.

59. Majella M, Thekkur P, Kumar A, Chinnakali P, Saka V, Roy G. Effect of mobile voice calls on treatment initiation among patients diagnosed with tuberculosis in a tertiary care hospital of Puducherry: A randomized controlled trial. Journal of Postgraduate Medicine. 2021;67(4):205-12.

60. Mohan A, Nassir H, Niazi A. Does routine home visiting improve the return rate and outcome of DOTS patients who delay treatment? Eastern Mediterranean health journal = La revue de sante de la Mediterranee orientale = al-Majallah al-sihhiyah li-sharq al-mutawassit. 2003;9(4):702-8.

61. Mukoka M, Twabi HH, Msefula C, Semphere R, Ndhlovu G, Lipenga T, et al. Utility of Xpert MTB/RIF Ultra and digital chest radiography for the diagnosis and treatment of TB in people living with HIV: a randomised controlled trial (XACT-TB). Transactions of the Royal Society of Tropical Medicine and Hygiene. 2022.

62. Mwansa-Kambafwile JRM, Chasela C, Levin J, Ismail N, Menezes C. Treatment initiation among tuberculosis patients: the role of short message service (SMS) technology and Ward-based outreach teams (WBOTs). BMC Public Health. 2022;22(1):318.

63. Wagstaff A, van Doorslaer E, Burger R, journal.pone. SMS nudges as a tool to reduce tuberculosis treatment delay and pretreatment loss to follow-up. A randomized controlled trial. PloS one. 2019;14(6):e0218527.

64. White MC, Tulsky JP, Reilly P, McIntosh HW, Hoynes TM, Goldenson J. A clinical trial of a financial incentive to go to the tuberculosis clinic for isoniazid after release from jail. Int J Tuberc Lung Dis. 1998;2(6):506-12.

65. Wingfield T, Tovar MA, Huff D, Boccia D, Montoya R, Ramos E, et al. A randomized controlled study of socioeconomic support to enhance tuberculosis prevention and treatment, Peru. Bulletin of the World Health Organization. 2017;95(4):270-80.

66. Ali AOA, Prins MH. Mobile health to improve adherence to tuberculosis treatment in Khartoum state, Sudan. Journal of public health in Africa. 2019;10(2):1101.

67. Müller AM, Osório CS, de Figueiredo RV, Silva DR, Dalcin PTR, crj. Educational strategy intervention and remote supervision on the post-discharge management of tuberculosis diagnosed in the hospital: Randomized clinical trial. Clin Respir J. 2019;13(8):505-12.

68. Al-Sayali MM, Mandoura N, Shah HBU, Al Sharif K, Almohammadi EL, Abdul-Rashid OA, et al. Treatment outcomes among tuberculosis patients in Jeddah, Saudi Arabia: Results of a community mobile outreach directly observed Treatment, Short-course (DOTS) project, compared to a standard facility-based DOTS: A randomized controlled trial. Journal of clinical tuberculosis and other mycobacterial diseases. 2021;22.

69. Awaisu A, Nik Mohamed MH, Mohamad Noordin N, Abd Aziz N, Syed Sulaiman SA, Muttalif AR, et al. The SCIDOTS Project: evidence of benefits of an integrated tobacco cessation intervention in tuberculosis care on treatment outcomes. Subst Abuse Treat Prev Policy. 2011;6:26.

70. Baluku JB, Nakazibwe B, Twinomugisha B, Najjuuko R, Isabella N, Nassozi S, et al. One dollar incentive improves tuberculosis treatment outcomes in programmatic settings in rural Uganda. Sci Rep. 2021;11(1):19346.

71. Bediang G, Stoll B, Elia N, Abena JL, Geissbuhler A. SMS reminders to improve adherence and cure of tuberculosis patients in Cameroon (TB-SMS Cameroon): a randomised controlled trial. BMC Public Health. 2018;18(1):583.

72. Broomhead S, Mars M. Retrospective return on investment analysis of an electronic treatment adherence device piloted in the Northern Cape Province. Telemedicine journal and e-health : the official journal of the American Telemedicine Association. 2012;18(1):24-31.

73. Datiko DG, Yassin MA, Theobald SJ, Blok L, Suvanand S, Creswell J, et al. Health extension workers improve tuberculosis case finding and treatment outcome in Ethiopia: a large-scale implementation study. BMJ Glob Health. 2017;2(4):e000390.

74. Diaw MM, Ndiaye M, Riccardi N, Ungaro R, Alagna R, Cirillo DM, et al. Implementing TB control in a rural, resource-limited setting: the stop-TB Italia project in Senegal. Multidiscip Respir Med. 2018;13:41.

75. Farooqi RJ, Ashraf S, Zaman M. The role of mobile SMS-reminders in improving drugs compliance in patients receiving anti-TB treatment from DOTS program. Journal of Postgraduate Medical Institute. 2017;31(2):156-62.

76. Fatima R, Yaqoob A, Qadeer E, Khan MA, Ghafoor A, Jamil B, et al. Community- vs. hospital-based management of multidrug-resistant TB in Pakistan. Int J Tuberc Lung Dis. 2022;26(10):929-33.

77. Janmeja AK, Das SK, Bhargava R, Chavan BS. Psychotherapy improves compliance with tuberculosis treatment. Respiration; international review of thoracic diseases. 2005;72(4):375-80.

78. John S, Gidado M, Dahiru T, Fanning A, Codlin AJ, Creswell J. Tuberculosis among nomads in Adamawa, Nigeria: outcomes from two years of active case finding. Int J Tuberc Lung Dis. 2015;19(4):463-8.

79. Khan MS, Suwannapong N, Howteerakul N, Pacheun O, Rajatanun T. Improvement of district hospital service system to increase treatment adherence among tuberculosis patients in Pakistan. The Southeast Asian journal of tropical medicine and public health. 2011;42(3):664-73.

80. Khortwong P, Kaewkungwal J. Thai health education program for improving TB migrant's compliance. Journal of the Medical Association of Thailand = Chotmaihet thangphaet. 2013;96(3):365-73.

81. Kunawararak P, Pongpanich S, Chantawong S, Pokaew P, Traisathit P, Srithanaviboonchai K, et al. Tuberculosis Treatment With Mobile-Phone Medication Reminders in Northern Thailand. The Southeast Asian journal of tropical medicine and public health. 2011;42(6):1444-51.

82. Lee CY, Chi MJ, Yang SL, Lo HY, Cheng SH. Using financial incentives to improve the care of tuberculosis patients. Am J Manag Care. 2015;21(1):e35-42.

83. Lewin S, Dick J, Zwarenstein M, Lombard CJ. Staff training and ambulatory tuberculosis treatment outcomes: a cluster randomized controlled trial in South Africa. Bull World Health Organ. 2005;83(4):250-9.

84. Louwagie G, Kanaan M, Morojele NK, Van Zyl A, Moriarty AS, Li J, et al. Effect of a brief motivational interview and text message intervention targeting tobacco smoking, alcohol use and medication adherence to improve tuberculosis treatment outcomes in adult patients with tuberculosis: a multicentre, randomised controlled trial of the ProLife programme in South Africa. BMJ Open. 2022;12(2):e056496.

85. Lutge E, Lewin S, Volmink J, Friedman I, Lombard C. Economic support to improve tuberculosis treatment outcomes in South Africa: a pragmatic cluster-randomized controlled trial. Trials. 2013;14:154.

86. Manyazewal T, Woldeamanuel Y, Holland DP, Fekadu A, Marconi VC. Effectiveness of a digital medication event reminder and monitor device for patients with tuberculosis (SELFTB): a multicenter randomized controlled trial. BMC Med. 2022;20(1):310.

87. Miti S, Mfungwe V, Reijer P, Maher D. Integration of tuberculosis treatment in a community-based home care programme for persons living with HIV/AIDS in Ndola, Zambia. International Journal of Tuberculosis and Lung Disease. 2003;7(9):S92-S8.

88. Khan AJ, Khowaja S, Khan FS, Qazi F, Lotia I, Habib A, et al. Engaging the private sector to increase tuberculosis case detection: an impact evaluation study. The Lancet Infectious diseases. 2012;12(8):608-16.

89. Niazi AD, Al-Delaimi AM. Impact of community participation on treatment outcomes and compliance of DOTS patients in Iraq. Eastern Mediterranean health journal = La revue de sante de la Mediterranee orientale = al-Majallah al-sihhiyah li-sharq al-mutawassit. 2003;9(4):709-17.

90. Peltzer K, Naidoo P, Louw J, Matseke G, Zuma K, McHunu G, et al. Screening and brief interventions for hazardous and harmful alcohol use among patients with active tuberculosis attending primary public care clinics in South Africa: results from a cluster randomized controlled trial. BMC Public Health. 2013;13:699.

91. Puchalski Ritchie LM, Schull MJ, Martiniuk AL, Barnsley J, Arenovich T, van Lettow M, et al. A knowledge translation intervention to improve tuberculosis care and outcomes in Malawi: a pragmatic cluster randomized controlled trial. Implementation science : IS. 2015;10:38.

92. Taneja N, Chellaiyan VG, Daral S, Adhikary M, Das TK. Home Based Care as an Approach to Improve the Efficiency of treatment for MDR Tuberculosis: A Quasi-Experimental Pilot Study. Journal of clinical and diagnostic research : JCDR. 2017;11(8):Lc05-lc8.

93. Thekkur P, Kumar AN, Chinnakali P, Selvaraju S, Bairy R, Singh AR, et al. Outcomes and implementation challenges of using daily treatment regimens with an innovative adherence support tool among HIV-infected tuberculosis patients in Karnataka, India: a mixed-methods study. Global health action. 2019;12(1):1568826.

94. Thiam S, LeFevre AM, Hane F, Ndiaye A, Ba F, Fielding KL, et al. Effectiveness of a strategy to improve adherence to tuberculosis treatment in a resource-poor setting: a cluster randomized controlled trial. Jama. 2007;297(4):380-6.

95. Torrens AW, Rasella D, Boccia D, Maciel EL, Nery JS, Olson ZD, et al. Effectiveness of a conditional cash transfer programme on TB cure rate: a retrospective cohort study in Brazil. Transactions of the Royal Society of Tropical Medicine and Hygiene. 2016;110(3):199-206.

96. Venkatapraveen A, Rampure MV, Patil N, Shivanand Hinchageri SS, Lakshmi DP. Assessment of clinical pharmacist intervention to improve compliance and health care outcomes of tuberculosis patients. Der Pharmacia Lettre. 2012;4(3):931-7.

97. Wongduan S, Wilawan P, Wanchai L, Akeau U. A Medication Adherence Enhancement Program for Persons with Pulmonary Tuberculosis: A Randomized Controlled Trial Study. Pacific Rim International Journal of Nursing Research. 2015;19(4):311-29.

98. Burzynski J, Mangan JM, Lam CK, Macaraig M, Salerno MM, deCastro BR, et al. In-Person vs Electronic Directly Observed Therapy for Tuberculosis Treatment Adherence: A Randomized Noninferiority Trial. JAMA Netw Open. 2022;5(1):e2144210.

99. Chaisson RE, Barnes GL, Hackman J, Watkinson L, Kimbrough L, Metha S, et al. A randomized, controlled trial of interventions to improve adherence to isoniazid therapy to prevent tuberculosis in injection drug users. Am J Med. 2001;110(8):610-5.

100. Chua AP, Lim LK, Ng H, Chee CB, Wang YT. Outcome of a grocery voucher incentive scheme for low-income tuberculosis patients on directly observed therapy in Singapore. Singapore medical journal. 2015;56(5):274-9.

101. Chuck C, Robinson E, Macaraig M, Alexander M, Burzynski J. Enhancing management of tuberculosis treatment with video directly observed therapy in New York City. Int J Tuberc Lung Dis. 2016;20(5):588-93.

102. Demissie M, Getahun H, Lindtjørn B. Community tuberculosis care through “TB clubs” in rural North Ethiopia. Social Science & Medicine. 2003;56(10):2009-18.

103. Fang XH, Guan SY, Tang L, Tao FB, Zou Z, Wang JX, et al. Effect of Short Message Service on Management of Pulmonary Tuberculosis Patients in Anhui Province, China: A Prospective, Randomized, Controlled Study. Med Sci Monit. 2017;23:2465-9.

104. Guo P, Qiao W, Sun Y, Liu F, Wang C, tmj. Telemedicine Technologies and Tuberculosis Management: A Randomized Controlled Trial. Telemedicine journal and e-health : the official journal of the American Telemedicine Association. 2020;26(9):1150-6.

105. Hermans SM, Elbireer S, Tibakabikoba H, Hoefman BJ, Manabe YC. Text messaging to decrease tuberculosis treatment attrition in TB-HIV coinfection in Uganda. Patient Preference and Adherence 2017;11:1479-87.

106. Hsieh CJ, Lin LC, Kuo BI, Chiang CH, Su WJ, Shih JF. Exploring the efficacy of a case management model using DOTS in the adherence of patients with pulmonary tuberculosis. Journal of clinical nursing. 2008;17(7):869-75.

107. Jahnavi G, Sudha CH. Randomised controlled trial of food supplements in patients with newly diagnosed tuberculosis and wasting. Singapore medical journal. 2010;51(12):957-62.

108. Kufa T, Fielding KL, Hippner P, Kielmann K, Vassall A, Churchyard GJ, et al. An intervention to optimise the delivery of integrated tuberculosis and HIV services at primary care clinics: results of the MERGE cluster randomised trial. Contemporary clinical trials. 2018;72:43-52.

109. Liefooghe R, Suetens C, Meulemans H, Moran MB, De Muynck A. A randomised trial of the impact of counselling on treatment adherence of tuberculosis patients in Sialkot, Pakistan. Int J Tuberc Lung Dis. 1999;3(12):1073-80.

110. Martins N, Morris P, Kelly PM. Food incentives to improve completion of tuberculosis treatment: randomised controlled trial in Dili, Timor-Leste. BMJ. 2009;339(7730):b4248-b.

111. Miller AC, Golub JE, Cavalcante SC, Durovni B, Moulton LH, Fonseca Z, et al. Controlled trial of active tuberculosis case finding in a Brazilian favela. Int J Tuberc Lung Dis. 2010;14(6):720-6.

112. Mohammed S, Glennerster R, Khan AJ. Impact of a Daily SMS Medication Reminder System on Tuberculosis Treatment Outcomes: A Randomized Controlled Trial. PloS one. 2016;11(11):e0162944.

113. Morisky DE, Malotte CK, Ebin V, Davidson P, Cabrera D, Trout PT, et al. Behavioral interventions for the control of tuberculosis among adolescents. Public Health Rep. 2001;116(6):568-74.

114. Moulding TS, Caymittes M. Managing medication compliance of tuberculosis patients in Haiti with medication monitors. International Journal of Tuberculosis and Lung Disease. 2002;6(4):313‐9.

115. Nyamathi AM, Christiani A, Nahid P, Gregerson P, Leake B. A randomized controlled trial of two treatment programs for homeless adults with latent tuberculosis infection. Int J Tuberc Lung Dis. 2006;10(7):775-82.

116. Plokhykh V, Duka M, Cassidy L, Chen CY, Malakyan K, Isaakidis P, et al. Mental health interventions for rifampicin-resistant tuberculosis patients with alcohol use disorders, Zhytomyr, Ukraine. J Infect Dev Ctries. 2021;15(9.1):25s-33s.

117. Rajasekaran S. Short course chemotherapy: A controlled study of indirect defaulter retrieval method. The Indian journal of tuberculosis. 1993;40.

118. Rocha C, Montoya R, Zevallos K, Curatola A, Ynga W, Franco J, et al. The Innovative Socio-economic Interventions Against Tuberculosis (ISIAT) project: an operational assessment. Int J Tuberc Lung Dis. 2011;15 Suppl 2:50-7.

119. Turnbull L, Bell C, Davies S, Child F. Delivering tertiary tuberculosis care virtually. Arch Dis Child. 2021;106(12):1226-8.

120. Wei X, Zou G, Yin J, Walley J, Yang H, Kliner M, et al. Providing financial incentives to rural-to-urban tuberculosis migrants in Shanghai: an intervention study. Infect Dis Poverty. 2012;1(1):9.

121. Yao H, Wei X, Liu J, Zhao J, Hu D, Walley JD. Evaluating the effects of providing financial incentives to tuberculosis patients and health providers in China. Int J Tuberc Lung Dis. 2008;12(10):1166-72.

122. Zou G, Wei X, Witter S, Yin J, Walley J, Liu S, et al. Incremental cost-effectiveness of improving treatment results among migrant tuberculosis patients in Shanghai. Int J Tuberc Lung Dis. 2013;17(8):1056-64.

123. Acosta J, Flores P, Alarcón M, Grande-Ortiz M, Moreno-Exebio L, Puyen ZM. A randomised controlled trial to evaluate a medication monitoring system for TB treatment. Int J Tuberc Lung Dis. 2022;26(1):44-9.

124. Cattamanchi A, Crowder R, Kityamuwesi A, Kiwanuka N, Lamunu M, Namale C, et al. Digital adherence technology for tuberculosis treatment supervision: A stepped-wedge cluster-randomized trial in Uganda. PLoS medicine. 2021;18(5):1-15.

125. Gashu KD, Gelaye KA, Lester R, Tilahun B. Effect of a phone reminder system on patient-centered tuberculosis treatment adherence among adults in Northwest Ethiopia: a randomised controlled trial. BMJ Health Care Inform. 2021;28(1).

126. Iribarren S, Beck S, Pearce PF, Chirico C, Etchevarria M, Cardinale D, et al. TextTB: A Mixed Method Pilot Study Evaluating Acceptance, Feasibility, and Exploring Initial Efficacy of a Text Messaging Intervention to Support TB Treatment Adherence. Tuberc Res Treat. 2013;2013:349394.

127. Lee S, Khan OF, Seo JH, Kim DY, Park KH, Jung SI, et al. Impact of Physician's Education on Adherence to Tuberculosis Treatment for Patients of Low Socioeconomic Status in Bangladesh. Chonnam medical journal. 2013;49(1):27-30.

128. Lu H, Yan F, Wang W, Wu L, Ma W, Chen J, et al. Do transportation subsidies and living allowances improve tuberculosis control outcomes among internal migrants in urban Shanghai, China? Western Pac Surveill Response J. 2013;4(1):19-24.

129. Parwati NM, Bakta IM, Januraga PP, Wirawan IMA. A Health Belief Model-Based Motivational Interviewing for Medication Adherence and Treatment Success in Pulmonary Tuberculosis Patients. Int J Environ Res Public Health. 2021;18(24).

130. Ravenscroft L, Kettle S, Persian R, Ruda S, Severin L, Doltu S, et al. Video-observed therapy and medication adherence for tuberculosis patients: randomised controlled trial in Moldova. Eur Respir J. 2020;56(2).

131. Shargie EB, Morkve O, Lindtjorn B. Tuberculosis case-finding through a village outreach programme in a rural setting in southern Ethiopia: community randomized trial. Bull World Health Organ. 2006;84(2):112-9.

132. Shin S, Livchits V, Connery HS, Shields A, Yanov S, Yanova G, et al. Effectiveness of alcohol treatment interventions integrated into routine tuberculosis care in Tomsk, Russia. Addiction. 2013;108(8):1387-96.

133. Soares EC, Vollmer WM, Cavalcante SC, Pacheco AG, Saraceni V, Silva JS, et al. Tuberculosis control in a socially vulnerable area: a community intervention beyond DOT in a Brazilian favela. Int J Tuberc Lung Dis. 2013;17(12):1581-6.

134. Sudarsanam TD, John J, Kang G, Mahendri V, Gerrior J, Franciosa M, et al. Pilot randomized trial of nutritional supplementation in patients with tuberculosis and HIV-tuberculosis coinfection receiving directly observed short-course chemotherapy for tuberculosis. Trop Med Int Health. 2011;16(6):699-706.

135. Thomas B, Watson B, Senthil EK, Deepalakshmi A, Balaji G, Chandra S, et al. Alcohol intervention strategy among tuberculosis patients: a pilot study from South India. Int J Tuberc Lung Dis. 2017;21(8):947-52.

136. Ukwaja KN, Alobu I, Gidado M, Onazi O, Oshi DC, ijtld. Economic support intervention improves tuberculosis treatment outcomes in rural Nigeria. International Journal of Tuberculosis and Lung Disease. 2017;21(5):564‐70.

137. Armijo-Olivo S, Stiles CR, Hagen NA, Biondo PD, Cummings GG. Assessment of study quality for systematic reviews: a comparison of the Cochrane Collaboration Risk of Bias Tool and the Effective Public Health Practice Project Quality Assessment Tool: methodological research. Journal of Evaluation in Clinical Practice 2012;18(1):12-8.

138. Shamseer L, Moher D, Clarke M, Ghersi D, Liberati A, Petticrew M, et al. Preferred reporting items for systematic review and meta-analysis protocols (PRISMA-P) 2015: elaboration and explanation. Bmj. 2015;350:g7647.
