# Supplementary figures and images for: Improving cascade outcomes for active TB: A global systematic review and meta-analysis of TB interventions
Source: PLoS Med. 2023 Jan 3;20(1):e1004091. doi: 10.1371/journal.pmed.1004091 (PMC9847969; doi:10.1371/journal.pmed.1004091)

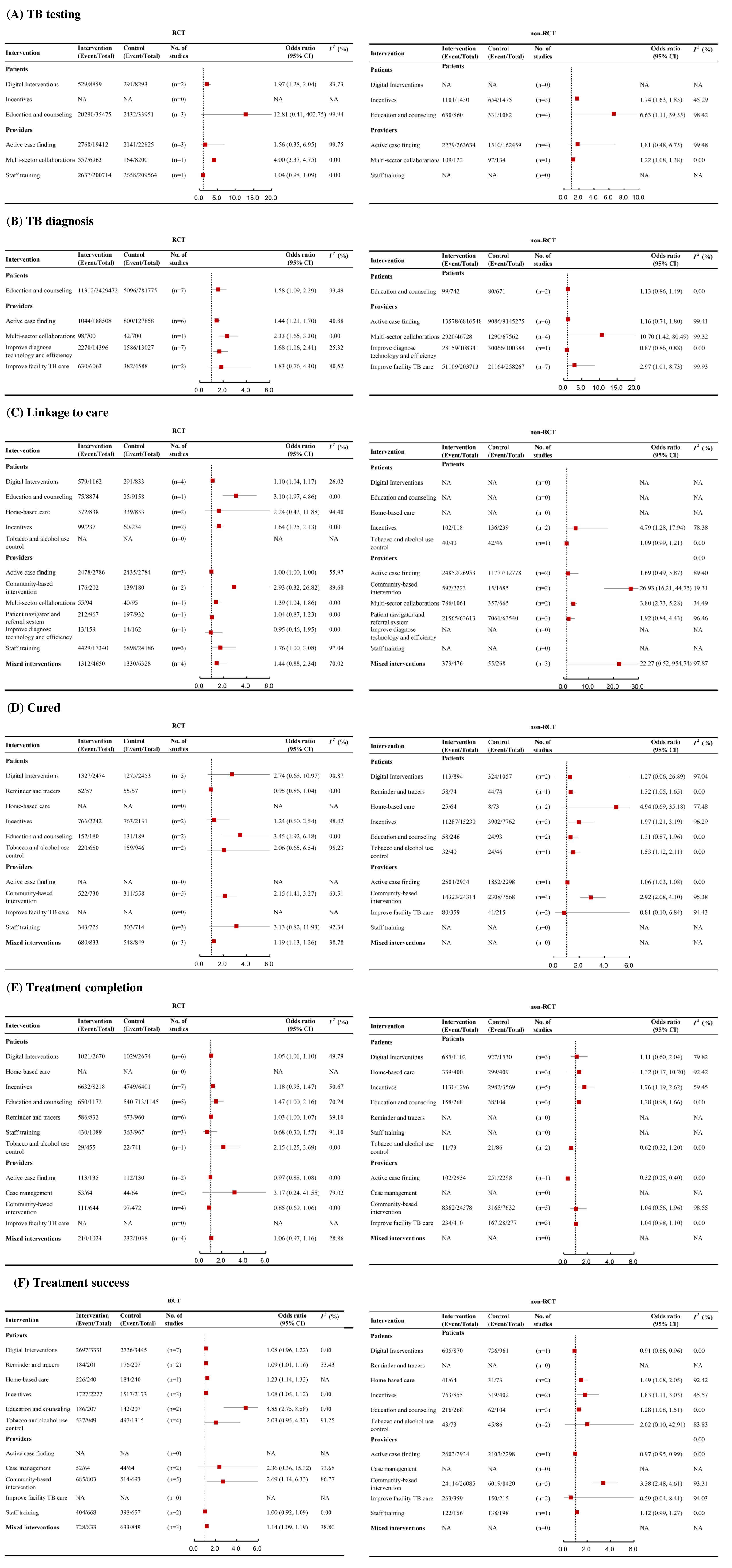

Supplement: S2 Fig — (TIFF) [file pmed.1004091.s003.tiff]

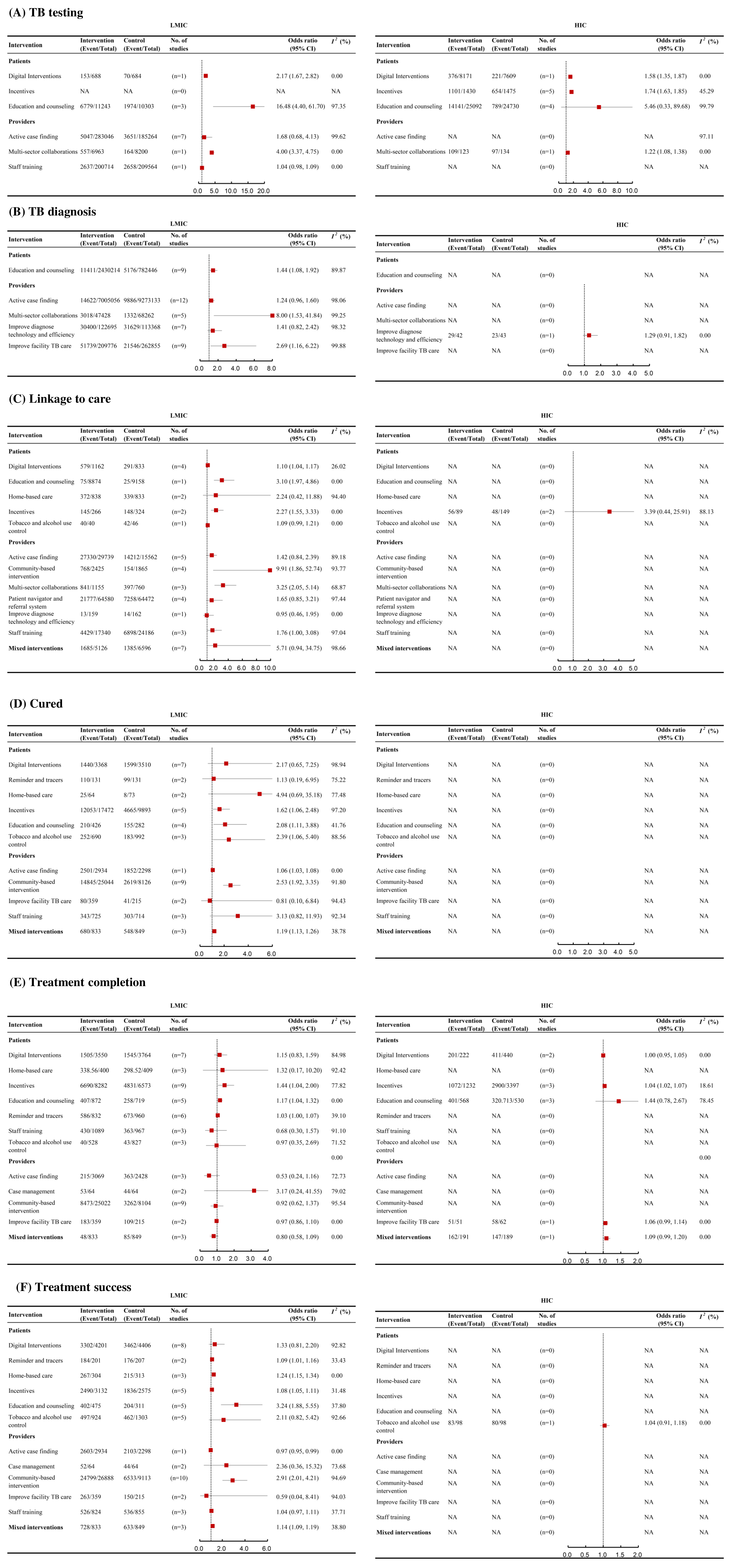

Supplement: S3 Fig — (TIFF) [file pmed.1004091.s004.tiff]

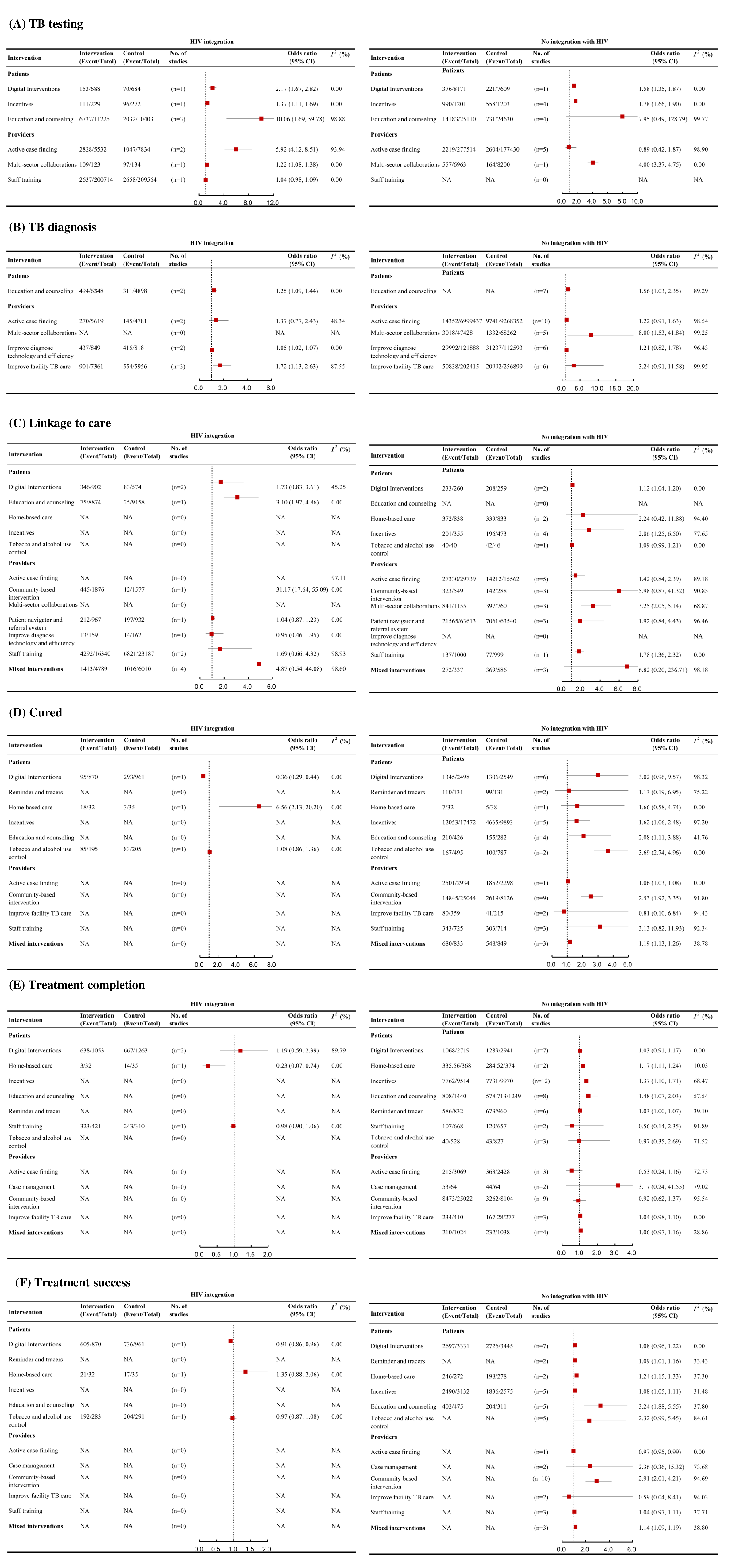

Supplement: S4 Fig — (TIFF) [file pmed.1004091.s005.tiff]
