# Supplementary material for: Improving cascade outcomes for active TB: A global systematic review and meta-analysis of TB interventions
Source: PLoS Med. 2023 Jan 3;20(1):e1004091. doi: 10.1371/journal.pmed.1004091 (PMC9847969; doi:10.1371/journal.pmed.1004091)
Supplement: Attachment — Submitted filename: marley.pdf [file pmed.1004091.s008.pdf]

This paper reports a systematic review with meta–analysis of interventions in the care of active tuberculosis.

I was asked for a statistical report and I interpret that to include all aspects of the design and conduct of the study.

Points of detail

Page 6 The language restriction mentioned on page 3 in the abstract should also be mentioned here. More importantly why was such a restriction imposed? If a high proportion of studies were expected to be in low– and middle–income countries then it seems too restrictive. For a condition which has a major impact in resource poor countries bibliographic databases with a coverage of the literature of those countries would seem indicated. Various empirical studies have indicated that the main bibliographic databases have varied and incomplete coverage of material not in English (Pilkington et al., 2005; Shenderovich et al., 2016), and have limited coverage of material from low and middle–income country journals (Kieling et al., 2009; Syed Sheriff et al., 2008). A wider selection of data–bases and inclusion of material other than in English would seem valuable. The authors might wish to read Jackson et al. (2019) for a brief report on using machine translation in systematic reviewing.

Page 6 Why exclude dissertations?

Page 6 I can see that previous systematic reviews are not suitable for the meta–analysis but they are presumably a source of studies which might otherwise be missed. Similarly did the authors use the reference lists of the studies they did find to identify possible other studies? Figure refers to hand searching but without clear explanation of what that means.

Page 7 I think there is an ambiguity possible here. '... without evidence of failure or a record to show a sputum smear or ...'. Does this mean '... without (evidence of failure) or (a record to show a sputum smear or ...)' '... without evidence of (failure) or (a record to show a sputum smear or ...)'?

Page 8 I do not see what 'similar comparator arms and intervention strategies' means. I thought the whole point of the authors' review was to include a wide range of comparators and strategies. It would be helpful to clarify what comparators were included. Are these all trials against

treatment as usual or are some head–to–head? If the latter then network meta–analysis would help to compare different treatment options.

Page 8 The phrase publication bias implies you know the aetiology. There are other ways in which small studies may differ and some of these like case–mix might well be an issue here.

Page 10 Having said that the small numbers preclude looking at small sample effects the authors then go on to present the results of Egger's test on some very small numbers. This seems flatly contradictory.

Page 15 Here the authors state 'digital interventions was not significantly associated with any' but on page 10 they report 'digital interventions [...] were each associated with increased likelihood of testing.' This seems flatly contradictory.

Supplementary Figure 2 On page 10 the authors state that all of the plots show evidence of asymmetry. All of the sub–tables would benefit from telling us the value of the statistic from Egger's method.

2.1 The y–axis means everything is compressed at the top. I see no evidence of systematic small study effects here contrary to the statement on page 10. There is an outlying value.

2.2 This one is more worrying I agree.

2.3 The y–axis could use adjustment and there are a couple of outliers but little evidence of systematic small study effects.

2.4 Remember that Egger's method is a regression. The green square bottom right will be probably be having a massive influence on the fitted model.

2.5 Again y–axis and again little to worry about here.

2.6 It is hard to see what is going on here because the three types of plot points overlap one another so we cannot see where, if anywhere, things are going wrong.

Supplementary Table 6 Contrary to the caption this does not show regional differences.

Points of more substance

Detail of reporting

The PRISMA guidelines ask (requirement 18) amongst other things for citations. I do not find this in the material. This is a major omission. We need a list of all the included studies with a proper citation so readers can investigate the original sources. It would also be beneficial to have citations for all the excluded studies. This would be especially valuable so that readers can look at the ones excluded for language in case they can read the relevant language themselves or they have access to human or machine translation facilities.

We could also use some more descriptive statistics about the studies. I would suggest a frequency table of countries involved possibly broken down by cascade topic or intervention type.

Seeing the wood for the trees

The authors have presented their results broken down by every combination of cascade stage and intervention type leading to many (more than 50) individual statements each based on a small number of primary studies. There seems to be a median of 3 primary studies per effect size in Figure 3. Apart from the issue that with so many comparisons confidence intervals and statistical significance statement need to be qualified it seems to me to lead to a confused picture. I would suggest that the authors consider performing a meta–regression for each cascade stage including intervention type as a moderator. This would enable us to see a forest plot for each which would show us the individual studies which go to make up each analysis, something which is very hard to disentangle at present. We would also see much more directly which intervention was more effective, if any. Alternatively the analysis could be by treatment type with cascade stage as a moderator if that is more interesting for the science or clinical readership.

Summary

Quite a few comments for clarification and enhancement. As it stands the authors have put a great deal of effort into the earlier stages of the review but have not, as I see it, extracted the maximum from the data–set they have so laboriously assembled.

Michael Dewey

References

J L Jackson, A Kuriyama, A Anton, A Choi, J-P Fournier, A-K Geier, F Jacquerioz, D Kogan, and R Sun. The accuracy of Google Translate for abstracting data from non–English–language trials for systematic reviews. *Annals of Internal Medicine*, 171:677–679, 2019.

C Kieling, H Herrman, V Patel, and J J Mari. Indexation of psychiatric journals from low– and middle–income countries: a survey and a case study. *World Psychiatry*, 8:40–44, 2009.

K Pilkington, A Boshnakova, M Clarke, and J Richardson. 'No language restrictions' in database searches: what does this really mean? *The Journal of Alternative and Complementary Medicine*, 11:205–207, 2005.

Y Shenderovich, M Eisner, C Mikton, F Gardner, J Liu, and J Murray. Methods for conducting systematic reviews of risk factors in low– and middle–income countries. *BMC Medical Research Methodology*, 16(32), 2016.

R J Syed Sheriff, C E Adams, P Tharyan, M Jayaram, L Duley, and the PRACTIHC Mental Health Group. Randomised trials relevant to mental health conducted in low and middle–income countries: a survey. *BMC Psychiatry*, 8(69), 2008.
