# Supplementary material for: Improving cascade outcomes for active TB: A global systematic review and meta-analysis of TB interventions
Source: PLoS Med. 2023 Jan 3;20(1):e1004091. doi: 10.1371/journal.pmed.1004091 (PMC9847969; doi:10.1371/journal.pmed.1004091)
Supplement: Attachment — Submitted filename: marley2.pdf [file pmed.1004091.s010.pdf]

This paper reports a systematic review with meta–analysis of interventions in the care of active tuberculosis.

I was asked for a statistical report and I interpret that to include all aspects of the design and conduct of the study.

I said:

> *The language restriction mentioned on page 3 in the abstract should also be mentioned here. More importantly why was such a restriction imposed? If a high proportion of studies were expected to be in low– and middle–income countries then it seems too restrictive. For a condition which has a major impact in resource poor countries bibliographic databases with a coverage of the literature of those countries would seem indicated. Various empirical studies have indicated that the main bibliographic databases have varied and incomplete coverage of material not in English (Pilkington et al., 2005; Shenderovich et al., 2016), and have limited coverage of material from low and middle–income country journals (Kieling et al., 2009; Syed Sheriff et al., 2008). A wider selection of data–bases and inclusion of material other than in English would seem valuable. The authors might wish to read Jackson et al. (2019) for a brief report on using machine translation in systematic reviewing.*

I do not find the authors' rebuttal here very convincing. This is a funded study and could have included a budget for professional translation if indeed all the authors are monoglot English speakers. The reference which they quote in the rebuttal to the editors does not give us enough detail specifically whether the authors of the Cochrane reviews searched for non–English language articles or whether they relied on the standard databases in which case they would have found fewer relevant studies. The message I draw from Grégoire et al. (1995); Moher et al. (1996); Egger et al. (1997); Jüni et al. (2002); Konno et al. (2020); Nussbaumer-Streit et al. (2020); Bachelet et al. (2021) is that bias is possible although it is hard to predict in advance the extent or direction of it. The authors also refer to their past experience with translation software but this ignores the fact that this is a field where considerable effort has gone into improving performance. Google Translate for instance is currently swapping from a purely statistical approach to one based on a neural network. Most of the languages which commonly occur in scientific writing have now swapped to the new system.

Michael Dewey

References

V C Bachelet, M S Navarrete, C Barrera-Riquelme, V A Carrasco, M Dallaserra, R A Díaz, A A Ibarra, F J Lizana, N Mezza-Ducaud, M G Saavedra, C Tapia-Davegnoa, A F Vergara, and J Villanueva. A multiyear systematic survey of the quality of reporting for randomised trials in dentistry, neurology, and geriatrics published in journals of Spain and Latin America. *BMC Medical Research Methodology*, 21(153), 2021.

M Egger, T Zellweger-Zähner, M Schneider, C Junker, C Lengeler, and G Antes. Language bias in randomised controlled trials published in English and German. *Lancet*, 350:326–329, 1997.

G Grégoire, F Derderian, and J Le Lorier. Selecting the language of the publications included in a meta-analysis: is there a tower of Babel bias? *Journal of Clinical Epidemiology*, 48(1):159–163, 1995.

J L Jackson, A Kuriyama, A Anton, A Choi, J-P Fournier, A-K Geier, F Jacquerioz, D Kogan, and R Sun. The accuracy of Google Translate for abstracting data from non–English–language trials for systematic reviews. *Annals of Internal Medicine*, 171:677–679, 2019.

P Jüni, F Holenstein, J Sterne, C Bartlett, and M Egger. Direction and impact of language bias in meta–analyses of controlled trials: empirical study. *International Jounal of Epidemiology*, 31:115–123, 2002.

C Kieling, H Herrman, V Patel, and J J Mari. Indexation of psychiatric journals from low– and middle–income countries: a survey and a case study. *World Psychiatry*, 8:40–44, 2009.

K Konno, M Akasaka, C Koshida, N Katayama, N Osada, R Spake, and T Amano. Ignoring non–English–language studies may bias ecological meta–analyses. *Ecology and Evolution*, 10:6373–6384, 2020.

D Moher, P Fortin, A R Jadad, P Jüni, T Klassen, J Le Lorier, A Liberati, K Linde, and A Penna. Completeness of reporting of trials published in languages other than English: implications for conduct and reporting of systematic reviews. *Lancet*, 347:363–366, 1996.

B Nussbaumer-Streit, I Klerings, A I Dobrescu, E Persad, A Stevens, C Garrity, C Kamel, L Affengruber, V J King, and G Gartlehner. Excluding non–english publications from evidence–syntheses did not change conclusions: a meta–epidemiological study. *Journal of Clinical Epidemiology*, 118:42–54, 2020.

K Pilkington, A Boshnakova, M Clarke, and J Richardson. ‘No language

restrictions' in database searches: what does this really mean? *The Journal of Alternative and Complementary Medicine*, 11:205–207, 2005.

Y Shenderovich, M Eisner, C Mikton, F Gardner, J Liu, and J Murray. Methods for conducting systematic reviews of risk factors in low– and middle–income countries. *BMC Medical Research Methodology*, 16(32), 2016.

R J Syed Sheriff, C E Adams, P Tharyan, M Jayaram, L Duley, and the PRACTIHC Mental Health Group. Randomised trials relevant to mental health conducted in low and middle–income countries: a survey. *BMC Psychiatry*, 8(69), 2008.
