# Supplementary material for: Improving cascade outcomes for active TB: A global systematic review and meta-analysis of TB interventions
Source: PLoS Med. 2023 Jan 3;20(1):e1004091. doi: 10.1371/journal.pmed.1004091 (PMC9847969; doi:10.1371/journal.pmed.1004091)
Supplement: Attachment — Submitted filename: Plos Med Editor Comments.edited.docx [file pmed.1004091.s011.docx]

Dear Editors,

Thank you for your helpful editorial and reviewer comments and interest in accepting our manuscript for publication. We have revised the manuscript according to the editorial requests suggested.

Please find below our point-by-point response to the editor’s comments.

Thank you.

*Comments from the Academic Editor:*

I think the authors did a very good job in revising this and addressing the comments of all reviewers (except of the point on language). While the statistical reviewer is right about the limitations of only including English literature, this is an excellent review of the English literature. Furthermore, including other language at this point would take a lot of time and is not really possible within the purview of a revision. From my reading of the literature, I don't expect substantial bias.

**Response: Thank you.**

*Requests from Editors:*

Abstract Methods and Findings:

1) Please include the study design (systematic review and meta-analysis).

**Response: We have included the study design on line 32 of the Abstract in the statement, "In this systematic review and meta-analysis, we retrieved English articles with comparator arms (like randomized controlled trials and before and after intervention studies) that evaluated TB interventions published from January 1970 to September 30, 2022, from Embase, CINAHL, PubMed, and the Cochrane library.”**

2) The sentence "Also, our study does not cover the entire care cascade as we did not measure gaps in pre-testing, pretreatment, and post-treatment outcomes (like loss-to-follow-up and TB recurrence)" should be relocated to the last sentence of the previous section (Abstract Methods and Findings).

**Response: We have moved the sentence "Also, our study does not cover the entire care cascade as we did not measure gaps in pre-testing, pretreatment, and post-treatment outcomes (like loss-to-follow-up and TB recurrence)" to lines 52-54 of the Abstract findings.**

3) Please include the decision not to include non-English language sources as a limitation in the Discussion.

**Response: We have included this limitation in the statement, "We also excluded non-English studies which may have impacted the capture of literature from the bibliographic databases of poor countries and biased our findings" on lines 54-547 of the discussion section.**

Author Summary:

* Please reformat your Author Summary to comprise 2-3 single sentence bullet points for each of the three questions.

**Response: We have reformatted the Author summary to comprise three single sentence points for each of the three questions on lines 70-91 of the Author summary.**

* Under the ‘What Did the Researchers Do and Find?’ question, please include the headline numbers from the study, such as the number of studies included and key findings.\

**Response: We have stated the number of included studies and specified the five main interventions associated with multiple TB care cascade outcomes on lines 78-82 of the author summary in the statements:**

- **We systematically reviewed and meta-analyzed evidence on TB interventions and their effects on the TB care cascade for active TB from 144 peer-reviewed studies.**
- **In this study, five out of 12 identified TB interventions associated with multiple care cascade outcomes were education and counseling, incentives, digital interventions, community-based, multi-sector collaborations, and mixed interventions.**

*Lines 105-106: Please temper claims of primacy of results by stating, "to our knowledge" or something similar.

**Response: We have tempered this primacy claim in the statement, "Moreover, previous reviews have mainly focused on synthesizing evidence of interventions on single care cascade outcomes- per our knowledge" on lines 106-107 of the Introduction.**

*Please update your PRISMA checklist to use section and paragraph numbers rather than line numbers (as these will not be present in the published version).

**Response: We have updated the PRISMA checklist to use section and paragraph numbers only. Thank you**

*Please add the following statement, or similar, to the Methods: "This study is reported as per the Preferred Reporting Items for Systematic Reviews and Meta-Analyses (PRISMA) guideline (S1 Checklist)."

**Response: We have added the statement "This study is reported as per the Preferred Reporting Items for Systematic Reviews and Meta-Analyses (PRISMA) guideline (S1 Checklist)" on lines 222-223 of the Methodology section.**

*Line 137: I could not locate S1 Table in the Supporting Information.

**Response: We have rectified this error and stated that “Details of search outcomes are in S2 Table” on lines 141-142.**

*Line 153: Figure 2 does not appear to relate to this statement.

**Response: This was an oversight and has been deleted. We have stated on lines 231-232 that "We identified a total of 12 major TB interventions across the six care cascade outcomes of interest [Figure 2].”**

*Please revise Figure 2 such that the word ‘Interventions’ does not break across two lines.

**Response: We have revised Figure 2 as recommended. Thank you**

*****Table 2: Please define the abbreviation MDR in the figure legend.

**Response: We have stated “MDR=Multidrug-resistant TB” in the Table 2 legend and the List of Abbreviations.**

*Line 237: Please delete the duplicate [S6 Table] citation here.

**Response: We have deleted the duplicate [s6 Table] on line 245.**

*In Figures 3A through 3F, please ensure that the X axis is identical for all figures to facilitate comparison.

**Response: We have ensured that the X-axis in figures 3A through 3F is all identical in the revised figures.**

*The title of S9 Figure Panel A (TB Testing) has been cut off during creation of the Figure.

**Response: We have rectified this in the new S9 Figure submitted.**

*Line 402: I could not locate Table 5.

**Response: We have corrected this to cite Table 4. We apologize for the oversight.**

S11 Figure appears to also duplicate Figures S9 and S10.

**Response: We have rectified this problem in the revised S11 Figure. Thank you.**

*Line 825: Error in the numbering of the References here, please delete.

**Response: Sorry, we could not determine which specific reference had the error in numbering per your comment, as the line numbers shifted while we revised the paper. However, we have updated the reference list and hope this error in numbering is resolved. If this persists, kindly let us know the specific reference number, and we will delete it accordingly. Thank you**

*Reviewer #4: I have no further comments

The new Tables are very helpful and clear

I stand corrected on the linkage to care definition

thank you.

**Response: Thank you for the helpful comments.**
