# Supplementary material for: Improving cascade outcomes for active TB: A global systematic review and meta-analysis of TB interventions
Source: PLoS Med. 2023 Jan 3;20(1):e1004091. doi: 10.1371/journal.pmed.1004091 (PMC9847969; doi:10.1371/journal.pmed.1004091)
Supplement: Attachment — Submitted filename: PLOS Medicine Reviewer Comments 09Nov2022_Final.docx [file pmed.1004091.s009.docx]

**Reviewer Response Letter**

Dear Editor,

Thank you for your interest in our manuscript and for the helpful reviewer comments. We have revised the manuscript according to the recommendations and suggestions made.

Please find below our point-by-point response to the reviewer's questions.

Thank you.

Abstract:
1. Please ensure that all numbers presented in the abstract are present and identical to numbers presented in the main manuscript text.

**Response: Thank you for the comment. We have double checked all numbers in the manuscript and abstract to ensure cohesion.**

2. Please combine the Methods and Findings sections into one section, "Methods and findings".

**Response: We have combined these sections.**

3. Please include the study design, dates of search, types of study designs included, and eligibility criteria.

**Response: we have specified in lines 35-39 of the abstract that "Only original research, published from 1970 to September 2022, and had standard-of-care comparator arms were eligible for inclusion. Commentaries, letters, qualitative studies, conference abstracts with limited information on intervention approaches, and studies that did not report quantitative results for TB care cascade outcomes were excluded".**

4. In the last sentence of the Abstract Methods and Findings section, please describe the main limitation(s) of the study's methodology.

**Response: we mentioned this on line 56-58 of the abstract in the statement that "Also, our study does not cover the entire care cascade as we did not measure gaps in pre-testing, pretreatment, and post-treatment outcomes (like loss-to-follow-up and TB recurrence). "**

5. The Financial Disclosure should describe sources of funding that have supported the work. If your manuscript is published, your statement will appear in the Funding section of the article. See <https://journals.plos.org/plosmedicine/s/submission-guidelines#loc-financial-disclosure-statement> for more information. Please remove the 'Funding' section from the main text and instead include this information in the Financial Disclosure section of the Submission Form.

**Response: We have removed the funding section from the manuscript**

6. Please remove the 'Summary' section and instead include a short, non-technical Author Summary of your research to make findings accessible to a wide audience that includes both scientists and non-scientists. The Author Summary should immediately follow the Abstract in your revised manuscript. This text is subject to editorial change and should be distinct from the scientific abstract. Please see our author guidelines for more information: <https://journals.plos.org/plosmedicine/s/revising-your-manuscript#loc-author-summary>

**Response: We have removed the ‘Summary’ and included an ‘Author summary after the Abstract on lines 68-88.**

7. Please include continuous line numbering throughout your manuscript.
**Response: We have included continuous line numbering throughout the manuscript.**

8. Please place citations in square rather than round brackets.
**Response: We have replaced the citation round brackets with square brackets.**

9. Please label items in the Supporting Information as described here: <https://journals.plos.org/plosmedicine/s/supporting-information>

**Response: We have labeled all items in the supplementary file following the prescribed guidelines. Thank you**

10. Please update your search to the present time.

**Response: We have updated the search to capture all studies published until 30^th^ September, 2022.**

11. Please provide the beginning and end dates of your search in the Methods.

**Response: We have included the search dates on line 34 of the abstract in the statement "Only original research, published from January 1970 to September 30, 2022..."**

12. Please include non-English language sources of studies.
**Response: Thank you for the recommendation. However, the team, like many other teams (**[**https://systematicreviewsjournal.biomedcentral.com/articles/10.1186/s13643-018-0786-6**](https://systematicreviewsjournal.biomedcentral.com/articles/10.1186/s13643-018-0786-6)**), lacked resources and funding for translation services. And although using online translation softwares could help circumvent the language barrier to eligibility, online translations of scientific manuscripts are not always accurate as sentence meanings easily get misinterpreted. Without a native speaker to verify the translation before inclusion, we are likely to include misinformation and introduce additional bias in our interpretation of the findings.**

**Although this decision may have some language bias, this cochrane abstract (**[**https://abstracts.cochrane.org/2019-santiago/excluding-non-english-studies-systematic-reviews-does-not-change-conclusions-meta**](https://abstracts.cochrane.org/2019-santiago/excluding-non-english-studies-systematic-reviews-does-not-change-conclusions-meta)**) showed that excluding non-English studies did not affect study conclusions.**

13. Please remove the Contributors, Conflicts of interest, and Data sharing sections from your main text – all of this information ought to be included in your responses to the relevant parts of the submission form.

**Response: We have removed this information and entered it at the relevant parts of the submission.**

14. Thank you for reporting your SR/MA according to the PRISMA guidelines. Please update the checklist to use section and paragraph numbers, rather than page numbers.

**Response: We have updated the PRISMA checklist with sections and paragraph numbers as directed. Thank you**

15. You note in your 'in text' Data sharing section that further data are available on request from the corresponding author. PLOS does not permit the corresponding author to be the main point of contact for data requests. Please relocate the data sharing statement to the relevant part of the submission form and see <https://journals.plos.org/plosmedicine/s/data-availability> for further information on how best to make your data available.
**Response: We will make all relevant data available as supplementary files.**

**Response to the reviewers:**

Reviewer 1. This paper reports a systematic review with meta–analysis of interventions

in the care of active tuberculosis. I was asked for a statistical report and I interpret that to include all aspects of the design and conduct of the study.

**Points of detail**

**Page 6** The language restriction mentioned on page 3 in the abstract should

also be mentioned here. More importantly why was such a restriction imposed? If a high proportion of studies were expected to be in low– and middle–income countries then it seems too restrictive. For a condition which has a major impact in resource poor countries bibliographic databases with a coverage of the literature of those countries would seem indicated. Various empirical studies have indicated that the main bibliographic databases have varied and incomplete coverage of material not in English (Pilkington et al., 2005; Shenderovich et al., 2016), and have limited coverage of material from low and middle– income country journals (Kieling et al., 2009; Syed Sheriff et al., 2008). A wider selection of data–bases and inclusion of material other than in English would seem valuable. The authors might wish to read Jackson et al. (2019) for a brief report on using machine translation in systematic reviewing.

**Response: Thank you. We have considered your recommendation to use online translators to enable us include non-English publications and reviewed the informative materials you suggested. We also agree that some relevant studies may be missed due to this eligibility criteria. However, we have decided to keep this criteria due to lessons from past experiences of using online translator software to circumvent language barriers. Whiles this method may be helpful with spoken language, the content of scientific publications easily get lost or miscommunicated after translation. And without a native speaker available to audit such translated documents before their inclusion, we are likely to introduce additional errors into the review process.**

**Page 6** Why exclude dissertations?

**Response: We excluded dissertations because findings dissertations are not always externally peer-reviewed and the major findings from most dissertations are usually published. As such, we are likely to have captured these studies in our literature retrieval already.**

**Page 6** I can see that previous systematic reviews are not suitable for the meta–analysis but they are presumably a source of studies which might otherwise be missed. Similarly did the authors use the reference lists of the studies they did find to identify possible other studies? Figure refers to hand searching but without clear explanation of what that means.

**Response: Yes we screened through the references of similar systematic reviews to identify eligible studies by title that may have been missed in the database search. This process of manually screening through the reference list of other systematic reviews is what we termed “hand-searched”.**

**Page 7** I think there is an ambiguity possible here. ‘. . . without evidence of failure or a record to show a sputum smear or . . . ’. Does this mean ‘. . . without (evidence of failure) or (a record to show a sputum smear or . . . )’ ‘. . . without evidence of (failure) or (a record to show a sputum smear or . . . )’?

**Response: We have revised this definition in Table 1 and on line 165-177 under the definitions paragraph to make it clearer.**

**Page 8** I do not see what ‘similar comparator arms and intervention strategies’ means. I thought the whole point of the authors’ review was to include a wide range of comparators and strategies. It would be helpful to clarify what comparators were included. Are these all trials against treatment as usual or are some head–to–head? If the latter then network meta–analysis would help to compare different treatment options.

**Response: Sorry for the confusion. While the aim of our review is to include a wide range of strategies, pooling data to inform our outcomes will be difficult if all the interventions are compared to different controls. To standardize the appraisal process and assessment of interventions effect well, we only pooled studies whose comparator arms (control groups) was standard TB care (treatment as usual).**

**Page 8** The phrase publication bias implies you know the aetiology. There are other ways in which small studies may differ and some of these like case–mix might well be an issue here.

**Response: Thank you for pointing this out. We agree with you and also believe there may be case-mix bias contributing to this finding.**

**Page 10** Having said that the small numbers preclude looking at small sample effects the authors then go on to present the results of Egger’s test on some very small numbers. This seems flatly contradictory.

**Response: We agree with your comment. This was an oversight as the paragraph was meant to deleted. We have rectified this in the revised manuscript.**

**Page 15** Here the authors state ‘digital interventions was not significantly associated with any’ but on page 10 they report ‘digital interventions [. . . ] were each associated with increased likelihood of testing.’ This seems flatly contradictory.

**Response: This was an oversight. We have rectified the statement on line 458 under the discussion section to “Digital interventions only increased the likelihood of TB testing and linkage to care”. This correction is shown by the results of our updated results analysis.**

**Supplementary Figure 2** On page 10 the authors state that all of the plots show evidence of asymmetry. All of the sub–tables would benefit from telling us the value of the statistic from Egger’s method.

**Response: We have re-ran the funnel plots and egger’s test based on updated data. We have also presented the results of funnel plots with ‘trim and fill’ adjustments as supplementary figures (S8 Figure) for easier accessibility.**

**2.1** The *y*–axis means everything is compressed at the top. I see no evidence of systematic small study effects here contrary to the statement on page 10. There is an outlying value.

**2.2** This one is more worrying I agree.

**2.3** The *y*–axis could use adjustment and there are a couple of outliers but little evidence of systematic small study effects.

**2.4** Remember that Egger’s method is a regression. The green square bottom right will be probably be having a massive influence on the fitted model.

**2.5** Again *y*–axis and again little to worry about here.

**2.6** It is hard to see what is going on here because the three types of plot points overlap one another so we cannot see where, if anywhere, things are going wrong.

**Supplementary Table 6** Contrary to the caption this does not show regional differences.

**Response: This table has been removed.**

**Points of more substance**

**Detail of reporting**

The PRISMA guidelines ask (requirement 18) amongst other things for citations. I do not find this in the material. This is a major omission. We need a list of all the included studies with a proper citation so readers can investigate the original sources. It would also be beneficial to have citations for all the excluded studies. This would be especially valuable so that readers can look at the ones excluded for language in case they can read the relevant language themselves or they have access to human or machine translation facilities.

We could also use some more descriptive statistics about the studies. I would suggest a frequency table of countries involved possibly broken down by cascade topic or intervention type.

**Response: Thank you. Per your recommendation, we have included a summary descriptive table as Table 2. We have also cited all included studies, however, a citation of our over 1000 excluded studies is not possible. We have also made available a detailed data extraction list of all our included studies as supporting file (S13 Table).**

**Seeing the wood for the trees**

The authors have presented their results broken down by every combination of cascade stage and intervention type leading to many (more than 50) individual statements each based on a small number of primary studies. There seems to be a median of 3 primary studies per effect size in Figure 3. Apart from the issue that with so many comparisons confidence intervals and statistical significance statement need to be qualified it seems to me to lead to a confused picture. I would suggest that the authors consider performing a meta–regression for each cascade stage including intervention type as a moderator. This would enable us to see a forest plot for each which would show us the individual studies which go to make up each analysis, something which is very hard to disentangle at present. We would also see much more directly which intervention was more effective, if any. Alternatively the analysis could be by treatment type with cascade stage as a moderator if that is more interesting for the science or clinical readership.

**Response: Thank you for the recommendation. We have run the meta-regression and presented the results on line 245-260 under the results section. A table with all details from the meta-regression is also available in the supplementary file (S4 Table).**

**Summary**

Quite a few comments for clarification and enhancement. As it stands the authors have put a great deal of effort into the earlier stages of the review but have not, as I see it, extracted the maximum from the data–set they have so laboriously assembled.

**Response: Thank you for the recommendations and suggestions to help ensure that we extract the maximum information from our data-set.**

**References**

J L Jackson, A Kuriyama, A Anton, A Choi, J-P Fournier, A-K Geier, F Jacquerioz, D Kogan, and R Sun. The accuracy of Google Translate for abstracting data from non–English–language trials for systematic reviews. *Annals of Internal Medicine*, 171:677–679, 2019.

C Kieling, H Herrman, V Patel, and J J Mari. Indexation of psychiatric journals from low– and middle–income countries: a survey and a case study. *World Psychiatry*, 8:40–44, 2009.

K Pilkington, A Boshnakova, M Clarke, and J Richardson. ‘No language restrictions’ in database searches: what does this really mean? *The Journal of Alternative and Complementary Medicine*, 11:205–207, 2005.

Y Shenderovich, M Eisner, C Mikton, F Gardner, J Liu, and J Murray. Methods for conducting systematic reviews of risk factors in low– and middle–income countries. *BMC Medical Research Methodology*, 16(32), 2016.

R J Syed Sheriffff, C E Adams, P Tharyan, M Jayaram, L Duley, and the PRACTIHC Mental Health Group. Randomised trials relevant to mental health conducted in low and middle–income countries: a survey. *BMC Psychiatry*, 8(69), 2008.

Page 4

Reviewer #2: PMEDICINE-D-22-02722R1: Improving Continuum Outcomes for Active TB: A Global Systematic Review and Meta-Analysis of TB Interventions.
Overall, the systematic review and metanalysis on this topic is highly relevant and I must congratulate the authors for taking this up.
The author should have given continuous line numbers in their manuscript file so that would have helped the reviewer in indicating the line numbers while making the comments. Nevertheless, the following are the major comments from me.

**Response: Thank you for reviewing our manuscript and sorry about the missing line numbers in the earlier version of the manuscript. We have included continuous line numbers in the revised manuscript.**

1.      The major problem with this manuscript is that several aspects of methodology and the results are available in the supplementary file and not as the main manuscript. This needs to be corrected.

**Response: We agree with your comment and have moved details of search terms, search strategy, and the quality assessment process from the supplementary file to the main manuscript under the method section on lines 119-136, quality assessment paragraph lines 210-218 and results section lines 40-447 to ease comprehension and accessibility.**

2.      The literature review was last updated till April 2021. The authors are requested to update their systematic review and include any new studies that have been published, so that their manuscript is up to date.

**Response: Thanks for the suggestion. Per the suggestion, we have updated the search to capture all studies published until 30^th^ September, 2022.**

3.      The specific objectives highlighting the PICO (target patients, interventions, control population, outcomes) elements for each step/multiple steps in the care continuum needs to be spelt out in the main manuscript. These are currently seen only in the supplementary tables.

**Response: We have moved this details to the method section of the main manuscript, lines 119-136.**

4.      The search terms must be derived from these objectives for each PICO element.  The search terms mentioned under methods is not of sufficient detail and the exact terms and search methodology for searching for articles in each of the databases needs to be mentioned very clearly. These are listed in the supplementary files and needs to be brought out in the main manuscript.

**Response: We agree with your comments and have moved these details to the main manuscript in the methodology section lines 119-136.**

5.      The operational definition given in Table 1 of what is considered as an intervention and what is not considered as an intervention in this systematic review needs to be spelt out. For example, if the intervention includes a diagnostic tool such as Xpert TB/Rif® test introduction or introduction of a newer TB treatment regimen, whether this is considered an intervention to improve TB care continuum is unclear.

**Response: Thank the reviewer for the suggestion. To better clarify this, we have included a definition of what we considered an intervention in row 2 of table one stating that interventions in this review was defined as ‘Any initiative implemented to improve TB care outcomes. This includes:**

**policy introduction or change (like updating clinical guidelines, integrating TB care with other services, establishing new referral procedures, and fostering collaboration between departments)**

**provision of tools and resources to improve case detection and treatment outcomes (like providing X-ray machines, microscopes, GeneXpert machines, pill boxes, etc.)**

**capacity building (like staff training, providing educational materials for TB patients, and engaging lay workers in services delivery).’**

6. The description of the intervention that was picked up from each study needs to be more elaborate. For example, describing an intervention as "Education & Counselling" without indicating what are the element of this intervention is not useful to understand this intervention and replicate it in future.

**Response: We have included further description of each intervention we identified and examples of how they were designed in Table 1. For example, we have stated as part of our definition of Education and Counseling in Table 1 that ‘Impacting knowledge about TB or self-care TB to patients through information materials dissemination, one-on-one talk sessions, and public education (like peer education and in-school talk sessions). Most studies used the term 'counseling' loosely to describe one-on-one sessions between designated counsellors and TB patients about TB treatment, the need for adherence and coping with adverse effects. Types of education and counseling interventions identified through this review included:**

- ***support groups* (TB patients form/join groups to support each other psychologically and emotionally through the treatment journey)**
- ***psychosocial education and counseling* (trained counsellors counsel newly diagnosed TB patients and patients on treatment on TB infection and treatment)**
- ***lay counseling* (community lay workers engaged to provide home-based care and adherence support for TB patients)**
- ***public education* (healthcare workers or lay counselors engage communities and household member of newly diagnosed TB patients/high-risk populations on TB infection, risks of transmission and the need to get tested**
- ***practice-based staff education* (tuberculosis specialists visit intervention clinic sites to promote tuberculosis screening, raise awareness of tb as a local public health concern, and distributed copies of local tuberculosis screening guidelines among healthcare providers)**
- ***peer education* (peer educator volunteers recruited and trained on TB to educate peers within specified geographical locations on TB transmission, TB risk groups, how treatment is conducted, the importance of screening).’**

7. Also, regarding interventions, was there a dose response relationship? More intensive education and counselling versus less intensive education and counselling? This sort is description is needed for each intervention described in this manuscript.

Response:

**Response: We do not have this information as we did not measure dose response relationship with intervention effects. However, we did acknowledge that that the intensity of interventions like counseling and education was associated with the effects on TB care outcomes and the variations in intensity contributed to the high heterogenity observed. We have noted this in the results section line 245-260 and the discussion section line 498-511.**

8. The quality of all the figures/images is very poor and the text is not easily readable. This needs to be replaced.

**Response: We apologize for this and have uploaded much clearer figures for your perusal.**

**Reviewer #3:**

This is a very large systematic review that makes two innovative contributions to the global TB literature. First, most systematic reviews of TB care interventions focus on TB treatment, whereas numerous studies of the care cascade have shown that the largest losses of people with TB occur well before individuals get to initiate TB treatment. As such, by conducting a review that evaluates interventions across multiple stages of the care cascade, the authors go beyond the scope of most systematic reviews in this space and address stages that receive less attention. Second, evaluating interventions for all of these stages together allows for comparison and provides important insights. For example, the most notable finding—regarding the potential benefits of counseling across multiple stages of care, is very helpful to TB programs, because it allows for focused investment in this intervention to address multiple gaps in care.

**Response: Thank you**

With that said, the challenge with this review is dealing with the problem of heterogeneity—by study setting (e.g., high-income vs. lower income countries), patient population, and with regard to the interventions themselves, among other forms of heterogeneity. The authors have done a reasonable job of dealing with the former two problems but do not provide enough information for readers to understand what these interventions across the various studies might involve. This is a shortcoming of the paper and limits the insights if provides—but I feel that the authors could address this shortcoming by providing a table or tables that provide more specific insights into the types of interventions that were found to be potentially impactful a cross various gaps.

**Response: Thank you for this helpful comment. We have updated table one in the manuscript to provide specific details of each identified intervention with description of characteristics that varied within each intervention. We have also elaborated more on how each intervention was designed and used during implementation in different studies to provide a better understanding of how similar interventions still differed at the implementation level in Table 1 . We have also made the list of included studies with details of their intervention strategies accessible in the supplementary file (S3 Table).**

Another major concern is regarding the authors' search strategy—which does not seems sufficiently broad to cover all cascade gaps, as discussed further below.

Major feedback:

1.      Search strategy does not seem sufficiently broad: A major concern is that the authors' search strategy does not appear sufficiently broad to actually cover care cascade gaps outside of the TB treatment phase. In fact, I worry that it is not adequate to even cover all interventions in the treatment phase. The umbrella search terms all seem focused on medication adherence—e.g., "adherence", "compliance", "retention", etc. None of these seems appropriate to cover early stages in the cascade focused on testing or pretreatment loss to follow-up. For example, there is no term for "testing", "diagnosis", "linkage to care", "loss to follow-up" (and related variants, such as "initial default", the old term for pretreatment loss to follow-up) which would address gaps in testing or pretreatment loss to follow-up, or on-treatment loss to follow-up for that matter. I am in fact worried that the authors may have initially aimed to conduct a review of interventions for medication adherence and then repurposed the search findings to look across the care cascade. The authors need to present evidence to suggest that gaps in testing, diagnosis, and linkage to care were actually somehow covered in their search, otherwise I'm worried this is a major shortcoming of this review as it's unclear how they would have adequately covered this literature.

# Response: We thank the suggestions. This systematic review and meta-analysis was designed to evaluate the effectiveness of progamtatic interventions in improving the TB care continuum. For the cascade, we adapted the suggestions from the novel framework for monitoring and evaluation of the cascade of HIV-associated TB care at the health facility level used by Hanran and Van in 2017. This included screening and testing to treatment success and prevention. However, our key focus on active TB ended our cascade on treatment success . Per the suggestions of the reviewer, we have updated our overall search strategy to include "testing", "diagnosis", and "linkage to care" as part of our search update to ensure all studies related to our outcomes of interest are captured. Details of the updated search terms and strategy are noted on lines 103-120 under the methods section. Early stages in the cascade focused on testing or pre-treatment loss to follow-up were not outcomes of interest we planned to measure per our study protocol. Hence we did not include that terms related to these outcomes such as pre-treatment loss to follow-up, or on-treatment loss to follow-up in our search strategy. We have noted this in the limitation paragraph on line 540-545 of the discussion section.

# Reference: Hanrahan CF, Van Rie A. A proposed novel framework for monitoring and evaluation of the cascade of HIV-associated TB care at the health facility level. J Int AIDS Soc. 2017 Apr 20;20(1):21375. doi: 10.7448/IAS.20.01.21375. PMID: 28440604; PMCID: PMC5515049. 2.      Definition of "testing" does not cover important testing modalities and confusion between "diagnosis" and "testing": The authors' definition of "testing" (page 7, first lines under "Definitions") does not appear to address Xpert MTB/RIF (or other cartridge-based nucleic acid amplification testing methods such as TrueNat) or mycobacterial culture. It also does not address testing for extrapulmonary TB. It is unclear why some of these modalities fall under the "diagnosis" gap rather than the "testing" gap. For example, how is the process of "administering sputum smear", which counts as testing, any different from the process of administering Xpert MTB/RIF for upfront testing? This raises the question of whether the "testing" and "diagnosis" gaps are really a single gap - as is articulated in most TB care cascades. Again, a contributing factor to this confusion is that it is unclear what the interventions included in the review actually entail—and therefore how addressing "testing" vs. "diagnosis" is different.

**Response: We agree with your comment about the confusion in how we defined these 2 terminologies. Most of the testing you mentioned are diagnostic tests and not screening tests, which is why we listed them under the diagnosis gap. We have revised the definitions to be clearer. We have stated in the definition section line 147-152 that "Testing in this review refers to** **the initial screening tests administered to persons suspected to have TB after risk and symptoms evaluation". It aims to sort persons who probably have a disease from those who do not and is not intended to be a diagnostic test. Screening tests use a simpler testing process (usually a sputum smear test) after which persons with positive or clinically suspicious results (like inconclusive results) are referred for diagnostic testing". We have also dated the definition of diagnosis on lines 154-158 under the definition section and clarified these definitions in table one.**

**References:**

**- Lönnroth K, Corbett E, Golub J, Godfrey-Faussett P, Uplekar M, Weil D, et al. Systematic screening for active tuberculosis: rationale, definitions and key considerations. Int J Tuberc Lung Dis. 2013;17(3):289-98.**

**- Assefa Y, Woldeyohannes S, Gelaw YA, Hamada Y, Getahun H. Screening tools to exclude active pulmonary TB in high TB burden countries: systematic review and meta-analysis. Int J Tuberc Lung Dis. 2019;23(6):728-34.**

**- van’t Hoog A, editor Sensitivity and specificity of different TB screening tools and approaches: a systematic review. 43rd Union World Conference on Lung Health, Kuala Lumpur, Malaysia; 2012.**

**- Chris Coulter. Tuberculosis testing. Australian Journal for General Practitioners. 2012;41:489-92.**

**- World Health Organization. Diagnostic testing for TB, HIV-associated TB and drug-resistant TB. Available from:** [**https://www.who.int/publications/digital/global-tuberculosis-report-2021/tb-diagnosis-treatment/diagnostic-testing**](https://www.who.int/publications/digital/global-tuberculosis-report-2021/tb-diagnosis-treatment/diagnostic-testing)**. Accessed October 30, 2022.**

3.      Need to provide more insights into what interventions actually entail. While the authors do define the broad intervention categories they use, I constantly found myself wondering whether more specific information could be provided on specific characteristics of the interventions to make this review more useful. For example, what were characteristics of the "community-based interventions" or "mixed interventions" that positive impacted outcomes for each outcome? Just as the authors provide a Forest Plot for each outcome, it would greatly increase the value of this review to supply one table per outcome in the main manuscript that provides more details on the specific characteristics of interventions based on the original paper. For example, for "testing" there were 2 studies involving digital interventions, 7 studies involving incentives, 9 studies involving counseling, 6 studies involving active case finding, and 2 studies involving multi-sector collaboration. A single table could very briefly summarize the specific interventions used in each study (with one row per intervention type, e.g., "incentives"). This would allow readers to gain a more specific understanding of what these interventions actually are.

**Response: We agree with your concerns and recommendations. We have updated the definitions of each intervention and insights into what the interventions entailed in table 1. We have included a summary showcasing the number of studies reporting each type of intervention and outcome in Table 2. We have also made an excel spreadsheet available with details of the interventions used in each study as part of data sharing and supplied tables per cascade outcome in the supplementary file (S3 Table) .**
+
4.      Need to discuss implications of the funnel plot findings: The authors note at the end that they could not address publication bias - but they actually partly did through the funnel plots, which do suggest evidence of bias. The authors should, in their limitations section, instead address the insights from the funnel plots. Skewed funnel plots usually suggest that studies that had small sample sizes with negative findings may not have been published—which might suggest that the effect sizes seen for certain intervention types might be larger than they would be if such studies had been published and were included. If their funnel plots do suggest that small, negative studies might be missing, they should discuss this point for each outcome and in the Discussion section of the paper.
**Response: We have discussed our publication bias findings and how it impacts the interpretation of our findings on lines 512-520 of the discussion section in the statement ‘We observed some evidence of publication bias per the funnel plots among the studies assessing interventions in TB diagnosis and treatment completion . This suggests that the effect size of certain interventions found in under this outcome may have been affected by missing small-size or negative finding studies. The reluctance of academic journals to publish studies with negative findings and our exclusion of case reports and short research reports may have contributed to this bias. Therefore, our reported effects of interventions on multiple care cascade outcomes should be interpreted with caution and within context. However, we corrected this bias by adjusting the plot using the trim and fill method. Therefore, our drawn conclusions based on the meta-anlysis results remain salient..’ We have also stated this shortcoming of our study on lines 403-408 of the limitation paragraph that ‘Fourth, our funnel plots showed the existence of publication bias. This suggests that studies with small sample sizes and studies with negative findings may not have been published. Hence, the effect sizes seen for certain interventions might be larger if such studies had been published and included in the review. Nonetheless, our findings remain relevant to informing TB intervention programming if interpreted with caution and within context.’**

Minor feedback:

1.      Review does not address major gaps in the care cascade: The authors need to be clear that their review does not cover the entire care cascade. For example, although the authors start from "testing" as the first stage, this assumes that patients have made it to clinic sites where testing might be offered. However, across most published country care cascades (e.g., India, South Africa, Zambia, Madagascar, for example), a substantial drop off before testing is partly or mostly attributable to people with TB in the population not even making it to health centers for testing. As such, the authors should be clear that their review does not address this major early problem in the care cascade.

**Response: We agree with the reviewer. We have noted this gaps on lines 540-545 in our limitations paragraph that ‘our study does not cover the entire TB care cascade as gaps in the early stages (focusing on testing or pretreatment loss to follow-up) and post-treatment outcomes (like TB recurrence and death) were not outcomes of interest and were not assessed in this review. Future reviews should consider examining these gaps and other key distinctions (like drug susceptibility or different forms of TB) and their effects on TB care outcomes to help inform strategies and policy adoption.’**

2., the authors do not address post-treatment TB recurrence and death as a problem. Guidelines, including ones cited by the author, and some country care cascades (e.g., India, Madagascar) point out that a meaningful proportion of patient experience post-treatment TB recurrence or death, especially in the year following treatment completion. While much of this gap is attributable to the quality of TB treatment itself (e.g., whether there is high medication adherence), it is very possible that interventions in the immediate time period after TB treatment could help reduce post-treatment death, for example. As such, post-TB care is a major area of interest for the TB community right now. The authors should also note that their review does not address this gap at all.
**Response: We agree with the reviewer that we did not cover post-treatment TB recurrence or death as a problem. We have noted this gaps on lines 540-545 in our limitations paragraph that ‘our study does not cover the entire TB care cascade as gaps in the early stages (focusing on testing or pretreatment loss to follow-up) and post-treatment outcomes (like TB recurrence and death) were not outcomes of interest and were not assessed in this review. Future reviews should consider examining these gaps and other key distinctions (like drug susceptibility or different forms of TB) and their effects on TB care outcomes to help inform strategies and policy adoption’**


Reviewer #4:
RE: PLOS Medicine 22-02722R1 - comments to the authors

The authors have done a very extensive review, with very careful and meticulous analysis including careful assessment of quality, , sources of heterogeneity, publication bias, and notably have applied the full-grade criteria to their findings in order to fully assess the quality of evidence for each intervention and each outcome.

I have a number of suggestions for the authors to consider that I believe would potentially improve the paper and also make the findings a little more accessible, as right now, there is a great deal of information, but I found it difficult to find the most important messages.

1. Introduction: I wonder if they would consider calling this the TB care cascade - a term that is increasingly used.

**Response: We agree with your recommendation and have "TB care continuum" to "TB care cascade throughout the revised manuscript**

2. Also in this section, and later in definitions, they define "linkage to care" as the link between diagnosis and treatment. this is an important linkage but really linkage to care usually refers to the first step - meaning getting individuals to have initial testing. That requires very different approaches compared to linking services within health systems of diagnosis and treatment.

**Response: We agree that linkage-to-care covers more than linkage-to-treatment service post diagnosis. However, we did not focus on the approaches used at the start point of getting people tested. Hence we have qualified our definition to depict the context of our considerations in this review only. We have stated on line 177-179 under the definitions paragraph that "Linkage-to-care in this review refers to the stage in care starting from registering newly diagnosed TB patients at designated facilities to initiate treatment or successfully initiating TB patients on treatment"**

On page 6 "pharmaceutical studies were excluded…." - I could not understand this sentence.

**Response: We have revised this and stated on line 143-146 under the eligibility paragraph that "Studies reporting the use of pharmaceutical interventions (studies that reported change in TB drug regimens or introduced new lines of TB drugs as interventions) were excluded, as recent reviews have evaluated DOTS efficiency in improving TB care outcomes".**

Quality - they have done an excellent job of rating quality, but it is less clear how they have used the quality ratings, (eg in the analysis); this should be stated in the methods.
**Response: We have detailed the quality assessment process and the fact that we used the results of the quality assessment in Grading the quality of evidence using Cochrane GRADE pro on line 207-218 under the methods section.**

Pooling - Top of page 8 "only data...", this sentence is unclear to me and need some clarification.
**Response: We have revised this statement on line 193-194 to “Data from RCTs and non-RCTs with similar comparator arms and intervention strategies compared to standard-of-care were pooled in a meta-analysis using the Review Manager”**

Results - In general, the results are dense; I found it difficult reading through it all to get a clear picture of the most important interventions, particularly ones that will act at a number of different steps in the TB care cascade. I think a summary table, similar in structure/concept to supplemental table 5, would be helpful for the main results. I would like to see a table that summarizes the main interventions and their effect estimates at the main steps in the TB care cascade. An alternative is to have figures which show single interventions and how they act at many different cascade steps. From a programmatic point of view, I think most programs are interested not in correcting a single problem, but rather in the entire TB care continuum, so interventions that act across all steps in the continuum are potentially more valuable than those that act only at a particular step. Hence, some conceptualization, potentially a figure or two showing how some of the major interventions, particularly those that were evaluated at various steps, have produced effects. For example, an intervention that results in more individuals getting tested but has no impact in the number of individuals completing treatment successfully is not necessarily that useful, and in fact is likely to be quite cost inefficient since all the costs are added for diagnostic activities and evaluation of patients result in no real benefit, either to individuals or programs. On the other hand, an intervention that has more modest effects but effects are seen throughout the care cascade, so that more patients are not only diagnosed but actually successfully complete treatment is likely to be much more beneficial intervention. But I cannot really get this from the information as it is now presented.

**Response: Thank your for the recommendation. We have created two new tables (Table 3 and Table 4) which only summarizes significant effects of major interventions which improved outcomes at multiple stages of the TB care cascade.**

Along these lines, were there any studies that looked at multiple steps and could these be another sub analysis, i.e., studies that looked at all steps for a given intervention or studies that looked at multiple steps.

**Response: We have noted in Table 2 that the number of studies that reported on multiple steps for a given interventions was 52. We have further stratified this by the number of studies that reported 2 outcomes, 3 outcomes and than 3 outcomes.**

A minor point is that I think they should round the estimates and confidence intervals to a maximum of one decimal place. To see an estimate of 16.48 with a confidence interval that ranges from near 1 .0 to over 50 is, in my mind, "pseudo precision". It is a minor point but going to one decimal point or even full numbers would be more accurate and would make the tables and figures a bit more readable.

**Response: Thank you for the recommendation. We have decided to maintain the format of two decimal places (which is the norm) for technical statistical interpretation purposes. Rounding up to whole numbers will make findings that were technically not statistically significant (0.91 or 0.55) seem statistically significant. This will introduce additional bias in the interpretation of our findings.**

Finally, but important:
The question about data availability; I do not believe the authors have filled this section correctly. Basically, they say that all data is available in the annex, but this cannot be true. I suggest that their metadata, i.e., really everything abstracted, is placed somewhere that is web-accessible, and the link given in the final version of the manuscript.

**Response: We have followed the Plos Medicine guidelines and made available excel spreadsheets containing relevant data from studies included as part of our supporting documents.**
